# A new Cretaceous thyreophoran from Patagonia supports a South American lineage of armoured dinosaurs

**DOI:** 10.1038/s41598-022-15535-6

**Published:** 2022-08-11

**Authors:** Facundo J. Riguetti, Sebastián Apesteguía, Xabier Pereda-Suberbiola

**Affiliations:** 1 Fundación de Historia Natural Félix de Azara, Centro de Ciencias Naturales Ambientales y Antropológicas, Universidad Maimónides, CONICET, Hidalgo 775, 7mo piso (1405), Buenos Aires, Argentina; 2 Departamento de Geología, Facultad de Ciencia y Tecnología, Universidad del País Vasco/Euskal Herriko Unibertsitatea, Apartado 644, 48080 Bilbao, Spain

**Keywords:** Palaeontology, Palaeontology, Phylogenetics, Taxonomy, Biogeography

## Abstract

The early evolution of thyreophoran dinosaurs is thought to have occurred primarily in northern continents since most evidence comes from the Lower and Middle Jurassic of Europe and North America. The diversification into stegosaurs and ankylosaurs is obscured by a patchy fossil record comprising only a handful of fragmentary fossils, most with uncertain phylogenetic affinities. Here we report the discovery of a new armoured dinosaur from the early Late Cretaceous of Argentina, recovered phylogenetically using various datasets either as a basal thyreophoran or a stem ankylosaur, closely related to *Scelidosaurus*. It bears unusual anatomical features showing that several traits traditionally associated with the heavy Cretaceous thyreophorans did not occur universally. *Jakapil kaniukura* gen. et sp. nov. is the first definitive thyreophoran species from the Argentinian Patagonia. Unlike most thyreophorans, it seems to show a bipedal stance, as in *Scutellosaurus*. *Jakapil* also shows that early thyreophorans had a much broader geographic distribution than previously thought. It is a member of an ancient basal thyreophoran lineage that survived until the Late Cretaceous in South America.

## Introduction

Thyreophora is a clade of ornithischian dinosaurs characterized by the presence of dermal bone armour on their backs^1^. Although most thyreophorans are grouped within Eurypoda (Ankylosauria + Stegosauria), basal thyreophorans show unresolved phylogenetic placements. Traditionally, they have been recovered as non-eurypodan thyreophorans^2–5^. Alternatively, new research suggests them as non-ankylosaurian ankylosauromorphs^6^ (also suggested by Refs.^7,8^). The most representative of these forms are *Scutellosaurus*, *Emausaurus* and *Scelidosaurus*, from the Lower Jurassic of the USA, Germany and England, respectively. *Scutellosaurus* is a small (1.5–2 m body length) and slender thyreophoran with a bipedal life style^9^ whereas the larger *Scelidosaurus* (4.5 m body length) is thought to be facultatively bipedal^10^ (body length has not been confidently estimated for *Emausaurus*). Both *Scutellosaurus* and *Scelidosaurus* represent part of the early locomotor diversity of early ornithischians, with obligate quadrupedality evolving in later and larger ankylosaurs and stegosaurs^9^.

The fossil record of Thyreophora is mainly known from the northern hemisphere, and its presence in the Gondwanan continents remains poorly known, with only a few ankylosaurian or stegosaurian species, and several indeterminate materials^4,11–15^.

We present here a new thyreophoran genus and species from the Late Cretaceous of South America. The remains were found near the locality of Cerro Policía within the ‘La Buitrera Paleontological Area’ (LBPA), close to the E. Ramos Mexía Dam, in North Patagonia, Río Negro Province, Argentina. The outcrops exposed in the LBPA represent the upper section of the Candeleros Formation (Cenomanian). They are interpreted as aeolian accumulations of the Kokorkom Desert (a ~ 826 km^2^ paleoerg^16^), with shifts between arid and semi-arid climatic conditions^17^. Most fossils in the LBPA were found between beds of migrating dunes. The new specimen was found as a close association of elements in a small area (~ 1.5 m × 1 m), isolated from any other individual, as generally occurs in the Candeleros Formation in the LBPA. The remains were found disarticulated, and with a slight southwestern-northeastern orientation due to dune transport.

The LBPA comprises a few localities such as La Buitrera, Cerro Policía, La Escondida and El Pueblito. This rich fossiliferous site produces three-dimensional, largely undeformed fossil vertebrates, including theropod dinosaurs^18^, uruguaysuchid crocodyliforms^19^, eilenodontine sphenodontians^20^, limbed snakes^21^, lizards^22^, chelid turtles^23^, dryolestoid mammals^24^, dipnoans^16^ and undescribed pterosaurs. Fragmentary sauropod skeletons and dinosaur tracks have also been recorded^16^. This new thyreophoran discovery from the LBPA provides new information about the structure of the early Late Cretaceous North Patagonian communities and the role of the still poorly known ornithischian component. In addition, this specimen provides new information about the early diversification and distribution of this cosmopolitan group.

### Institutional abbreviations

AMNH, American Museum of Natural History, New York, USA; BRSMG: Bristol City Museum, Bristol, England; NHMUK: Natural History Museum, London, England; MNA: Museum of Northern Arizona, Flagstaff, USA; MPCA-PV: Colección de Paleovertebrados, Museo Provincial Carlos Ameghino, Cipolletti, Argentina; ZMNH: Zhejiang Museum of Natural History, Hangzhou, China.

## Systematic paleontology

**Figure 1:**
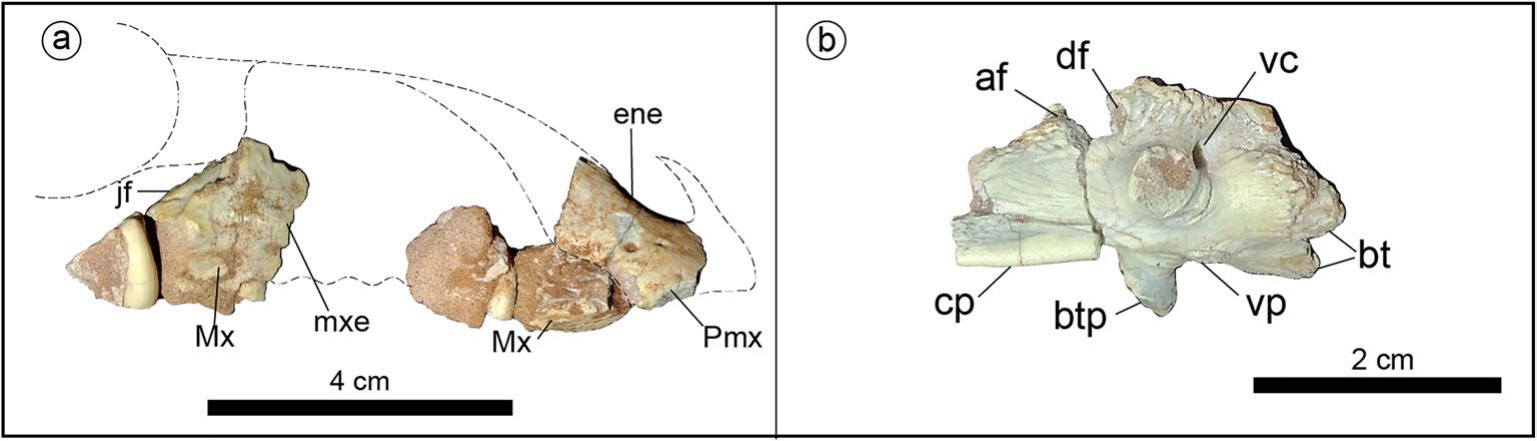
Holotype of *Jakapil kaniukura* (MPCA-PV-630), skull bones. (**a**) Skull bones in right lateral view (dashed contours based on *Scelidosaurus*^10^); (**b**) basisphenoid in left lateral view. *af* anterior foramen, *btp* basipterygoid process, *bt* basal tubera, *cp* cultriform process, *df* double foramen, *ene* external naris edge, *jf* jugal facet of the maxilla, *Mx* maxilla, *mxe* maxillary emargination, *Pmx* premaxilla, *vc* Vidian canal, *vp* ventral process.

**Figure 2:**
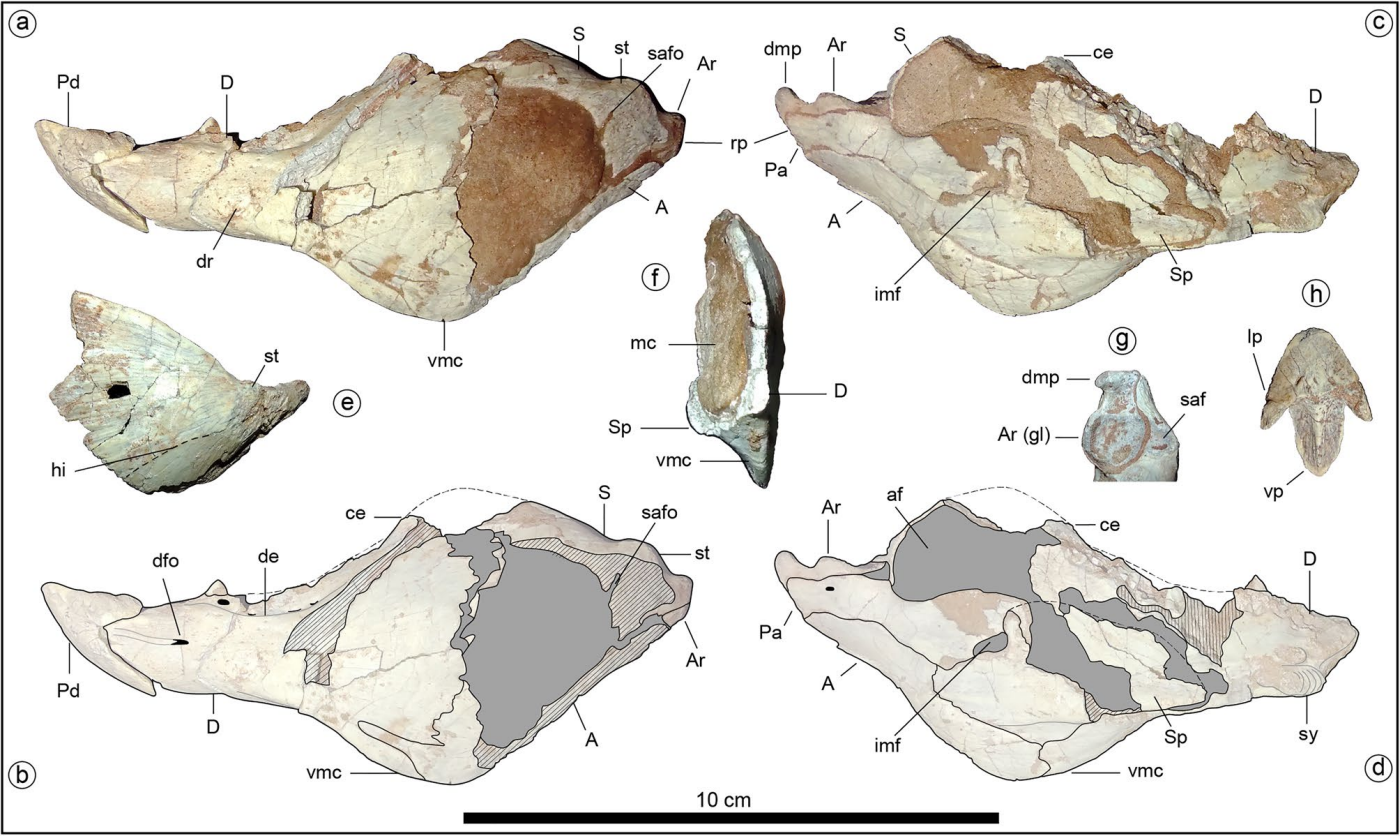
Holotype of *Jakapil kaniukura* (MPCA-PV-630), lower jaw bones. (**a**) left mandible in lateral view; (**b**) left mandible in lateral view, interpreted bone contours; (**c**) left mandible in medial view; (**d**) left mandible in medial view, interpreted bone contours; (**e**) right surangular in lateral view (mirrored); (**f**) transversal section of the posterior half of the left mandible, cranial view; (**g**) articular bone in occlusal view; (**h**) predentary bone in occlusal view. *A* angular, *af* adductor fossa, *Ar* articular, *Ar (gl)* glenoid fossa of the articular, *ce* coronoid eminence, *D* dentary, *de* dentary emargination, *dfo* dentary foramen, *dmp* dorsomedial process of the articular, dr dentary rugosities, *hi* subhorizontal inflection (dashed line), *imf* internal mandibular fenestra, *lp* lateral process of the predentary, *mc* Meckelian canal, *Pa* prearticular, *Pd* predentary, *rp* retroarticular process, *S* surangular, *saf* surangular facet for the glenoid articulation, *safo* surangular foramen (canal), *Sp* splenial, *st* surangular tubercle, *sy* mandibular symphysis, *vmc* ventral mandibular crest.

**Figure 3:**
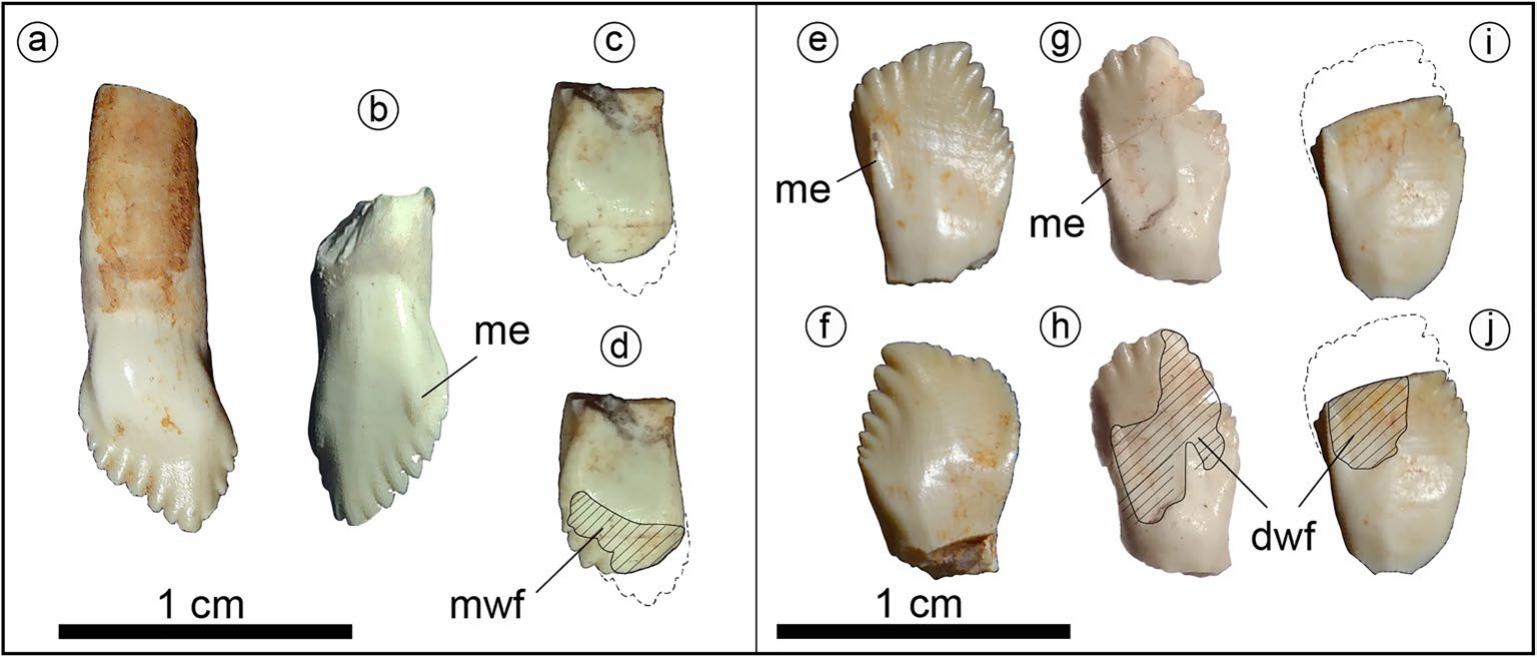
Holotype of *Jakapil kaniukura* (MPCA-PV-630), teeth. Maxillary teeth in labial (**a**,**b**) and lingual (**c**,**d**); (**d**) highlight the wear facet) views; dentary teeth in lingual (**e**,**g**–**j)**; (**h,j**) highlight the wear facets) and labial (**f**) views. *dwf* dentary tooth wear facet, *me* prominent mesial edge, *mwf* maxillary tooth wear facet.

**Figure 4:**
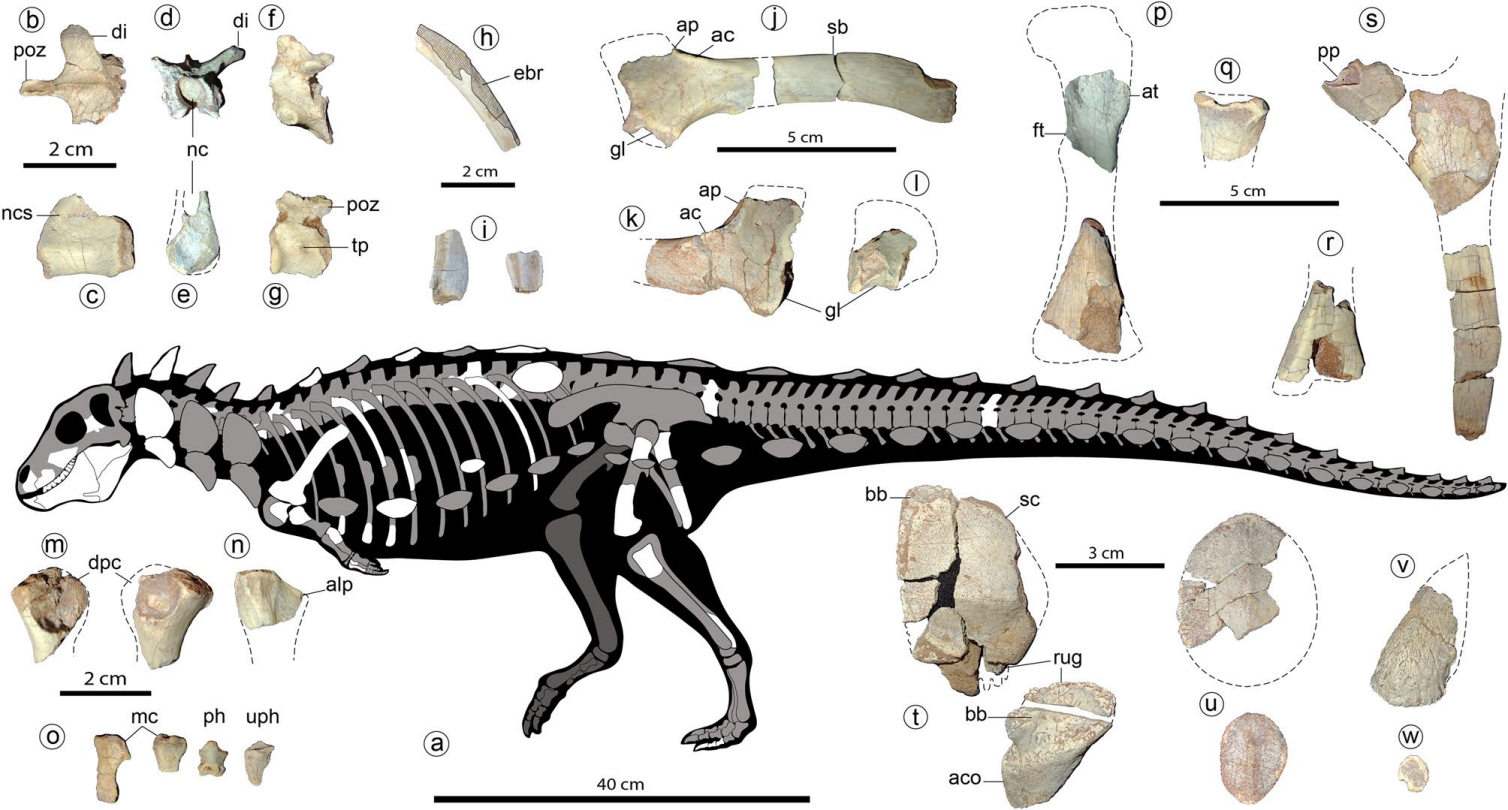
Holotype of *Jakapil kaniukura* (MPCA-PV-630), postcranial bones. Speculative silhouette showing preserved elements (**a**); osteoderm distribution is speculative and partial to show non-osteodermal elements); dorsal vertebra elements in dorsal (**b**), right lateral (**c**) and anterior (**d**,**e**) views; sacral vertebra in left lateral view (**f**); mid-caudal vertebra in left lateral view (**g**); fragment of the mid-shaft of a dorsal rib in posterior view (the enlarged, broken posterior edge is highlighted (**h**); expanded distal ends of two dorsal ribs (**i**); left scapula in lateral view (**j**); right scapula in lateral view (**k**); right coracoid in lateral view (**l**); left and right humeri in anterior view (**m**); probable right ulna in lateral view (**n**); metacarpals, non-ungual and ungual phalanx in dorsal views (**o**); left femur elements in anterior view (**p**); proximal end of the right fibula in lateral view (**q**); distal end of the left tibia in anterior view (**r**); ischial elements in side view (**s**); cervical osteoderms in dorsal view (**t**), flat scutes in dorsal view (**u**), spine-like osteoderm in side view (**v**) and ossicle in dorsal view (**w**). *ac* acromial crest, *aco* asymmetrical cervical osteoderm, *alp* anterolateral process, *ap* acromial process, *at* anterior trochanter, *bb* basal bone, *ebr* expanded broken rib edge, *di* diapophysis, *dpc* deltopectoral crest, *ft* fourth trochanter, *gl* glenoid, mc metacarpals, *nc* neural canal, *ncs* neurocentral suture, *ph* non-ungual phalanx, *pp* pubic peduncle, *poz* postzygapophyses, *rug* marginal rugosities, *sb* scapular blade, *sc* scute, *tp* transverse process, *uph* ungual phalanx.

Dinosauria—Owen, 1842^25^,

Ornithischia—Seeley, 1887^26^,

Thyreophora—Nopcsa, 1915^27^,

*Jakapil kaniukura* gen. et sp. nov. **(**Figs. 1, 2, 3, 4, Suppl. Figs. 2, 3).

### Etymology

The genus, *Jakapil* (Ja-Kapïl: shield bearer), comes from the ‘gananah iahish’, Puelchean or northern Tehuelchean language. The specific epithet, comprising *kaniu* (crest) and *kura* (stone), refers to the diagnostic ventral crest of the mandible, and comes from the Mapudungun language. These languages, currently spoken by more than 200,000 people, have been combined as a tribute to both of the coexisting native populations of North Patagonia, South America.

### Holotype

MPCA-PV-630 is a partial skeleton of a subadult individual (see Supplementary Information) that preserves fragments of some cranial bones (premaxilla, maxilla and basisphenoid), approximately 15 partial teeth and fragments, a nearly complete left lower jaw plus an isolated surangular, 12 partial vertebral elements, a complete dorsal rib and fifteen rib fragments, a partial coracoid, a nearly complete left scapula, a partial right scapula, two partial humeri, a possible partial right ulna, a complete and a partial metacarpal bone, three ischial and two femoral fragments, the distal end of a right tibia, the proximal end of a right fibula, three pedal phalanges, and more than forty osteoderms.

### Referred specimens

MPCA-PV-371, two partial conical osteoderms.

### Locality and horizon

Upper beds of the Candeleros Formation, early Late Cretaceous (Cenomanian, ~ 94–97 My, see^16^, and references therein), locality of Cerro Policía, Río Negro Province, North Patagonia, Argentina (Suppl. Fig. 1).

### Diagnosis

*Jakapil* differs from all other thyreophorans in having: a large, ventral crest on the posterior half of the lower jaw, which is composed of the dentary, the angular and the splenial (medially hidden by the crest); a dorsomedially directed process in the short retroarticular process; leaf-shaped tooth crowns with a prominent mesial edge on their labial surface; maxillary and dentary tooth crowns differ from each other in their apical contour, the former being pointed and strongly asymmetrical, and the latter slightly curved distally with a more rounded and less asymmetrical contour; elongated (articular surface almost or completely beyond the posterior centrum face) and slender (width of less than a half postzygapophyses length) postzygapophyses in dorsal vertebrae; a strongly reduced humerus relative to the femur (proximal humeral width smaller than distal femoral width, see Supplementary Information), with a deep proximal fossa distally delimited by a curved ridge; a very large fibula relative to the femur (anteroposterior length of the proximal end almost comparable to the distal width of the femur); flattened and thin disk-like postcranial osteoderms.

### Summarized description

A detailed description of the holotype is provided in the Supplementary Information. *Jakapil* is a small thyreophoran dinosaur (the subadult holotype is estimated to have been less than 1.5 m in body length and to have weighed 4.5–7 kg; see Supplementary Information, femoral description), with several novelties for a thyreophoran dinosaur.

A short skull is suggested by the size of the skull and jaw bones, and the reduced number of dentary tooth positions (eleven), compared with most non-ankylosaurid thyreophorans^28,29^. The antorbital and mandibular fenestrae seem absent, as in ankylosaurs^29^ (Fig. 1a; the mandibular fenestra is also absent in *Scelidosaurus*^10^). Dentary and maxillary emarginations are present, as usual in ornithischians^30^ (Fig. 1a). The block-like basisphenoid is strongly similar to that of *Scelidosaurus*^10^, with Vidian canals opened posterodorsally to the basipterygoid processes, the basipterygoid processes lateroventrally projected (unlike the anteriorly directed processes of stegosaurs^28^ and ankylosaurs^29^), and a strong cultriform process (as in *Lesothosaurus*^31^, *Thescelosaurus*^32^ and probably *Scelidosaurus*^10^; Fig. 1b).

*Jakapil* also bears the first predentary bone (Fig. 2a–d) with a plesiomorphic shape in a thyreophoran. It is subtriangular and quite similar to that of *Lesothosaurus*^31^, and externally it is ornamented by sulci and foramina, suggesting the presence of a keratinous beak. A beak is also supported in the edentulous and subtly ornamented preserved part of the premaxilla, as in derived thyreophorans^28,29^. The posterior half of the short lower jaw (Fig. 2a–f) is strongly dorsoventrally expanded, resembling the general shape of the heterodontosaurid^33^ and basal ceratopsian jaws^34^. This expansion is composed of a well-developed coronoid eminence (Fig. 2a–d, ce; similar to that in the stegosaur *Huayangosaurus*^35^ and most ankylosaurs^36^) and a large ventral crest at the dentary-angular contact that is unique among thyreophorans (Fig. 2a–d,f, vmc; resembling that of some ceratopsians, see SI). The dentary symphysis is slightly spout-shaped, as in most ornithischians^37^. Anteriorly, the dentary oral margin is subhorizontal in lateral view (Fig. 2a–d, D), unlike the strongly downturned line of most thyreophorans^30,37^. There is no evidence of a mandibular osteoderm as occurs in *Scelidosaurus* and ankylosaurs^10^. A surangular tubercle (Fig. 2a, st) adjacent to the glenoid fossa seems anteriorly continued by a subtly developed subhorizontal inflection of the anterior lamina (Fig. 2e, hi), in the position of the surangular ridge (synapomorphy of Thyreophora^37^), though the first is poorly developed. The glenoid fossa is roughly aligned with the tooth row in lateral view (Fig. 2a–d). The short retroarticular process bears a dorsomedially directed process resembling that of several theropods (Fig. 2g, dmp; see Discussion). This process is absent in all other thyreophorans ^9,10,35,36^.

The tooth crowns are leaf-shaped as in basal ornithischian and thyreophorans^10,28,29,38^ (Fig. 3). The tooth crowns are swollen labially at their base and lack both cingulum and ornamentation, unlike those of derived eurypodans^28,29^, heterodontosaurids^33^ and most neornithischians^30,32^. The mesial edge of the labial surface in the maxillary and dentary tooth crowns is prominent as in *Scelidosaurus*^10^, and ends distally in a denticle-like structure in *Jakapil* (Fig. 3, me). This prominent edge delimits anteriorly the wear facets of the dentary teeth. A striking difference with respect to most thyreophorans is that the maxillary and dentary tooth crowns are quite different (see Supplementary Information). The maxillary teeth (Fig. 3a–d) show seven/eight mesial and four distal denticles, a vertical apical denticle, and a straighter mesial denticle row (resembling those of non-ankylosaurid and non-stegosaurid thyreophorans^10,35,36^). The dentary teeth (Fig. 3e–j) bear seven mesial and five/six distal denticles, and a distally curved apical-most denticle. Also, the mesial denticle row is lingually recurved, as in *Huayangosaurus*^35^. Large, high-angled wear facets are present (Fig. 3d,h,j; dwf and mwf).

The axial elements are similar to those of *Scelidosaurus*^39^ (Fig. 4). The posterior articular surface of an isolated cervical centrum is flattened and seems almost as wide as high. A large foramen is placed just posteroventral to the parapophysis. The dorsal centra are cylindrical and elongated, with subcircular articular surfaces, and are biconcave (Fig. 4c,e). The neural arch is low but the neural canal is larger (Fig. 4d,e, nc). A dorsal neurocentral suture is visible (Fig. 4c, ncs). The diapophyses are laterodorsally directed almost 40° from the horizontal (Fig. 4d, di), at a lower angle than in stegosaurs^28^ and most ankylosaurs^29^, unlike the horizontal processes of basal ornithischians^38^. The postzygapophyses are medially fused in a slender (width of less than a half postzygapophyses length) and strongly elongated posteriorly structure (Fig. 4b, poz; more than in some ankylosaurs, such as *Euoplocephalus* and *Polacanthus*; see^40,41^). An isolated mid-caudal vertebra shows an equidimensional centrum in lateral view, with concave, oval articular surfaces (Fig. 4g). Transverse processes are very small and button-like (Fig. 4g, tp). Postzygapophyses are medially fused and do not extend beyond the centrum edge (Fig. 4g, poz). Proximally, the cross-section of the dorsal ribs is T-shaped. The low curvature of the shaft suggests a wide torso, as occurs in *Emausaurus*^42^, *Scelidosaurus*^39^, and ankylosaurs^29^. Some rib fragments with expanded (though broken) posterior edges suggest the presence of intercostal bones (Fig. 4h, ebr), as in *Scelidosaurus*^39^, *Huayangosaurus*^43,44^, some ankylosaurids^45^ (and references therein) and some basal ornithopods^46^. Some ribs are distally expanded (Fig. 4i) like the anterior dorsal ribs of *Scelidosaurus*^39^ and *Huayangosaurus*^43^.

Girdle and limb bones (see also Suppl. Figs. 2, 3) are mostly broken and with boreholes (probably due to bioerosion) at their ends. The scapular blade (Fig. 4j, sb) is elongated and parallel-sided, without distal expansion, an overall shape that resembles that of several theropods^47^, contrasting the distally expanded condition in most ornithischians^30^. A straight and parallel sided scapular blade is common in ankylosaurids^29,40^. The proximal scapular plate with a high acromial process (Fig. 4j,k, ap) is stegosaurian-like, and the lateral acromial crest (Fig. 4j,k, ac) is developed as in *Huayangosaurus*^43^. A low distinct ridge rises posterior to the glenoid fossa and represents the insertion site for the muscle *triceps longus caudalis*, as occur in ankylosaurids ^40^. The incomplete coracoid (Fig. 4l) is much shorter than the scapula, unlike that of ankylosaurs^29,40^, which bear a large coracoid. The coracoid and the scapula are not fused. The partial humeri (Fig. 3m) are strongly reduced in size, with overall limb proportions resembling those of basal ornithischians^3,38^ and several theropods^47^. A possible proximal end of the ulna (Fig. 4n) resembles that of other basal ornithischians, though more strongly laterally compressed. The anterolateral process is present (Fig. 4n, alp), and the olecranon process seems absent or poorly developed, as in *Scutellosaurus*^9^ and *Scelidosaurus*^39^. The ischia are poorly preserved (Fig. 4s). The pubic peduncle is separated from the iliac articulation, unlike the continuous cup-shaped structure of most ankylosaurs^29^. The shaft of the ischium is straight and parallel-edged, as in *Scutellosaurus*^9^ and *Scelidosaurus*^39^, and distally tapers as in stegosaurs^28^. The preserved femoral pieces (Fig. 4p) resemble those of basal ornithischians^38,39^. The bases of both the broken anterior and fourth trochanters (Fig. 4p, at, ft) are large, suggesting large elements; the fourth trochanter is proximally placed on the femoral shaft (near the height of the base of the anterior trochanter); and the distal end of the femur is slightly curved posteriorly. The proximal end of the right fibula (Fig. 4q) is much larger than that of all other thyreophorans (compared with both the femoral and tibial distal ends) and bears a large anterior curved crest. The block-like non-ungual phalanges and a bluntly pointed hoof-like ungual (Fig. 4o, ph, uph) are similar to those of *Scelidosaurus*^39^.

At least five osteoderm types are preserved in the holotype of *Jakapil*. The cervical elements are composed of an external, low-crested scute (Fig. 4t, sc) over a fused, smooth bone base (Fig. 4t, bb), as in *Scelidosaurus*^48^ and several ankylosaurs^2,49^. A probable cervical element is also composed of a concave base of smooth bone fused to a high, asymmetrical osteoderm (Fig. 4t, aco). The bases of these dermal elements present strong rugosities at one edge, suggesting a sutural contact between (Fig. 4t, rug), as in *Scelidosaurus*^48^ and some ankylosaurs (such as *Pinacosaurus* and *Scolosaurus*^40,49,50^). Scute-like post-cervical osteoderms (Fig. 4u) are strongly flattened, disk-shaped, and suboval with a very low crest, resembling those of few ankylosaurs such as *Gastonia* and *Gargoyleosaurus*^51^ (‘body osteoderms’ sensu Kinneer et al.^52^; see also^49^). Only one scute shows a high triangular cross-section like those of *Scelidosaurus*^48^. Also present are a few conical, spike-like osteoderms with deep concave bases (Fig. 4v), and many flat, disk-shaped, minute (7–10 mm) ossicles without crests (Fig. 4w).

### Phylogeny

**Figure 5:**
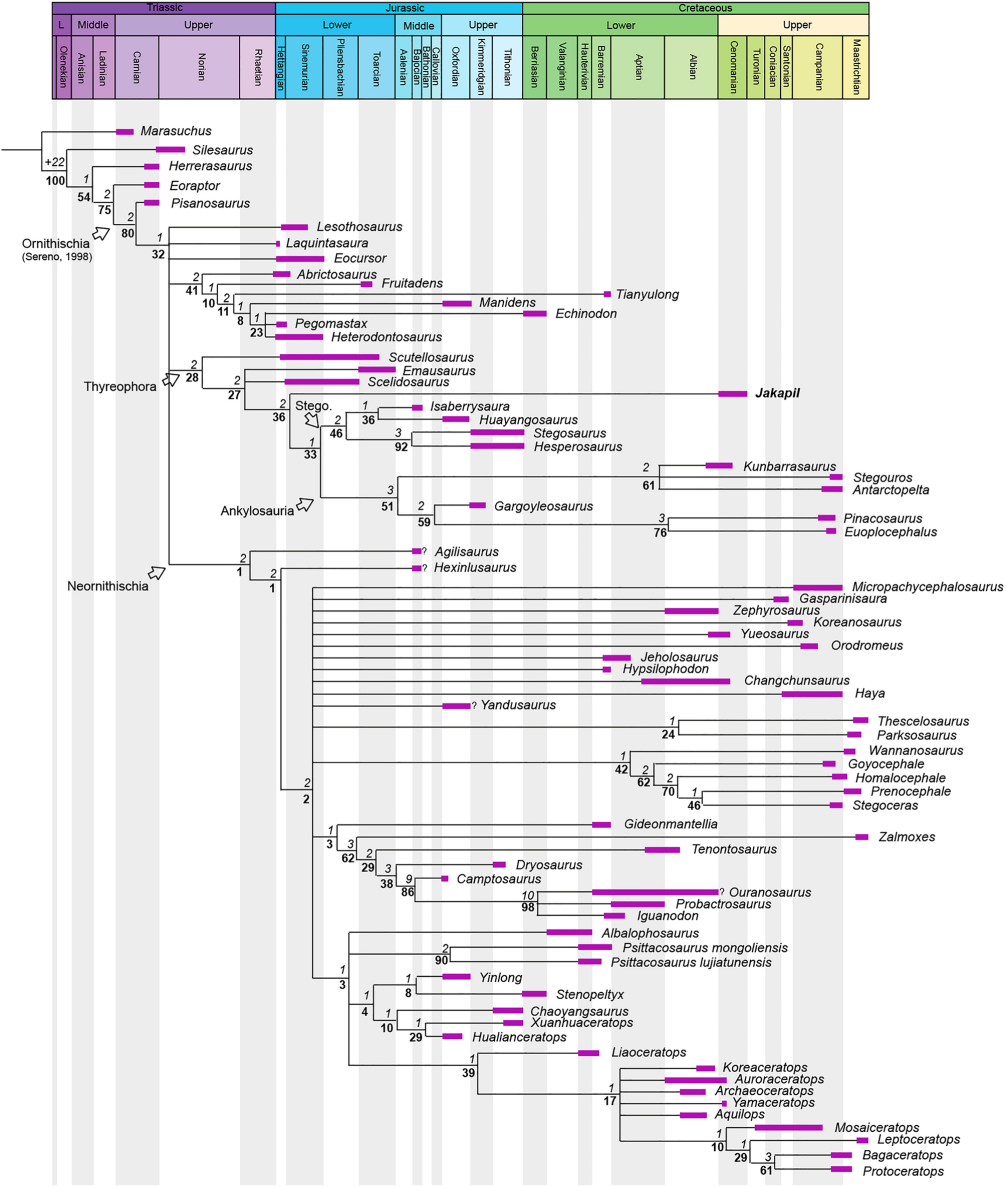
Time-calibrated strict consensus of 26,784 most parsimonious trees (L = 1267) with the Soto-Acuña et al.^5^ matrix. CI 0.359, RI: 0.708. Branch supports are figured (Bremer/bootstrap). Record ages references are listed in the Supplementary Information (Suppl. Fig. 4).

The phylogenetic analysis using the matrix of Soto-Acuña et al.^5^ recovers *Jakapil* within Thyreophora, as the sister taxon of Ankylosauria (Fig. 5). The branch support for the basal thyreophorans is considerably lower than that obtained by Soto-Acuña et al.^5^, although the support of Stegosauria and some less inclusive eurypodan clades is slightly better (ceratopsians and pachycephalosaurs also show a lower support). The *Jakapil* autapomorphies in this analysis are: ventrally orientated basipterygoid processes (char. 134; shared with *Agilisaurus*, *Hypsilophodon*, *Zalmoxes*, *Tenontosaurus*, *Dryosaurus*, *Liaoceratops*, *Yamaceratops*, *Leptoceratops*, *Bagaceratops* and *Protoceratops*); lateral orientation of the basipterygoid process articular facet (char. 136; shared with *Homalocephale*, *Prenocephale*, *Stegoceras* and *Yinlong*); a straight dentary tooth row in lateral view (char. 166; shared with the ornithischians *Lesothosaurus*, *Eocursor*, *Scutellosaurus*, *Pinacosaurus*, *Euoplocephalus*, heterodontosaurids and neornithischians); the presence of a ventral flange on the dentary (char. 170; shared with *Psittacosaurus, Yamaceratops* and *Protoceratops*); a well-developed coronoid process (char. 174; shared with heterodontosaurids and neornithischians); a surangular length of more than 50% the mandibular length (char. 183; shared with *Stegoceras*, *Psittacosaurus, Yinlong, Chaoyangsaurus* and *Hualianceratops*); less than 15 dentary teeth (char. 204; shared with heterodontosaurids, *Gasparinisaura*, *Hypsilophodon*, *Wannanosaurus*, *Tenontosaurus*, *Dryosaurus* and ceratopsians); apicobasally tall and blade-like cheek teeth crowns (char. 205; shared with *Laquintasaura*, *Psittacosaurus, Yinlong, Chaoyangsaurus* and *Hualianceratops*). Alternative phylogenetic analyses using the data matrices of Maidment et al.^4^, Norman^6^ and Wiersma and Irmis^8^ recover *Jakapil* as the sister taxon of Eurypoda (Stegosauria + Ankylosauria) and as a basal ankylosaur, respectively (see Supplementary Information). Being recovered either as an ankylosauromorph or a stem-eurypodan, *Jakapil* is closely related to *Scelidosaurus* in all analyses. Detailed phylogenetic results and discussion are provided in the Supplementary Information.

## A novel thyreophoran anatomy

The discovery of *Jakapil* in the Cenomanian of Argentina shows a completely new thyreophoran lineage for the Southern Hemisphere. The new taxon shares many features with basal ornithischians and thyreophorans (even with ankylosaurids, see Supplementary Information), but also bears several novelties. The relatively short mandible of *Jakapil* (Fig. 2a–d), with a large adductor fossa, extensively ornamented surangular, and a well-developed coronoid eminence (even higher than that of *Scelidosaurus*), resembles that of heterodontosaurids^33^ and basal ceratopsians^34^, suggesting a quite strong bite for a thyreophoran^36,53^. The wear facets of *Jakapil* (Fig. 3, dwf, mwf) indicate dental occlusion; they are larger than those of most basal thyreophorans and stegosaurs, resembling those of the adult lectotype of *Scelidosaurus*^10^, ankylosaurs, ceratopsids and hadrosaurids^36,54^. *Scelidosaurus* shows a patched arrangement of wear along the tooth rows^10^. In *Jakapil*, the wear on functional cheek teeth is large and high angled, and seems largely distributed along the tooth rows, from the anteriormost teeth backward, as in heterodontosaurids and cerapodans^33,54^. On the whole, it is probable that *Jakapil* had a masticatory system that was more efficient than the early thyreophorans in processing vegetation. The *en echelon* arrangement of tooth crowns suggests a mainly orthal motion for chewing, as in most thyreophorans^6,36,53^ (and references therein). In *Scelidosaurus* and other thyreophorans, the ventrally curved tooth row allows a ‘scissor’ effect of the anteriormost teeth^10^. By contrast, the straight, narrow snout of *Jakapil* suggests a different feeding strategy, not cutting leaves but selecting elements that require harder processing^36^. Thus, *Jakapil* expands the record of herbivorous vertebrates from the Kokorkom paleodesert, complementing this trophic level that contains the lepidosaur *Priosphenodon*^20^.

The predentary bone is the first known for a basal thyreophoran. Despite the variably complete lower jaws in *Scutellosaurus*^9^, *Emausaurus*^42^, *Scelidosaurus*^10^, “*Tatisaurus*”^55^ and “*Bienosaurus*”^56^, a predentary bone is absent. Norman^10^ suggested that this may be cartilaginous in *Scelidosaurus*. The presence of an ossified predentary bone in *Jakapil* contrasts with that hypothesis. Its plesiomorphic morphology mainly resembles that of the basal ornithischian *Lesothosaurus*^31^. It also shows some similarities with the predentary of stegosaurs^35^ and is very unlike the broad predentary of ankylosaurs^29^, suggesting a more selective food strategy^36^. Several features of the predentary, such as the large ventral process, are shared with some basal ceratopsians^34^. However, this process is usually very robust and proportionally larger that the lateral processes in basal ceratopsians when comparing with *Jakapil*.

The presence of a dorsomedial process in the articular (Fig. 2c,d,g, dmp) is a new component in a thyreophoran jaw. Some ankylosaurids bear a medial shelf of the glenoid formed by a medial expansion of the articular^36^. In *Jakapil*, the glenoid fossa is not medially extended, and the pointed dorsomedial process arises from the retroarticular process. A rather similar process is present in various coelurosaurian theropods, such as dromaeosaurids^57^, *Tyrannosaurus*^58^, *Gobipteryx*, and ornithurine birds^59^. In Neornithes, the dorsomedial process of the articular is more anteriorly placed, medial to the mandibular articulation (F. J. R., pers. obs. based on specimens in the Fundación Azara collection: *Bubo*, *Guira*, *Pterocnemia* and *Eudromia*; see also^60^), and receives the pterygoid adductor musculature^61^. In living crocodilians and lepidosaurs, the pterygoid musculature is usually attached to the posteroventral edge of the mandible (also inferred for non-avian dinosaurs^61^). However, the presence of a medial process in *Jakapil* may suggest a new placement of the pterygoid musculature, as in birds. If this were the case, the free ventral crest of the mandible (Fig. 2a–d,f, vmc) could have had an exhibition function, rather than being used as an enlarged musculature attachment. In fact, the rugged texture across the mandibular edge resembles an ornamentation element (as in *Scelidosaurus*^10^ and *Pinacosaurus*^29^; and references therein) with no obvious muscular scar. Otherwise, the crest may represent an enlargement of the surface for musculature insertion, increasing the efficiency of the chewing process (see above).

The armour of *Jakapil* is also peculiar. Almost all the recovered osteoderms are extremely low, unlike those of basal thyreophorans (Fig. 4r,s,u). The Morphotype A osteoderms of *Scutellosaurus*^62^ are very low and bear a central keel, being roughly similar to the disk-shaped osteoderms of *Jakapil* (Fig. 4s; although the keel of the osteoderms in the latter is smoother). Larger scutes in *Jakapil* show twice the radial extension of those of *Scutellosaurus*. Some ankylosaurs, such as *Gastonia*^52^ and *Gargoyleosaurus*^51^, bear depressed plate-like osteoderms (with or without a low, sharp keel) resembling those of *Jakapil*. In *Scelidosaurus*, the osteoderms develop a strong keel^48^, much higher than that seen in the *Jakapil* osteoderms. By contrast, large, high-keeled osteoderms and spikes are rare in *Jakapil*.

The above-mentioned features appear to be novelties, probably due to the poorly known record of thyreophorans in the Southern Hemisphere^4,11–15^. Moreover, the mixture of plesiomorphic, stegosaurian and ankylosaurian characters of *Jakapil* may also suggest a basal phylogenetic placement (outside Eurypoda) for this taxon (contra^6^). In addition, the incorporation of *Jakapil* into the data matrices of Soto-Acuña et al.^5^, Norman^6^, Maidment et al.^4^, and Wiersma and Irmis^8^ generates a general decrease in branch support. This demonstrates that the early diversification of thyreophorans is still poorly understood due to their poor Lower-Middle Jurassic fossil record and the scarcity of Gondwanan material^4,11^, and may explain the ambiguous phylogenetic placement of *Jakapil* and the early thyreophorans^2,4,6^.

## Bipedalism in armoured dinosaurs

Regarding locomotion, the evolutionary trends observed in thyreophorans are associated with the transition between small, bipedal species and large or graviportal quadrupedal forms, observed in Ankylosauria and Stegosauria^1^. The transitional state has been attributed to the facultative quadruped *Scelidosaurus*^39^. In *Jakapil*, the relative dimensions of the forelimb, hind limb, and cranial remains (Fig. 4a) bear a greater resemblance to those of the bipedal theropods^47^, basal ornithischians^38^ and heterodontosaurids^33^ than thyreophorans. Moreover, the elongated, non-expanded scapular blade and the strong reduction in the humeri resemble those of specific theropod clades (e.g., abelisaurids^47^), and unlike the shorter, distally expanded scapular blade of the fully quadrupedal ornithischians and sauropods.

A comparison of the limb elements of some thyreophorans (Suppl. Fig. 4) shows the strong reduction in size of the humerus in *Jakapil*. Considering a reconstruction of the elements based on *Scelidosaurus* (the nearest taxon to *Jakapil* in all phylogenetic analyses), *Scutellosaurus* (a basal form) and *Jinyunpelta* (an ankylosaur), the reduction in size is evident. Despite the incompleteness of the material, we quantified this reduction comparing the proximal humeral (PHW) and the distal femoral widths (DFW; the distal end of the femur in *Jakapil* was measured in the only well-preserved transversal section, although this is not the most distal). The proximal humeral width/distal femoral width ratio (HFR) is lower in the basal taxa (*Jakapil*, *Scutellosaurus* and *Scelidosaurus*) with respect to the ankylosaurs *Jinyunpelta* and *Euoplocephalus*, showing a widening of the humerus in the quadrupedal taxa reaching a comparable width (ratio ~ 1). Such widening in the proximal humeral end is evident in the lack of fit of the *Jakapil* bones in the *Jinyunpelta* proportions, also suggesting limb proportions more similar to those of basal forms. Moreover, the incomplete distal end of the femur in *Jakapil* allows even smaller values of the HFR ratio (and of the humerus size). Also, a shortening of the humerus relative to the femur is present in the obligate quadrupedal Ankylosauria. Regardless of the unknown humeral length, the lack of a robust humerus in *Jakapil* allows us to reject a fully quadrupedal stance like that of the heavily built ankylosaurs.

In summary, the overall limb dimensions and estimations (with forelimb and olecranon process both reduced^63^), and the femoral anatomical similarities to the basal ornithischians and thyreophorans^39^ (e.g., large trochanters and a non-columnar element) suggest a bipedal stance in the specimen. However, the incompleteness of the remains demands caution to define the stance of *Jakapil*. To make more complex the scenery, *Jakapil* still retains quadruped-associated features, such as a probable anterolateral process in the ulna, and stout metacarpals^63^ (and references therein). More complete material is needed to make accurate quantitative comparisons with other taxa and clarify its stance.

## Paleobiogeography of early thyreophorans

The Early Jurassic thyreophoran record consists of basal forms known from several continents showing a Pangean distribution. These comprise *Scutellosaurus* (Hettangian-Toarcian of the USA^9^), *Scelidosaurus* (Sinemurian of the UK; see ^6,10^), *Emausaurus* (Toarcian of Germany^42^), ‘*Lusitanosaurus*’ (Sinemurian of Portugal^1^), ‘*Bienosaurus*’, ‘*Tatisaurus*’ (both from the Hettangian-Sinemurian of China; see^55,56^) and *Yuxisaurus* (late Sinemurian-Toarcian^64^). Some recent papers recover the unarmoured ornithischians *Lesothosaurus* (Hettangian-Sinemurian of South Africa and Lesotho; see^3,31^) and *Laquintasaura* (Hettangian of Venezuela; see^3,4,65^) as Gondwanan basal members of Thyreophora. However, alternative studies show their phylogenetic position as controversial^3,6,65^, so they cannot be confidently assigned to Thyreophora.

The extensive distribution of armoured basal thyreophorans (excluding both *Lesothosaurus* and *Laquintasaura*) across the northern landmasses during the Early Jurassic shows a rapid diversification after the origin of the clade. However, basal thyreophoran remains from Gondwana known from the Middle Jurassic of Niger^66^, along with problematic material from the Lower Jurassic (Sinemurian-Pliensbachian) of India (see^55,67^ and references therein), suggest a more extensive distribution for the early thyreophorans. In addition, the early distribution of stegosaurs and ankylosaurs shows a similar pattern. The presence of the Middle Jurassic basal stegosaur *Isaberrysaura* from the Bajocian of Argentina^68,4^ and the stegosaurid *Adratiklit* from the Bathonian-Callovian of Morocco^4^ depict a distribution of the early stegosaurs that extends into southern landmasses. The Middle Jurassic thyreophoran fossil record also includes the stegosaur *Loricatosaurus* from the Callovian of England and France, the ankylosaurs *Sarcolestes* and ‘*Cryptosaurus*’ from the Callovian of England, the ankylosaur *Spicomellus* from the Bathonian-Callovian of Morocco^15^, the ankylosaur ‘*Tianchisaurus*’ from the Callovian of China, and indeterminate remains from Europe and Asia (see^4,67^ and references therein). On the whole, the Pangean distribution of early thyreophorans across the Early-Middle Jurassic makes it difficult to recognize a source area for Thyreophora and the most inclusive clades within it.

In this context, *Jakapil* not only increases the poor Gondwanan record of thyreophorans, but also establishes a theoretical framework for Gondwanan basal thyreophoran evolution and distribution (Fig. 5). Whether *Jakapil* is recovered as an ankylosaur or a non-eurypodan thyreophoran, it is closely related to *Scelidosaurus* (see Supplementary Information). The presence of a basal thyreophoran in the early Late Cretaceous of South America shows that an ancient Gondwanan lineage of early thyreophorans evolved independently from those of the Northern Hemisphere, whose relationships have to be traced during the Early-Middle Jurassic Pangean rupture and the consequent isolation of Gondwana and later South America. Accordingly, early Gondwanan thyreophorans survived a long time after the Pangean breakup, whereas the northern early thyreophorans seem to have gone extinct by the Middle Jurassic. The persistence of a Gondwanan lineage of thyreophorans finds support in new thyreophoran remains recovered from the Lower Cretaceous rocks of the Bajada Colorada Formation (Berriasian-Hauterivian), also from the Argentinian Patagonia (Neuquén Province). These remains are composed of a diversity of osteoderms, showing either a mixture of thyreophorans at the site, or a new, still poorly understood lineage^69^.

A new lineage of Gondwanan thyreophorans was recently proposed by Soto-Acuña et al.^5^, Parankylosauria. This clade includes *Antarctopelta* (Campanian-Maastrichthian of Antarctica), *Stegouros* (Campanian-Maastrichthian of South America), and the traditionally basal ankylosaur *Kunbarrasaurus* (Albian-Cenomanian of Australia)^5^. Despite their extensive Gondwanan distribution during the Cretaceous, anatomical differences with *Jakapil* are remarkable. Parankylosaurs show ankylosaurian features, like broad ornamented skulls, depressed caudal vertebrae, similar limb proportions, and a columnar femur with both reduced anterior and fourth trochanters (among others), which contrast with the mixture of features of *Jakapil*. Even more, ankylosaurian features present in *Jakapil* were recognized as convergent with ankylosaurids within Euankylosauria (e.g., a straight dentary tooth row in lateral view, a small diastema on the dentary, a shallow symphysis, scapular blade shape; see Supplementary Information) rather than with parankylosaurs. Therefore, a close phylogenetic relation between them is unlikely. Until more records contribute to the understanding of Gondwanan thyreophorans, both *Jakapil* and parankylosaurs belong to two different lineages. This shows that Gondwanan thyreophorans were a diverse clade with morphologically disparity.

On the other hand, further work will help to fill the extensive gap between the early thyreophorans and the Cretaceous remains from South America. Recent research is reflected in an increase in the thyreophoran fossil record from South America, with all its implications for thyreophoran evolution^11–14,69,70^; and this paper). The discovery of *Jakapil* not only supports the presence of a new Gondwanan lineage of early thyreophoran dinosaurs that persisted in Gondwana for a long time, but has also brought to light the importance of the Gondwanan fossil record in the study of the origin and evolution of dinosaurs (and other clades).

## Methods

### Morphological datasets

We used the dataset of Soto-Acuña et al.^5^, that comprises a broad sample of ornithischians suitable to test the phylogenetic position of *Jakapil*, a specimen with a complex mixture of features complete enough to include both several outgroups (non-thyreophoran ornithischians) and also both groups of thyreophorans. The dataset consists of 75 taxa and 383 morphological characters (see character list in Soto-Acuña et al.^5^ and references therein). *Marasuchus* was fixed as the outermost outgroup taxon. All characters were unweighted. Characters 2, 23, 31, 39, 125, 163, 196, 203, 204, 222, 227, 238, 243, 247, 268, 292, 296, 302, 306, 320 and 361 were treated as additive. Memory space was made for 1,500,000 trees.

### Phylogenetic analyses

Phylogenetic analyses of the morphological matrix were carried out in TNT v1.5 (see Supplementary Information). A Traditional search was applied with 10,000 replicates of Wagner trees under the tree bisection reconnection (TBR) algorithm, saving 10 trees per replication. Trees saved in memory were resampled with an additional round of TBR. The support for each node in the trees was assessed in TNT. Bremer values were also recorded with Traditional searches until 22 suboptimal trees. Bootstrap analysis was carried out using 10,000 pseudoreplicates with a Traditional search, and Absolute frequencies. Consistency and retention indexes (from the archive STATS.RUN), character mapping, and moving taxon positions over the consensus to test parsimony, were carried out in TNT. The detailed phylogenetic methods are provided in the Supplementary Information.

## Supplementary Information

Supplementary Information.
