# Supplementary material for: A new Cretaceous thyreophoran from Patagonia supports a South American lineage of armoured dinosaurs
Source: Sci Rep. 2022 Aug 11;12:11621. doi: 10.1038/s41598-022-15535-6 (PMC9372066; doi:10.1038/s41598-022-15535-6)
Supplement: Supplementary file 1 — Supplementary Information. [file 41598_2022_15535_MOESM1_ESM.docx]

**A new Cretaceous bipedal thyreophoran from Patagonia supports a South American lineage of armoured dinosaurs**

Facundo J. Riguetti, Sebastián Apesteguía, Xabier Pereda-Suberbiola

**Supplementary Information**

**Contents**

[Geological and paleontological settings 2](#_Toc73951777)

[Description of *Jakapil kaniukura* 4](#_Toc73951778)

Histology and ontogenetic state…………………………………………………………………………………………………………….17

[Testing the phylogenetic placement of *Jakapil kaniukura* 22](#_Toc73951779)

[Supplementary References 35](#_Toc73951781)

Morphological data matrices ………………………………………………………………………………………………………………..48

**Institutional abbreviations**

BRSMG: Bristol City Museum, Bristol, England; NHMUK: Natural History Museum, London, England; MPCA-PV: Colección de Paleovertebrados, Museo Provincial Carlos Ameghino, Cipolletti, Argentina.

# Geological and paleontological settings

*Jakapil* *kaniukura* (MPCA-PV-630) was found in the early Upper Cretaceous Candeleros Formation. This continental unit belongs to the Neuquén Group deposited within the Neuquina Basin, and crops out extensively in several localities at the central-eastern region of the Neuquén Province and the northwestern region of Río Negro Province, around the E. Ramos Mexía Dam (Supp. Fig. 1a). The Candeleros Formation comprises an almost 200 m sequence of reddish psammite in muddy matrix deposits, with some intercalations of tuff or psephites. These are interpreted as fluvial deposits that may be associated with terminal fans, playa-lakes, and sandy dunes (Garrido, 2010). The age is considered as Cenomanian-Turonian, as constrained between the ^~^99 Ma Patagonidican Unconformity (Leanza, 2009) and the 88±3 Ma base of the overlying Huincul Formation (Corbella *et al.*, 2004).

The upper section of the Candeleros Formation outcrops at the La Buitrera Paleontological Area (LBPA), in the western slope of the Rentería plateau, Río Negro Province, North Patagonia, Argentina. The coarse to medium-grained sandstones and subordinate conglomerates at the LBPA represent the aeolian accumulations of the Kokorkom desert (an at least 826 km^2^ paleoerg; Candia Haluckzop *et al.*, 2018). Three described stages of respective contraction and expansion of the erg through time resulted in changes in the water table position and water availability, following the shifts between arid and semi-arid climatic conditions (Pérez Mayoral *et al.*, 2021). Most of the fossils of the LBPA were found isolated and articulated, in seasonally migrating aeolian dunes, represented by thick cross-bedded sandstone beds, some with sides of stabilized paleosols, rhizoliths and burrows (Candia Haluckzop *et al.*, 2018).


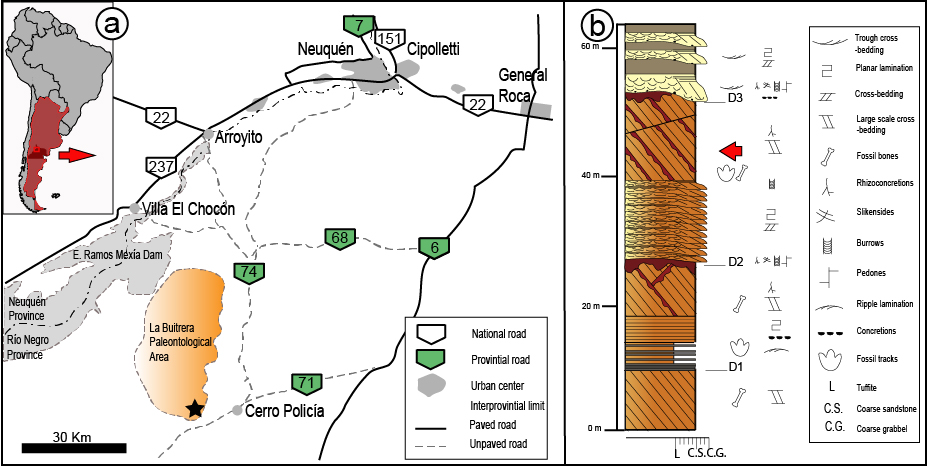


**Supplementary Figure 1.** Location and geology of the Cerro Policía locality. **a**, Geographic location of the La Buitrera Paleontological Area (LBPA): South America (top left; Argentina and Río Negro Province colored), and detail of the northern Rio Negro Province with the LBPA colored (right). Black star indicates the type locality of the specimen; **b**, Stratigraphic section of the Candeleros Formation at the Cerro Policía locality (modified from Candia Haluckzop *et al.*, 2018). Red arrow in **b** indicates the stratigraphic location of the specimen.

The LBPA comprises some few localities like La Buitrera (main locality; see Apesteguía & Novas, 2003), Cerro Policía, El Loro, La Escondida, and El Pueblito, with a rich and unique fossil record of vertebrates. In several sites of the Candeleros Formation there were found large and giant vertebrates, including both carcharodontosaurid and titanosaurian dinosaurs (Leanza *et al*., 2004). However, the particular conditions of the LBPA allowed the preservation of a large number of small, mostly articulated and uncrushed vertebrates. As mentioned in the main text, the vertebrate fossil record includes dromaeosaurid and alvarezsaurid dinosaurs (Makovicky *et al.*, 2005; 2012), undescribed pterosaurs, uruguaysuchid crocodyliforms (Pol & Apesteguía, 2005; Fernández Dumont *et al.*, 2020), eilenodontine sphenodontians (Apesteguía & Novas, 2003), limbed snakes (Apesteguía & Zaher, 2006; Garberoglio *et al.*, 2019a; 2019b), lizards (Apesteguía *et al.*, 2005), chelid turtles (Maniel *et al.*, 2020), dryolestoid mammals (Rougier *et al.*, 2011), and ceratodontiform dipnoans (Apesteguía *et al.*, 2007). Fragmentary sauropod skeletons and dinosaur tracks were also recorded (Candia Haluckzop *et al.*, 2018).

The holotype and referred materials of *Jakapil* *kaniukura* were found in the upper beds of the Candeleros Formation (Cenomanian, ~94-97 My; see Candia Halupczok *et al*., 2018, and references therein) at the ‘Rinconada de la Piedra Blanca’ and ‘Jofra’ sites, respectively, near the locality of Cerro Policía within the LBPA, Río Negro Province, North Patagonia, Argentina (Suppl. Fig. 1a, b). The holotype individual was found isolated, with all elements associated but disarticulated (unlike most fossils in the LBPA). The material was found in the lower part of a thick (~10 m) bed composed of gray to yellowish, well sorted sandstone, interpreted as migrating dune deposits, just below the D3 discontinuity of Candia Haluckzop *et al.* (2017). These levels of the Candeleros Formation in the locality of Cerro Policía have been interpreted in the context of aeolian-fluvial interactions at the southern margin of the Kokorkom Desert (Candia Haluckzop *et al.,* 2017; Pérez Mayoral *et al.*, 2021). Conversely, no remains of *Jakapil* have been found at the centre of this paleoerg system in the locality of La Buitrera (~10 km northward), after two decades of fieldwork. The presence of this herbivore dinosaur near the margins of the desert and its absence at the centre are assumed to be due to the higher availability of food sources. New research will help to improve the geographic distribution of *Jakapil* and its ecological importance.

# Description of *Jakapil* *kaniukura*

All elements are figured in the main text and Supplementary Figures 2 and 3.

**Skull overview.** Most elements of the skull are absent. However, preserved bones allow some comments. The preserved premaxilla, maxilla and mandible evidence a quite short skull in comparison with most non-ankylosaurid thyreophorans (Galton & Upchurch, 2004; Norman *et al*., 2004a; Vickaryous *et al*., 2004). The acute predentary and the slender cranial bones suggest both a narrow snout and a light skull, thus being more similar to the skull of basal thyreophorans and ornithischians (Norman *et al*., 2004a, b). In addition, the angle formed with the articulation of the predentary and the mandible not only suggest a narrow snout, but also a skull posteriorly narrower than that of the ankylosaurs (Vickaryous *et al*., 2004).

**Premaxilla.** The right premaxilla preserves the mid-posterior oral margin without teeth or alveoli, suggesting an edentulous bone, as in most eurypodans (stegosaurs + ankylosaurs; Galton & Upchurch, 2004; Vickaryous *et al*., 2004). The dorsal edge of the lateral vertical wall delimits the external nares, as in *Lesothosaurus* (Porro *et al*., 2015), *Emausaurus* (Haubold, 1990), *Scelidosaurus* (Norman, 2020a) and *Huayangosaurus* (Sereno & Dong, 1992), and unlike many eurypodans, where the premaxilla flares laterally (Sereno & Dong, 1992; Vickaryous & Russel, 2003). Laterally, the premaxilla-maxilla contact is broken, precluding to note if a groove is present as in *Huayangosaurus* (Sereno & Dong, 1992). Both sides of the oral margin of the premaxilla bear rugosities and foramina. Contrary to most thyreophorans, where the anterior region of the oral margin curves ventrally (forming a sinuous profile, see Maidment *et al*., 2008), the premaxilla is here aligned horizontally with the maxilla as in other ornithischians such as *Lesothosaurus* (Sereno, 1991), *Jeholosaurus* (Barta and Norell, 2021) and *Thescelosaurus* (Boyd, 2014).

**Maxilla**. The right maxilla preserves the anteroventral and the posterior regions. The anteromedial process contacts the premaxilla and is not strongly developed unlike the large process in *Emausaurus* (Haubold, 1990). The maxillary oral edge bears a diastema of 2 to 3 teeth until the first alveolus, as occurs in several ornithischians (Han *et al*., 2018). An extensive anterior maxillary diastema is present in *Paranthodon* (Raven & Maidment, 2018) and *Stegosaurus* (Gilmore, 1914). Medial and parallel to the tooth row, special foramina for replacement teeth are present. Medially, the posterior region bears a large crested surface for the palatine, as in *Scelidosaurus* (Norman, 2020a). Both the maxilla and the palatine form a dorsal shelf, and the former includes a lateral longitudinal groove for the jugal contact as in *Scelidosaurus* (Norman, 2020a). Anterior to this groove, the edge of the maxilla elevates dorsally, as occurs in *Kunbarrasaurus* (Leahey *et al*., 2015). There is no evidence of maxillary contribution to an antorbital fossa or fenestra, similar to the condition in ankylosaurs, where the antorbital fossa and fenestra are absent (Vickaryous *et al*., 2004). Laterally, a slight maxillary emargination is present and delimited by a rugose ridge, as in most ornithischians (Butler *et al*., 2008). Some nutrient foramina and small protuberances are present close to the jugal contact. The posterior end of the tooth row is subtly curved laterally. The teeth are wrapped by alveolar bone that extends slightly beyond the oral margin.

**Basisphenoid**. The block-like basisphenoid forms the floor of the braincase. It is not fused to the basioccipital or the pterygoid. The dorsal texture is rugose and pierced centrally by a groove. Anteriorly, the large pituitary fossa opens and laterally are the exits of the cranial nerve VI, as in *Scelidosaurus* (Norman, 2020a). Internal to the pituitary fossa, one foramen opens in the floor, and the Vidian canals are opened and run anteroposteriorly. These canals have three lateral exits from the pituitary fossa. The largest posterior exit (Vidian canal) is posterodorsal to the basipterygoid processes. The middle exit (for c. n. VII, following Norman, 2020b) converges with that of the c. n. VI in a double foramen placed anterodorsal with respect to the basipterygoid processes. Further anteriorly, the third exit is at the base of the cultriform process. The preserved plate-like base of the cultriform process is strongly developed, as in *Lesothosaurus* (Porro *et al*., 2015), *Thescelosaurus* (Boyd, 2014) and probably *Scelidosaurus* (Norman, 2020a). The basipterygoid processes are lateroventrally projected, with a subtle posterior deviation, as in *Scelidosaurus* (Norman, 2020a), contrasting the anteriorly directed processes of eurypodans (Galton & Upchurch, 2004; Vickaryous *et al*., 2004). As is common in thyreophorans and unlike other ornithischians, the basipterygoid processes are not close to the midline (Sereno, 1991; Norman *et al*., 2011; Boyd, 2014; Han *et al*., 2016). The lateral compression between the basipterygoid processes and the basal tubera is not strong, as in some ankylosaurs (e.g., Maryańska, 1977; Kinneer *et al*., 2016), and lacks lateral processes like those in *Yinlong* (Han *et al*., 216). The ventral process is small and carries a foramen (the exit of that is in the floor of the pituitary fossa), as in *Kunbarrasaurus* (Leahey *et al*., 2015).

**Lower jaw overview.** The left lower jaw is almost complete with teeth in situ. The most striking feature is the height of the posterior half, twice the height of the anterior half, and higher than that of most thyreophorans. It is formed by a well-developed coronoid eminence and a large ventral crest at the dentary-angular contact. This crest is formed by ventral projections of the dentary, the angular and the splenial, being the latter hidden in lateral view. A similar dentary flange is present in *Liaoceratops* (Xu *et al*., 2002) and *Psittacosaurus* (You *et al*., 2008 and references therein), though in the latter the flange is anteriorly recurved and mostly formed by the dentary. The deep posterior half of the mandible resembles the general shape of the heterodontosaurid and basal ceratopsian mandibles (You and Dodson, 2004; Norman *et al*., 2011; Sereno, 2012). However, the mandible is higher itself in these groups, whereas in *Jakapil* is exaggerated by the presence of the ventral crest. Preserved bones suggest the absence of a mandibular fenestra, as in *Scelidosaurus* and ankylosaurs (Norman, 2020a).

**Predentary.** The predentary is the first known for a non-eurypodan thyreophoran, and resembles that of *Lesothosaurus* (Sereno, 1991; Porro *et al*., 2015). Its main body is pyramidal-shaped with a rounded apex (in contrast to the acute apex in neornithischians such as *Thescelosaurus* and *Archaeoceratops*; You and Dodson, 2003; Boyd, 2014), and two large and rounded lateral processes. The ventral process is large (unlike most eurypodans and heterodontosaurids; Galton & Upchurch, 2004; Vickaryous *et al*., 2004; Norman *et al*., 2011; Sereno, 2012) and abuts against the anterior edge of the dentaries with a loose articulation, suggesting some movement between the elements (see Norman, 2020a; 2020b). Basal ceratopsians usually bear a predentary with a robust ventral process and reduced lateral processes (see Lambert *et al*., 2002; You *et al*., 2007). The ventral process is single as in *Lesothosaurus*, *Agilisaurus* and eurypodans, unlike the bifurcated ventral process of many neornithischians (Han *et al*., 2018). Ventral to the lateral process, a groove communicates with the anterior dentary foramen as in *Lesothosaurus* (Sereno, 1991; Porro *et al*., 2015). The external surface is ornamented with grooves and crests, suggesting a keratinous coverage.

**Dentary**. The edges of the dentary are subparallel anteriorly but they diverge posteriorly. The anterior end is dorsally wedged as in *Scutellosaurus* (Colbert, 1981), and unlike both the truncated dentary of *Scelidosaurus* (Norman, 2020a), heterodontosaurids (Sereno, 2012) and many ceratopsians (Hailu and Dodson, 2004), and the mid-height wedged of *Lesothosaurus* (Porro *et al*., 2015) and neornithischians such as *Haya* (Barta and Norell, 2021), *Thescelosaurus* (Boyd, 2014) and *Archaeoceratops* (You and Dodson, 2003). The symphysis is slightly spout-shaped as in most ornithischians (Boyd, 2015). The dorsal process elevates forming the anterior edge of the coronoid eminence, though it is posteriorly broken. The ventral process arises lateroventrally and extends further ventrally to contribute to the ventral crest. Its lateral surface is slightly ornamented with rugosities, grooves and tubercles, as in ‘*Bienosaurus*’ (Raven *et al*., 2019). Anteriorly, the oral margin is subhorizontal in lateral view, unlike the sinuous line of thyreophorans (synapomorphy of Thyreophora; see Butler *et al*., 2008; also present in *Scutellosaurus*, Breeden *et al*., 2021). The tooth row is inset medially, and subtly curved laterally towards the last teeth like those in non-ankylosaurian thyreophorans (Barrett, 2001). The emargination shows some large nutritious foramina. There are 10 teeth positions, similar to the 13 teeth of ‘*Bienosaurus*’ (Raven *et al*., 2019), but considerably less than most thyreophorans (usually more than 20; see Carpenter, 2004; Galton & Upchurch, 2004; Norman, 2020a), suggesting a shorter muzzle. A similar tooth count is present in heterodontosaurids (Sereno, 2012) and some basal ceratopsians (e.g. *Archaeoceratops*; You and Dodson, 2003). The teeth are vertically oriented, unlike the dorsomedially inclined teeth of some stegosaurs (e.g., *Stegosaurus* NHMUK PV R36730, Raven *et al*., 2019). Tooth crowns are arranged *en echelon*. Tooth roots are wrapped by alveolar bone. Four alternate special foramina (2, 4, 7 and 9) bear replacement teeth. Unlike the continuous condition observed in *Lesothosaurus* (Porro *et al*., 2015), *Scelidosaurus* (Norman, 2019), *Gargoyleosaurus* (Han *et al*., 2018) and *Huayangosaurus* (Sereno & Dong, 1992), there is a gap of two teeth between the predentary contact and the first dentary alveolus (shared with *Camptosaurus*, *Pinacosaurus*, *Thescelosaurus* and *Yamacertatops*, among other ornithischians; see Han *et al*., 2018).

**Splenial**. The splenial is a thin medial bone that partially covers the dentary medially from the third tooth backward. The posterior half is stouter, continuing the ventral dentary edge, and bears a ventral lamina that contributes to the ventral crest. Dorsally it bears the anterior and ventral edges of the internal mandibular (Meckelian) fenestra. A minute lamina of bone between the splenial and the dentary may belong to the coronoid, otherwise it is lacking.

**Angular**. The posteroventral edge of the mandible is composed of the angular. Anteriorly, a finger-like process of the angular enters in a dentary recess in lateral view as in *Scelidosaurus* (Norman, 2020a) and unlike most ornithischians. Ventrally, it contributes to the ventral crest and is ornamented with subtle rugosities and foramina. The broken angular of *Jakapil* avoids assuming the presence of an attached osteoderm as in *Scelidosaurus* and ankylosaurs (Norman, 2020a).

**Surangular**. The anterior two thirds of the surangular forms a lamina that comprises the posterodorsal wall of the mandible and delimits laterally and posteriorly the large adductor fossa. The thicker posterior third of the surangular contacts the articular medially, and bears a subcircular articular surface that complements the glenoid fossa of the articular. This glenoid component of the surangular slightly faces dorsomedially. Lateral to the glenoid, and right over the surangular foramen, it bears a tubercle. The tubercle seems anteriorly continued by a subtly developed horizontal inflection of the anterior lamina, above which is an extensive striated surface for muscle insertion. Although a strong surangular ridge in the lateral surface is absent, this inflection is placed along the same position of the surangular ridge of basal thyreophorans (synapomorphy of Thyreophora; Boyd, 2015), probably associated with the reduction of this structure in Eurypoda (Butler *et al*., 2008). On the other hand, the surangular ridge of ceratopsians is dorsally placed, forming a shelf with the dorsal edge of the surangular (Han *et al*., 2018). The complete surangular and coronoid bones, and the partial dentary, suggest a well-developed coronoid eminence like that of most eurypodans (see Sereno & Dong, 1992; Ősi *et al*., 2016), although a high eminence/process as in neornithischians and heterodontosaurids is not developed (see Xu *et al*., 2002; Barrett *et al*., 2005; Sereno, 2012). The surangular contribution to the retroarticular process is short and tongue-shaped.

**Prearticular**. The prearticular is similar to that of *Lesothosaurus* (Porro *et al*., 2015) and *Scelidosaurus* (Norman, 2020a). Anteriorly, it delimits the posterior and dorsal edges of the internal mandibular (Meckelian) fenestra. The dorsal edge delimits the adductor fossa medially. Its retroarticular contribution is short.

**Articular.** The articular forms the glenoid fossa anteriorly. The glenoid is subrounded (almost as long as wide, unlike the medially expanded glenoid of derived ankylosaurs; Ősi *et al*., 2016), and posteriorly straight. It is concave medially and flattened laterally. In lateral view, the glenoid is roughly aligned with the tooth row, as in *Gargoyleosaurus* (Kilbourne and Carpenter, 2005), *Shamosaurus* (Tumanova, 1987) and *Yinlong* (Han *et al*., 2016). The short retroarticular process bears a dorsomedially directed process that resembles that of several theropods (see the section ‘A novel thyreophoran anatomy’ in the main text). No thyreophoran (nor ornithischian) bears such a process, instead a medial projection of the glenoid occurs in several eurypodans (Vickaryous *et al*., 2004), though it is not homologous to the process.

**Dentition.** Tooth roots are elongated, cylindrical and barely curved along their main axis. Tooth crowns are leaf-shaped, labiolingually compressed, subrhomboidal, with one apical denticle surrounded by marginal ones, and asymmetrical, unlike the symmetrical crown teeth of several ornithischians (see Sereno, 1991; Barrett et al., 2005; Butler and Zhao, 2009; Boyd, 2014). A slight constriction separates the crown from the long root. The crown is swollen labially at its base, and lacks both cingulum and ornamentations such as grooves, striae or ridges, unlike those of derived eurypodans (Galton & Upchurch, 2004; Vickaryous *et al*., 2004), heterodontosaurids (Sereno, 2012) and most neornithischians (Tanoue *et al*., 2009; Boyd, 2014; Mallon and Anderson, 2014). The mesial edge of the labial surface in maxillary and dentary tooth crown is prominent as in *Scelidosaurus*^9^, but ends distally in a denticle-like structure in *Jakapil*. A striking difference with most thyreophorans is that maxillary and dentary tooth crowns of *Jakapil* are quite different. Dentary teeth bear one apical, 7 mesial, and 5-6 distal denticles, as in *Scelidosaurus* (Norman, 2020a), *Huayangosaurus* (Sereno & Dong, 1992) and *Gastonia* (Kinneer *et al*., 2016). The apical-most denticles curve distally, more than in *Scelidosaurus* and some ankylosaurs. Also, the mesial denticle row is lingually recurved in a greater degree than in *Huayangosaurus*. A flat to concave mesial surface lies between the curved denticle row and the labial isolated denticle. The maxillary teeth show acute and asymmetric crowns bearing one apical, 7-8 mesial and 4 distal denticles, resembling those of non-ankylosaurid thyreophorans (Haubold, 1990; Sereno and Dong, 1992; Ősi et al., 2016; Salgado et al., 2017; Norman, 2019). In contrast to the dentary teeth, the apical denticle is vertical and the mesial denticle row is straight.

Large and high angled wear facets are present in almost half of the teeth found (for both maxillary and dentary teeth; also for isolated and in situ teeth). In situ teeth show large wearing starting from the anterior teeth (at least the second position in both maxilla and dentary), and extending at least until half the dentary tooth row (fifth position). Isolated crowns with small or without facets have not been identified as functional or replacement teeth. Larger wear on maxillary tooth crowns is placed lingually along the base of the mesial denticles. Larger wear on dentary tooth crowns is extensive and placed labially, mostly on the mesial half (in tooth 4 it reaches almost all the labial surface). In dentary teeth, the mesial prominent edge and the swollen of the base delimits the facets, forming a basin-like surface for chewing as in *Scelidosaurus* and some nodosaurids (Barrett, 2001; Ősi *et al*., 2016).

**Vertebrae and ribs.** Axial elements are similar to those of *Scelidosaurus* (Norman, 2020c). The cervical centrum is anteroventrally broken. The posterior articular surface is flattened and seems almost as wide as high. The high position of the parapophysis suggests it is probably the last cervical. A large foramen is placed just posteroventral to the parapophysis. It seems disarticulated from the neural arch. In a posterior cervical neural arch, pre and postzygapophyses are not fused in the midline and rise to 50-60° from the horizontal. In the same, the diapophyses rise to 40° from the horizontal.

Dorsal vertebra elements are disarticulated or partially fused (neurocentral suture visible). Dorsal centra are cylindrical, with subcircular articular surfaces, and biconcave. These lack a ventral keel. The centra are anteroposteriorly longer than wide and high, and increase in both lateral compression and length posteriorly along the series. Centra are long as in basal ornithischians (e.g., *Lesothosaurus* and *Laquintasaura*; Barrett *et al*., 2014; Baron *et al*., 2017), *Scutellosaurus* (Breeden *et al*., 2021), a juvenile specimen of *Scelidosaurus* (NHMUK PV R6704; Norman, 2020c), and some ankylosaurs (e.g., *Jinyunpelta* and *Polacanthus*; Zheng *et al*., 2018; Raven *et al*., 2020). . The neural arch is low and resembles that of *Scelidosaurus* (Norman, 2020c) and *Thescelosaurus* (Brown *et al*., 2011), although the neural canal is comparatively larger here. The neural canal is subcircular anteriorly, and higher posteriorly. It is subtly higher than half the centrum height, as in some eurypodans (Raven & Maidment, 2017). The diapophyses are laterodorsally directed at almost 40° from the horizontal, lower than in most eurypodans (Raven & Maidment, 2017). The postzygapophyses are medially fused in a slender (width of less than a half postzygapophyses length) and strongly elongated posteriorly structure (more than in some ankylosaurs; see Arbour & Currie 2013; Blows 2015).

Dorsal ribs are free and not bifurcated. The proximal half of the rib has a T-shaped cross section. Some ribs are distally expanded and ornamented, like the anterior dorsal ribs of *Scelidosaurus* (Norman, 2020c) and *Huayangosaurus* (Zhou, 1984; Maidment *et al*., 2006). The low curvature of the shaft suggests a wide torso, as occurs in *Emausaurus* (Haubold, 1990), *Scelidosaurus* (Norman, 2020c), and the ankylosaurs (Vickaryous *et al*., 2004). Two fragments show a posterior broken thickness along the mid-portion of the shaft, suggesting the presence of intercostal bones, as in *Scelidosaurus* (Norman, 2020c), *Huayangosaurus* (Zhou, 1984; Maidment *et al*., 2006), some ankylosaurids (Maryańska, 1977; Park *et al*., 2021) and some basal ornithopods (Brown et al., 2011).

Sacrocaudal vertebra elements are fused, with a visible suture between them, resembling the fourth sacral (caudosacral) vertebra of a juvenile specimen of *Scelidosaurus* (NHMUK PV R6704; Norman, 2020c). The posterior articular surface of the centrum is flat to subtly concave. The neural canal is large and subtriangular. The neural spine is very low and anteriorly directed. Postzygapophyses are well-developed and divergent from each other.

A mid-caudal centrum is narrow and equidimensional in lateral view, as in some eurypodans (Ostrom, 1970; Sereno & Dong, 1992; Ősi, 2005). Articular facets are concave and oval-shaped. Transverse processes are small and button-like. The neurocentral suture is not visible. Postzygapophyses are medially fused and do not extend beyond the centrum edge.

**Pectoral girdle.** The coracoid and the scapula are not fused together. The scapular blade is elongated and mostly parallel-sided, without distal expansion (Supple. Fig. 2a, b), an overall shape that resembles that of several theropods (Gilmore, 1920; Burch, 2013), contrasting the distally expanded condition in most ornithischians (Baron *et al*., 2017; Han *et al*., 2018; Barta and Norell, 2021). A straight and parallel sided scapular blade is common in ankylosaurids (Maryańska, 1977; Arbour and Currie, 2013; Zheng *et al*., 2018). The proximal scapular plate resembles that of stegosaurs (Maidment *et al*. 2006a). The stegosaurian-like acromial process is high, and the lateral acromial crest resembles those of *Huayangosaurus* (Zhou, 1984). On the medial side, a large proximodistal groove seems to be the scapular continuation of the coracoid foramen; this groove is placed higher than in *Lesothosaurus* (Barrett *et al*., 2016) and *Scelidosaurus* (Norman, 2020c). The glenoid fossa is suboval and anteroventrally directed. Posterior to the glenoid fossa and over the ventral edge of the scapular blade, a low distinct ridge (Supple. Fig. 2a, b; r) represents the insertion site for the muscle *triceps longus caudalis*, as occur in ankylosaurids (Tumanova, 2000; Carpenter, 2004; Lü et al., 2007; Arbour and Currie, 2013; Zheng et al., 2018). This ridge is absent (or very reduced) in non-ankylosaurid ornithischians (Maidment *et al*., 2006; Baron *et al*., 2017; Morschhauser *et al*., 2018; Barta and Norell, 2021).

Though broken, the coracoid is much shorter than the scapula, unlike that of ankylosaurs, that bear a large coracoid (see Blows, 2015; Vickaryous *et al*., 2004). It bears a D-shaped, lateroventrally oriented (~45° from the coracoid plane) glenoid fossa. The coracoid and scapula contributions to the glenoid fossa are similar in size, is common in thyreophorans (see Arbour *et al*., 2016). The coracoid foramen is not preserved in the specimen.


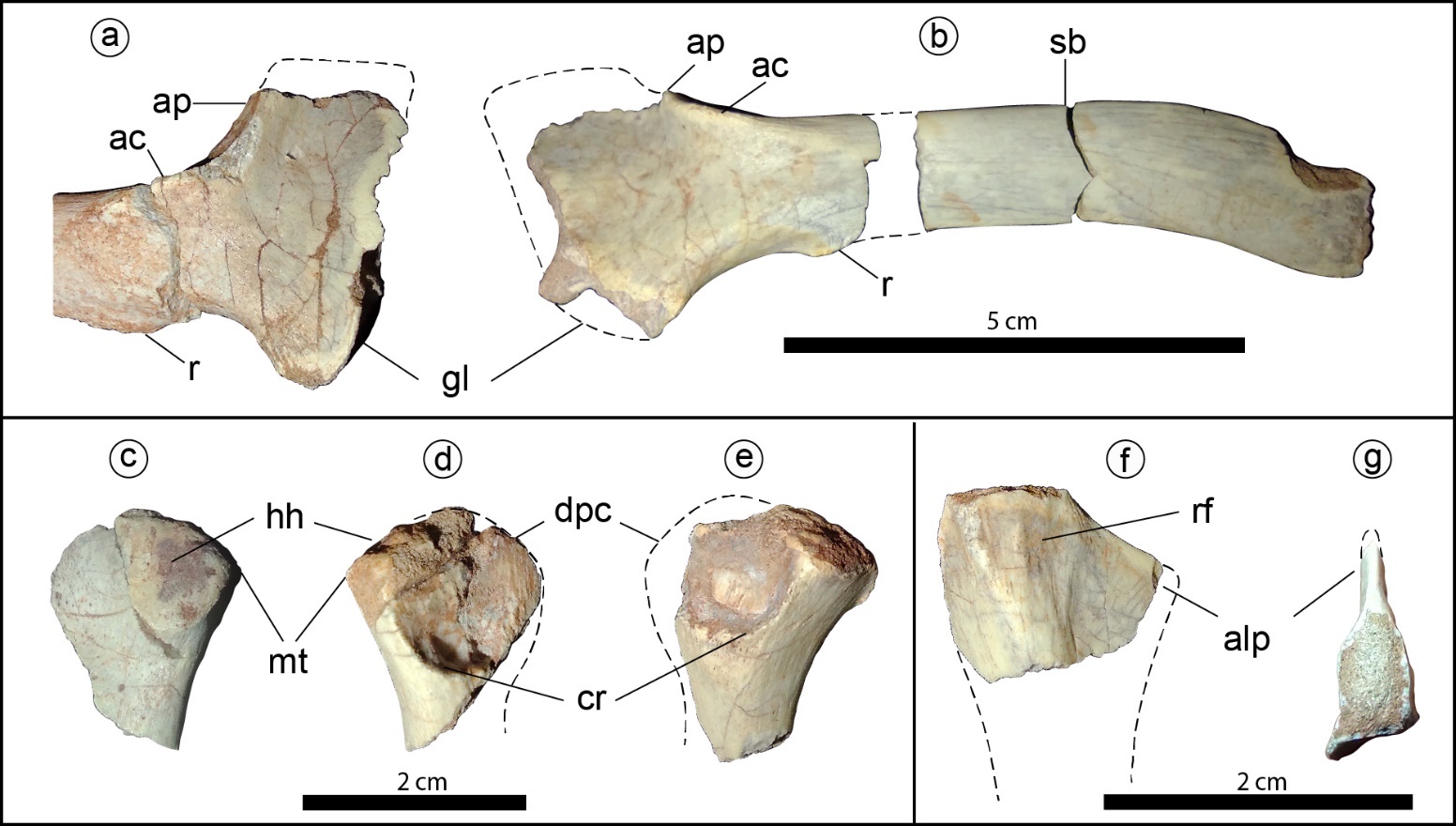


**Supplementary Figure 2.** Holotype of *Jakapil kaniukura* (MPCA-PV-630), pectoral girdle and limb bones. Right (**a**) and left (**b**) scapulae in lateral view; left proximal humerus in posterior (**c**) and anterior view (**d**), and right proximal humerus in anterior view (**e**); proximal ulna in lateral (**f**) and proximal (**g**) views. Dashed contours speculative. Abbreviations: ac, acromial crest; alp, anterolateral process; ap, acromial process; dpc, deltopectoral crest; gl, glenoid; r, ridge; sb, scapular blade.

**Forelimb elements.** The humeri (Supple. Fig. 2c-e) are strongly reduced in size, with overall limb proportions resembling those of basal ornithischians (Butler, 2010) and several theropods (Middleton & Gatesy, 2000; see Suppl. Figs. 3, 4 and the section ‘Bipedalism in armoured dinosaurs’ in the main text). The humeral head is large (as in eurypodans), medially placed and continuous with the medial tuberosity. The humeral head shows two continuous flattened articular surfaces (proximal and posterior), rather than being hemispherical. Anteriorly, the humerus bears a deep fossa (that is abraded) delimited by a curved ridge and the deltopectoral crest, as in *Lesothosaurus* (Baron *et al*., 2017; a shallow concavity is present in other basal ornithischians like *Agilisaurus* and *Hypsilophodon*; Peng, 1992; Galton, 1974). The proximal portion of the deltopectoral crest is rounded in anterior view, laterally directed and subtly rugose distally. The incompleteness of the bone avoids testing the presence of the descendent ridge and the triceps tubercle (synapomorphy of Stegosauria, Maidment *et al*., 2020).

A possible proximal end of the ulna (Supple. Fig. 2f, g) resembles that of basal ornithischians and thyreophorans, though it is much smaller and strongly laterally compressed. The proximal articular surface is broken, though the olecranon process seems poorly developed, as in *Scutellosaurus* (Breeden *et al*., 2021) and *Scelidosaurus* (Norman, 2020c). The anterolateral process is present as in *Scelidosaurus* (Norman, 2020c), eurypodans (Upchurch and Galton, 2004; Vickaryous *et al*., 2004), iguanodontians and ceratopsids (Maidment & Barrett, 2014).

The metacarpal II is stout and dumbbell-shaped, and resembles that of *Scelidosaurus* (Norman, 2020c). Although badly preserved, the proximal articular surface is flat to slightly convex. Distal condyles are separated by a shallow groove and collateral ligament pits are poorly developed. The proximal end of another metacarpal (I or III) shows a convex surface.

**Pelvic girdle.** Both left and right ischial elements are known (Supple. Fig. 3c). The right ischium is poorly preserved. The iliac peduncle presents bores (probably due to bioerosion) on the articular surface, and is separated from the pubic articulation, unlike the continuous and cup-shaped structure of most ankylosaurs (Vickaryous *et al*., 2004; Carpenter *et al*., 2013). The partial ischial shaft is straight, laterally compressed and parallel edged, like those of *Scutellosaurus* (Breeden *et al*., 2021) and *Scelidosaurus* (Norman, 2020c). Distally, it tapers as in stegosaurs (Maidment *et al*., 2015).


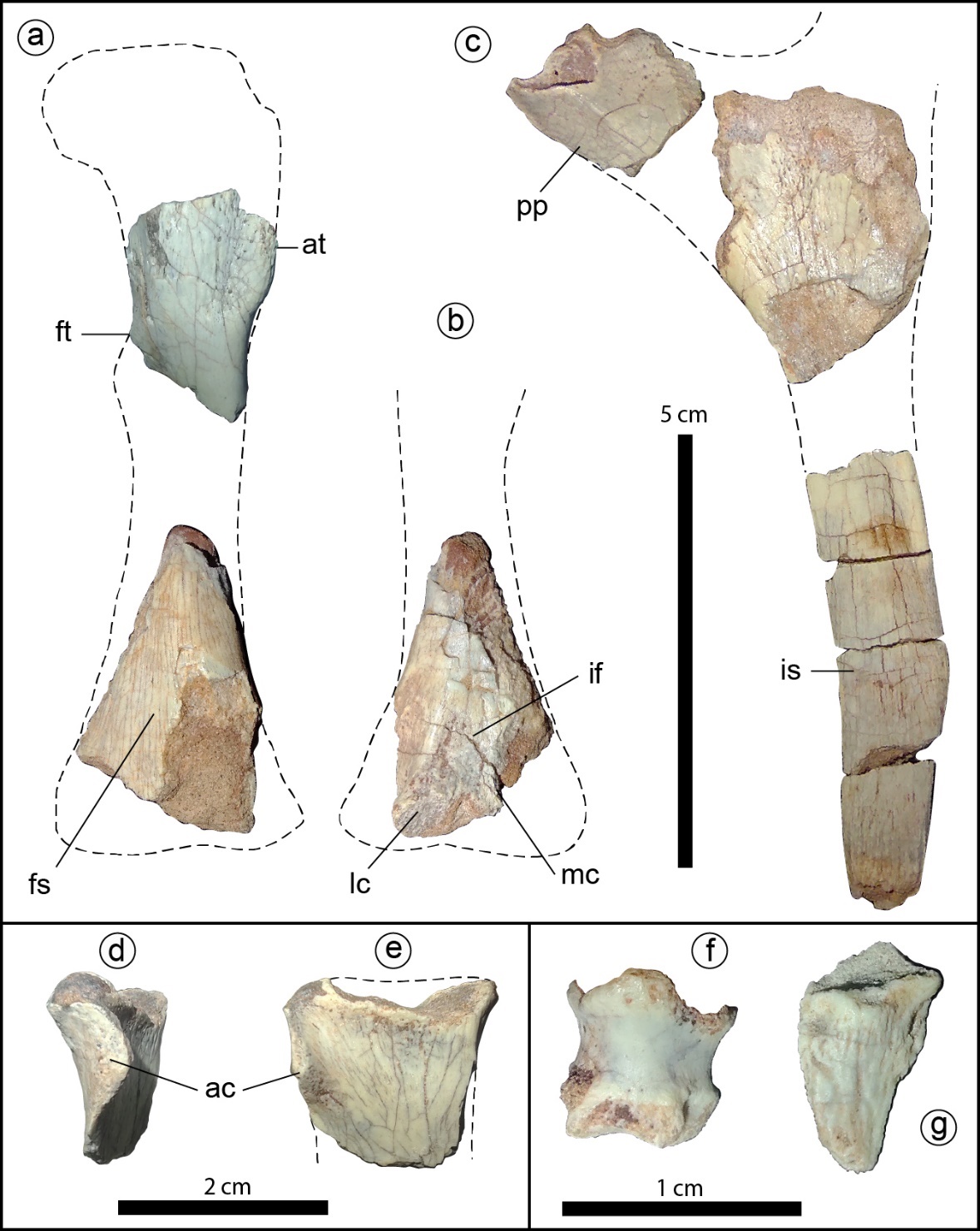


**Supplementary Figure 3.** Holotype of *Jakapil kaniukura* (MPCA-PV-630), pelvic girdle and limb bones. Femoral fragments in anterior (**a**) and posterior (**b**) views, and ischial fragments in side view (**c**) (dashed contours based on *Scelidosaurus*^9^); proximal right fibula in anterior (**d**) and left lateral (**e**) views; non-ungual phalanx in dorsal view (**f**) and ungual phalanx in ventral view (**g**). Abbreviations: ac, anterior crest; at, anterior trochanter; if, intercondylar fossa; is, ischial shaft; lc, lateral condyle; mc, medial condyle; fs, flattened surface; ft, fourth trochanter; pp, pubic peduncle.

**Hindlimb elements.** The partial femur (Supple. Fig. 3a, b) preserves the bases of both the anterior and the fourth trochanters, and resemble those of basal ornithischians (e.g., *Lesothosaurus*, *Heterodontosaurus* and the juvenile specimen of *Scelidosaurus*; Sereno, 2012; Baron *et al*., 2017; Norman, 2020c). The anterior trochanter is separated from the femoral shaft by a shallow groove and shows marginal rugosities (anterolateral ridge in *Scelidosaurus*, Norman 2020c). The base of the fourth trochanter is placed posteromedially, almost at the same level of the base of the anterior trochanter, thus both trochanters are closer each other than those of *Scelidosaurus*. The bases of both trochanters are large, suggesting large elements, though it is not possible to know their full extension. The distal end is expanded and subtly curved posteriorly. The posterior intercondylar groove is shallow. Though the distal condyles are mostly eroded, the medial condyle seems larger than the lateral one.


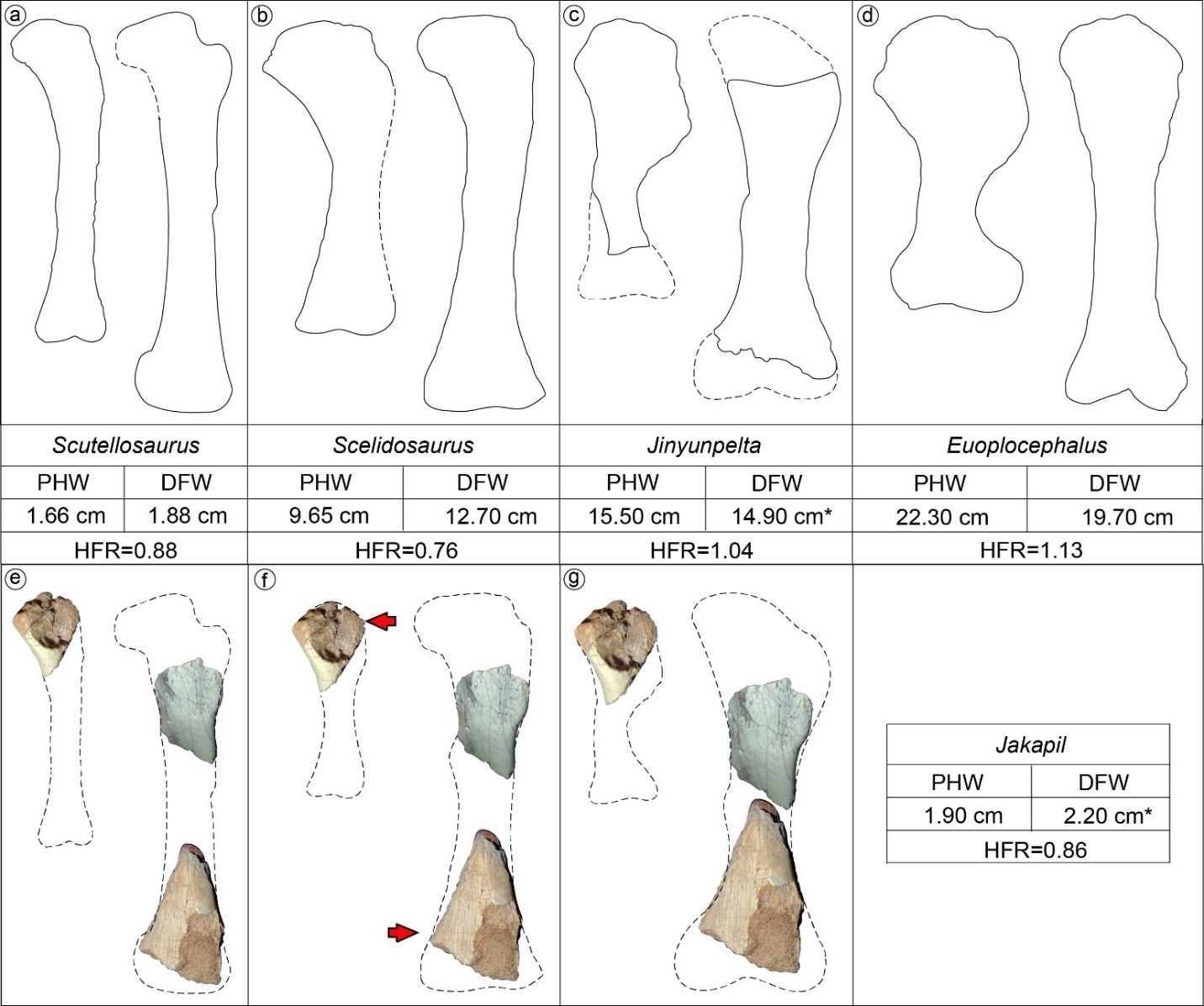


**Supplementary Figure 4**. Limb comparison between thyreophorans*.* *Scutellosaurus* (MNA. V. 175, **a**), *Scelidosaurus* (NHMUK R1111, **b**), *Jinyunpelta* (ZMNH M8960, **c**), *Euoplocephalus* (AMNH 5404, **d**); qualitative estimation of *Jakapil* limbs based in *Scutellosaurus* (e), *Scelidosaurus* (f) and *Jinyunpelta* (g). Abbreviations: PHW, proximal humerus width; DFW, distal femur width; HFR, proximal humerus/distal femur width ratio. Silhouettes were modified from Arbour & Currie (2013, Zheng *et al*. (2018), Norman (2020c), and Breeden *et al*. (2021). Dashed lines represent missing parts. Arrows indicate the site of width measurement in *Jakapil*.

The femur is incomplete, but a preliminary femoral-based body mass estimation was possible. We used the non-phylogenetic scaling equation for biped dinosaurs of Benson *et al*. (2018), and incorporated the distal circumference of the proximal femoral fragment with two methodologies. Measuring the real circumference (45 mm), a body mass of 7.15 kg was estimated for *Jakapil*. Measuring the circumference of the oval formed with both minimum anteroposterior and lateromedial diameters (38 mm), a body mass of 4.5 kg was estimated. Both masses are comparable with the 6.31 kg estimated for *Lesothosaurus* (Benson *et al*., 2018).

The distal end of the right tibia is distally expanded and the articular surface is eroded. The lateral expansion is anteroposteriorly compressed and bears a subtle oval surface to contact the fibula.

The proximal end of the right fibula is laterally compressed and seems proportionally larger than that of all other thyreophorans (as compared with both the femur and the tibia). Its articular surface is mostly broken. The medially curved anterior crest (Suppl. Fig. 3d, e; ac) is large and proximally displaced when compared with that of *Scelidosaurus* (Norman, 2020c) and *Kentrosaurus* (Galton, 1982b). A similar process (though not a crest) is present in some hadrosaurids (e.g., McDonald et al., 2012; Cruzado Caballero and Powell, 2017).

Non-ungual phalanges II and III of the left pedal digit IV are block-like, equidimensional in dorsal view, stouter proximally and expanded at the articular surfaces (Supple. Fig. 3f, 1), similar to those of non-derived ornithischians (e.g., Brown *et al*., 2011; Baron *et al*., 2017; Morschhauser *et al*., 2018; Norman, 2020c), and different from the wider (usually disk-shaped) phalanges of derived groups (Galton, 1982b; Mallon & Holmes, 2010; Sissons, 2011; Zheng et al., 2011). Distal condyles are well developed, but collateral ligament pits are not. The ungual phalanx of the digit IV (Supple. Fig. 3g) is very similar to that of *Scelidosaurus*. It is dorsoventrally flattened, bluntly pointed and slightly bent medially towards the tip. It is ornamented with pits and furrows, rougher in the dorsal surface. Ventrally, a notch separates the proximal oval surface from the collateral edges and claw grooves distally, as in *Scelidosaurus* and unlike other ornithischians (Norman, 2020c), where these are visible in dorsal view (e.g., Baron *et al*., 2017; Morschhauser *et al*., 2018).

**Osteoderms.** Osteoderms are represented by at least five different morphological types. All osteoderms are finely rough externally and full of vascular pits and small furrows, increasing in density towards the edges and over their crest. Since the skeleton was disarticulated, the classification into cervical and postcervical osteoderms was performed on the basis of comparisons with other thyreophorans.

Two cervical elements are the largest osteoderms preserved for *Jakapil*, and are composed of an external osteoderm over a fused smooth bone base, as in *Scelidosaurus* (Norman, 2020d) and several ankylosaurs (Ford, 2000; Arbour & Currie, 2016). However, the osteoderms in *Jakapil* are quite low, much flatter than in other thyreophorans. These are subhexagonal, strongly depressed, and show a subtle central crest. The base is smooth, slightly concave and shows large foramina, as in ankylosaurs, rather than the rugose bases of *Scelidosaurus*. The lateral edge of the base bears projected bone sheets, suggesting a strong suture with another dermal element.

A probable cervical element is also composed by a concave base of smooth bone and an osteoderm. However, the dermal bone is high, asymmetric and apically compressed. The base also presents strong rugosities at the edge, suggesting a sutural contact with the former pieces, as in *Scelidosaurus* (Norman, 2020d) and some ankylosaurs (Ford, 2000).

The placement of postcervical osteoderms on the body is unknown. Most of these elements are strongly flattened, disk-shaped, suboval and with a very low crest, resembling those of certain ankylosaurs (see Ford, 2000; Kilbourne & Carpenter, 2005; Kinneer *et al*., 2016, ‘body osteoderms’). The dorsal crest may be central or eccentric, and straight or flexed in external view. The edges are slightly elevated, allowing shallow internal circumferential depressions. A few osteoderms bear a slightly higher crest with a variable concave base, thickened edges and similar circumferential depressions. One osteoderm shows a high triangular cross section, like those of *Scelidosaurus* (Main *et al*., 2005; Norman, 2020d).

Conical spike-like osteoderms with deep concave bases are also known in *Jakapil*. The basal edges are crenulated and the external furrows are larger than those of other osteoderms. Ventrally, they are smooth with some large foramina.

Finally, many flat, disk-shaped, minute (7-10 mm) ossicles are present. These are subcircular and lack an external crest.

**Histology and ontogenetic state**

**Description.** The proximal and the mid-distal regions of two different dorsal ribs and five osteoderms were sectioned following standard paleohistological techniques (Cerda et al., 2020; Suppl. Fig. 5). The gross histology is similar of that of other thyreophorans (Padian & Woodward, 2021). The thin section of the proximal half of the dorsal rib is T-shaped, bearing an anteroposteriorly expanded lateral side (Suppl. Fig. 5b, top). It shows a compact external cortex and a core of coarse cancellous bone. The lateral cortex is thinner than the medial one. The thicker medial cortex allows a better view of its histological features (Suppl. Fig. 5b, bottom). The primary tissue is lamellar-zonal, with annuli of pseudolamellar tissue and vascularized zones with fibrolamellar matrix around the coarse cancellous bone. Secondary osteons are present between the cancellous bone and the cortex, and are abundant in the lateral side. The cortex is moderately vascularized with scattered and predominantly longitudinal primary osteons, which have a regular circumferential arrangement on the medial side. The abundance of canals is higher in the inner fibrolamellar cortex, and diminished towards the subperiosteal margin (although it does not disappear). Few canals are obliquely oriented, and very few open to the subperiosteal margin. At least seven lines of arrested growth (LAGs) associated to annuli have been identified in the proximal region of the shaft, and at least six in the mid-shaft (Suppl. Fig. 5b). These are approximately equally spaced, the last one being very close to the subperiosteal margin. Despite the very poor vascularization external to the outer most LAG, a distinct External Fundamental System (EFS) is not evident.

The osteoderms are composed by thin external and basal cortices that enclose a strongly cancellous core (Suppl. Fig. 5c top). External cortex is well vascularized with mostly oblique and radial canals. Basal cortex is poorly vascularized, though some large canals are present entering through the osteoderm base. Secondary osteons are present at the internal cortex limit in larger osteoderms. The external cortex is somewhat thicker than the internal one, although this may vary locally. Osteoderms are composed of structural collagen fibres bundles as in ankylosaurs and titanosaurs (Scheyer & Sander, 2004; Cerda & Powell, 2010; Burns & Currie, 2014), ordered in sets parallel and oblique to the osteoderm surface (in some cases, the external cortex presents transversal structural fibre bundles). Some osteoderms present between one and two poorly defined growth marks, which are visible in the external cortex (Suppl. Fig. 5c, bottom).


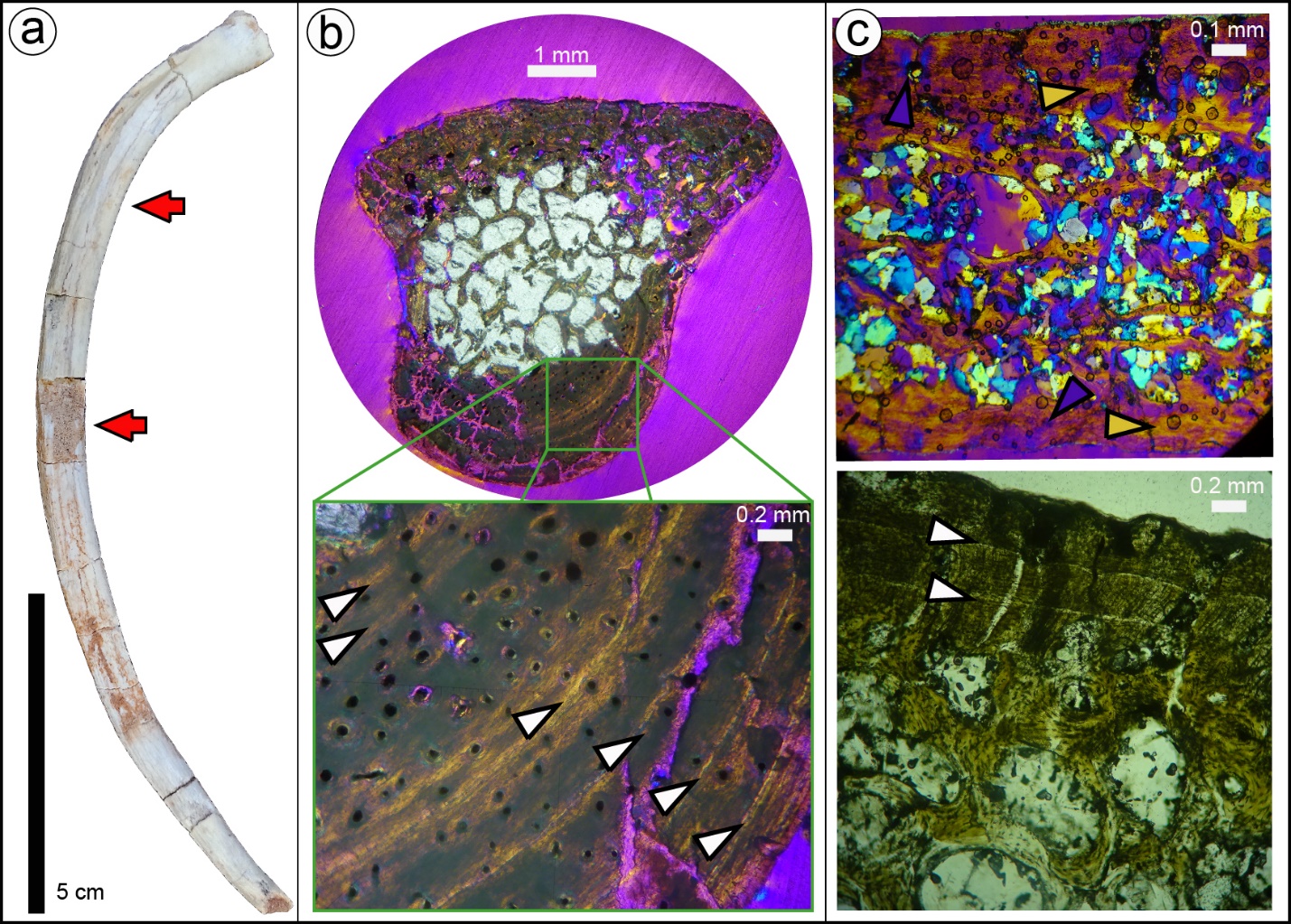


**Supplementary Figure 5**: histology of *Jakapil*. **a**, dorsal rib (red arrows indicate the two sectioned regions); **b**, thin section of the dorsal rib mid-shaft, general view (top) and detail of the medial region (bottom); **c**, thin sections of two osteoderms, showing the bundles of structural fibres of the external and basal cortices with polarized light plus a lambda filter (top), and the detail of an external cortex under normal light (bottom). White arrows indicate LAGs (**b**, **c** bottom). Blue and yellowish arrows point direction of some bundles of structural fibers (**c**, top).

**Discussion.** Regarding the ontogenetic state of *Jakapil*, rib tissues provide some insights. As a whole, the presence of pseudolamellar tissue around the fibrolamellar bone (increasing in fibre organization towards the subperiosteal margin), the presence of secondary remodelling (even abundant in some sectors), the majority of longitudinal primary canals with reduced diameters, and a moderate to low vascularity externally, show a decrease in the growth rate and the absence of typically juvenile histological features in the *Jakapil* holotype (Padian *et al*., 2004; Chinsamy-Turan, 2011; Padian & Woodward, 2021; and references therein). Despite the increasing fibre arrangement in the middle and outer cortices, the regular growth mark spacing (non-decreasing) towards the bone surface could suggest sexual immaturity of the holotype of *Jakapil* (Erickson *et al*., 2007).

The partial long bones of *Jakapil* preclude a correct sampling for a skeletochronology study. However, ribs have been used to study thyreophoran histology with similar results to those obtained with long bones. In *Antarctopelta* (Cerda *et al*., 2019) and *Stegosaurus* (Hayashi *et al*., 2009), LAG count in limb bones are similar to those observed in dorsal ribs. In ankylosaurs, extensive remodelling obliterates growth marks even in ribs (Stein *et al*., 2013). The absence of an EFS and the presence of seven growth marks in *Jakapil*, as compared with the large number of LAGs present in adult individuals of other thyreophorans (8-13 in *Antarctopelta* and *Stegosaurus*; Hayashi *et al*., 2009; Cerda *et al*., 2019), suggest that the holotype of *Jakapil* does not belong to a fully grown individual. In addition, the absence of an EFS in the holotype individual of *Jakapil* suggests that the somatic maturity was not reached (Padian *et al*., 2004; Chinsamy-Turan, 2011). Therefore, although the holotype individual may have reached almost 1.5 m, it is not yet fully grown.

Following the ontogenetic scale of Hayashi *et al*. (2009) for *Stegosaurus*, the absence of an EFS and the presence of growth marks suggest that the holotype individual of *Jakapil* may correspond to a young adult (stage 3). However, the remodelling in *Jakapil* is low and concordant with a subadult *Stegosaurus* individual (stage 2), and also with the adult *Scutellosaurus* (Padian *et al*., 2004). In both adult *Stegosaurus* and ankylosaurs, the degree of remodelling is higher, extensively filling the compact cortex (Hayashi *et al*., 2009; Stein *et al*. 2013; Cerda *et al*., 2019). Moreover, Hayashi *et al*. (2009) have noted that the young adult *Stegosaurus* (stage 3) externally shows some features traditionally considered as juvenile proxies, mainly related to the lack of fusion of several structures. The same occurs in basal thyreophorans, that bear unfused or partially fused presacral vertebrae and girdle elements, not necessarily suggesting a juvenile or immature individual (Breeden & Rowe, 2020; Norman, 2020c). This condition is also present in *Jakapil*.

In addition to the lack of fusion in some bones (e.g. basisphenoid with basioccipital, and coracoid with scapula), and the mostly broken articular surfaces (resembling certain bioerosion patterns in aeolian environments rather than unossified bone; see Saneyoshi *et al*., 2011), the high number of denticles on the tooth crowns, the high acromion process, the anterior ridge over both the anterior trochanter and the proximal end of the fibula support a non-juvenile specimen (see Galton, 1982a,b; Burns *et al*., 2011; Norman, 2020c). *Jakapil* also lacks fusion between parasagittal cervical armour elements, although the osteoderms are fully fused to their respective bases, as occurs in a large, not fully grown *Scelidosaurus* (BRSMG LEGL 0004; Norman, 2020d).

The presence of postcervical osteoderms also supports a non-juvenile state for the *Jakapil* holotype. Several authors have noted that postcervical osteoderms are absent in juvenile ankylosaurs (e. g. Maryańska, 1977; Galton, 1982a; 1982b; Coombs, 1986; Burns *et al*., 2011; Norman, 2020c). Furthermore, the presence of fewer growth marks in the compacta of the osteoderms than in the dorsal ribs supports that these elements appear later in the ontogeny. However, the unknown relative timing and body pattern of osteoderm development precludes an approximation to known a possible age of appearance of these structures in the animal body. A similar pattern is present between dermal plates and endochondral bones in *Antarctopelta* (Cerda *et al*., 2019). Conversely, the dermal ossicles of *Antarctopelta* show a large amount of growth marks, suggesting that this kind of osteoderms appears early in the ontogeny of the genus. Similar ossicles are also present in other ankylosaurs (e. g. *Kunbarrasaurus*; Molnar, 2001; Leahey *et al*., 2015), but are apparently absent in *Jakapil*. Anyway, histological evidence supports that postcervical osteoderms are absent at earlier ontogenetic stages in *Jakapil*. In addition, the relative cortical thickness in the osteoderms of *Jakapil* resembles that of the ‘polacanthids’ and some ankylosaurids (Burns & Currie, 2014).

As a whole, the histology of *Jakapil* is intermediate between that of *Scutellosaurus* (Padian *et al*., 2004) and eurypodans (Padian & Woodward, 2021) in some aspects. In *Scutellosaurus*, the predominance of parallel fibered bone represents a slow growing rate, proposed by Padian *et al*. (2004) as typical for small dinosaurs. In *Jakapil*, however, the presence of zonal bone with fibrolamellar matrix represents periods of high growth rate. Also, both taxa have a low proportion of secondary tissue in the innermost cortex. These features contrast with the condition observed in stegosaurs and ankylosaurs (even in juveniles), which show both abundant fibrolamellar bone (suggesting a higher growing rate) and extensive remodelling (Hayashi *et al*. 2009; Stein *et al*., 2013; Padian & Woodward, 2021, and references therein). Sampled material of both *Jakapil* and *Scutellosaurus* represent very small (less than 2 m in length) non-juvenile individuals.

# Testing the phylogenetic placement of *Jakapil kaniukura*

**Data matrix of Soto-Acuña *et al*. (2021)**

In the main text we include the phylogenetic results recovered with the matrix of Soto-Acuña *et al*. (2021). The dataset consists of 75 taxa and 383 morphological characters and is attached at the end of this archive (see character list in Han *et al*., 2018 and Soto-Acuña *et al*., 2021). *Marasuchus* was fixed as the outgroup-most taxon. All characters were unweighted. Characters 2, 23, 31, 39, 125, 163, 196, 203, 204, 222, 227, 238, 243, 247, 268, 292, 296, 302, 306, 320 and 361 were treated as additive. Character 269 (scapula-coracoid fusion, absent/present), which is probably under ontogenetic control (see Burns, 2015; Maidment *et al*., 2015), was not codified due to the subadult ontogenetic state of the holotype of *Jakapil*. Memory space was made for 1,500,000 trees. A Traditional search was carried out in TNT v1.5 (Goloboff & Catalano, 2016), with 10,000 replicates of Wagner trees under the tree bisection reconnection (TBR) algorithm, saving 10 trees per replication. Trees saved in memory were resampled with an additional round of TBR. Bremer values were also recorded with Traditional searches until 22 suboptimal trees. Bootstrap analysis was carried out using 10,000 pseudoreplicates with a Traditional search, and Absolute frequencies. We recovered *Jakapil* within Thyreophora, as the sister taxon of Eurypoda (Suppl. Fig. 6).

This analysis allows us to test *Jakapil* as a thyreophoran. Also, we include below other analyses with some other datasets to test the phylogenetic position of *Jakapil* within Thyreophora (Norman, 2020b, Wiersma & Irmis, 2018 and Maidment *et al*., 2020).


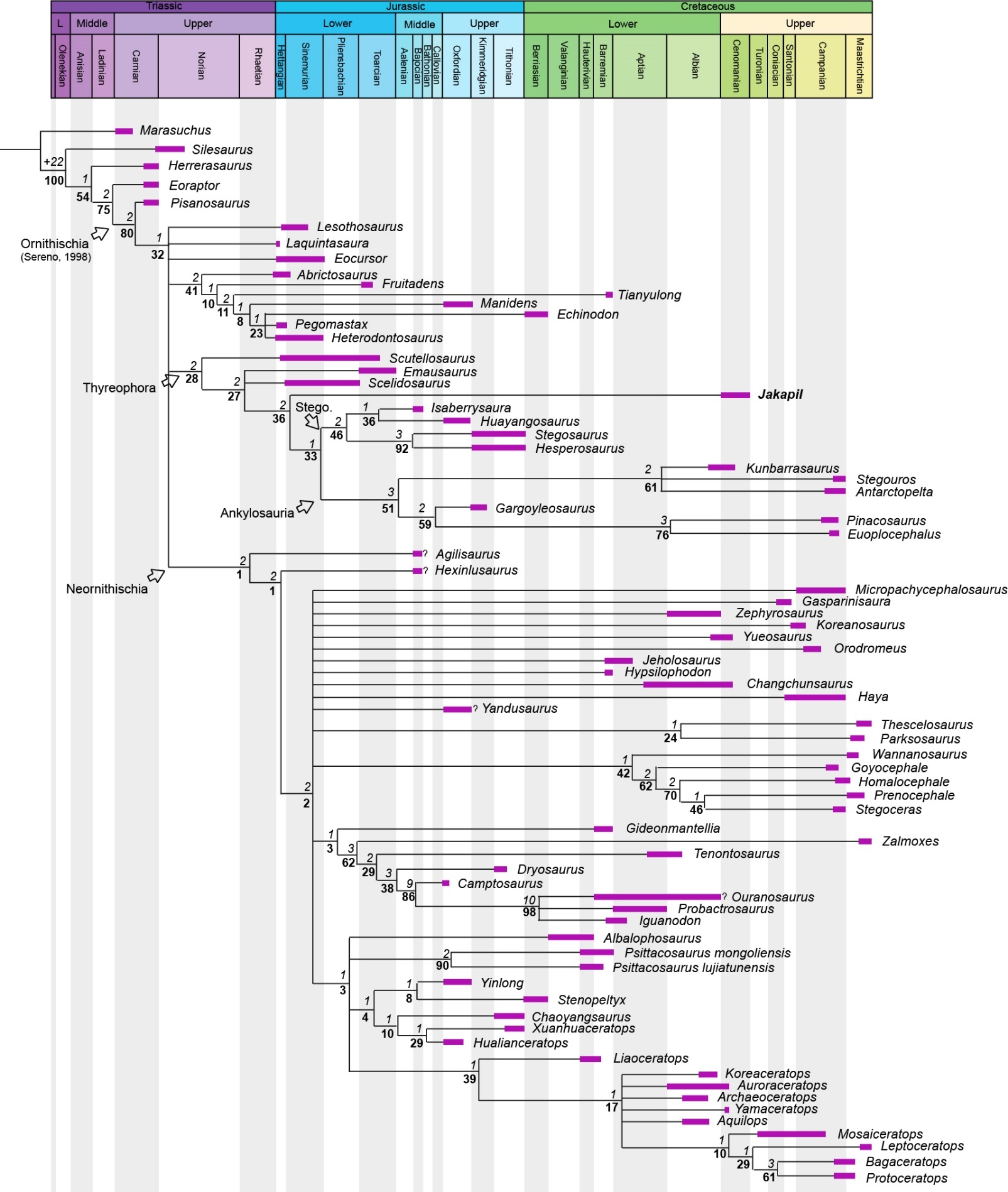


**Supplementary Figure 6**: Time calibrated strict consensus of 26,784 most parsimonious trees (L=1268). CI: 0.359, RI: 0.708. Branch supports are figured (Bremer /Bootstrap). Ages of each record were taken from Norman (2002), Barrett *et al*. (2005), Butler & Zhao (2009), Ohashi & Barrett (2009), Pol *et al*. (2011), Ruiz Omeñaca *et al*. (2012), Sereno (2012), Ősi *et al*. (2016), Bertozzo *et al*. (2017), Maiorino *et al*. (2017), Dieudonné *et al*. (2020), Maidment *et al*. (2020), Müller & Silva García (2020), Soto-Acuña *et al*. (2021).

**Synapomorphies of Thyreophora present in *Jakapil*:**

Char. 259: 0🡪1: Postcranial osteoderms: present.

**Autapomorphies of *Jakapil*:**

Char. 134: 0🡪1: Basipterygoid processes, orientation: ventral (shared with *Agilisaurus*, *Hypsilophodon*, *Zalmoxes*, *Tenontosaurus*, *Dryosaurus*, *Liaoceratops*, *Yamaceratops*, *Leptoceratops*, *Bagaceratops* and *Protoceratops*).

Char. 136: 0🡪1: Basipterygoid process articular facet orientation: laterally (shared with *Homalocephale*, *Prenocephale*, *Stegoceras* and *Yinlong*).

Char. 166: 1🡪0: Dentary tooth row (and edentulous anterior portion) in lateral view: straight (shared with the ornithischians *Lesothosaurus*, *Eocursor*, *Scutellosaurus*, *Pinacosaurus*, *Euoplocephalus*, heterodontosaurids and neornithischians).

Char. 170: 0🡪1: Ventral flange on dentary: present (shared with *Psittacosaurus, Yamaceratops* and *Protoceratops*).

Char. 174: 0🡪1: Coronoid process: well-developed, distinctly elevated, depth of mandible at coronoid is more than 150% depth of mandible beneath tooth row (shared with heterodontosaurids and neornithischians).

Char. 183: 0🡪1: Surangular length: more than 50% (1) of mandibular length (shared with *Stegoceras*, *Psittacosaurus, Yinlong, Chaoyangsaurus* and *Hualianceratops*).

Char. 204: 1🡪0: Dentary teeth, number of alveoli: less than 15 (shared with heterodontosaurids, *Gasparinisaura*, *Hypsilophodon*, *Wannanosaurus*, *Tenontosaurus*, *Dryosaurus* and ceratopsians).

Char. 205: 1🡪0: Cheek teeth, crown shape: apicobasally tall and blade-like (shared with *Laquintasaura*, *Psittacosaurus, Yinlong, Chaoyangsaurus* and *Hualianceratops*).

Char. 256: 1🡪0: Epaxial ossified tendons present along vertebral column: absent.

**Data matrix of Norman (2020b)**

In the main text we include the phylogenetic results recovered with the newly elaborated matrix of Norman (2020b). The dataset consists of 19 taxa and 115 morphological characters and is attached at the end of this archive (see character list in Norman, 2020b). *Silesaurus* was fixed as the outgroup-most taxon. All characters were unweighted and non-additive. Character 35 (basisphenoid-basipterygoid fusion, absent/present), which is probably under ontogenetic control (see Norman, 2020a), was not codified due to the subadult ontogenetic state of the holotype of *Jakapil*. Memory space was made for 2,000,000 trees and most parsimonious trees (MPT) were searched with an exact search (implicit enumeration). The analysis was made with TNT v1.5 (Goloboff & Catalano, 2016). We recovered *Jakapil* within the Ankylosauromorpha, as the sister taxon of the Ankylosauria (sensu Norman, 2020b; Suppl. Fig. 7).

Since Norman (2020b) used other software to make the phylogenetic analyses, we tested the branch support previous to the inclusion of *Jakapil*, and these were similar to those of Norman. The inclusion of *Jakapil* considerably diminished the branch support for the non-Ankylosauria ankylosauromorphs compared with those obtained by Norman (2020b), as well as the Bremer support for both Ankylosauria (sensu Norman, 2020b) and Stegosauria. However, *Jakapil* is well supported within Thyreophora.


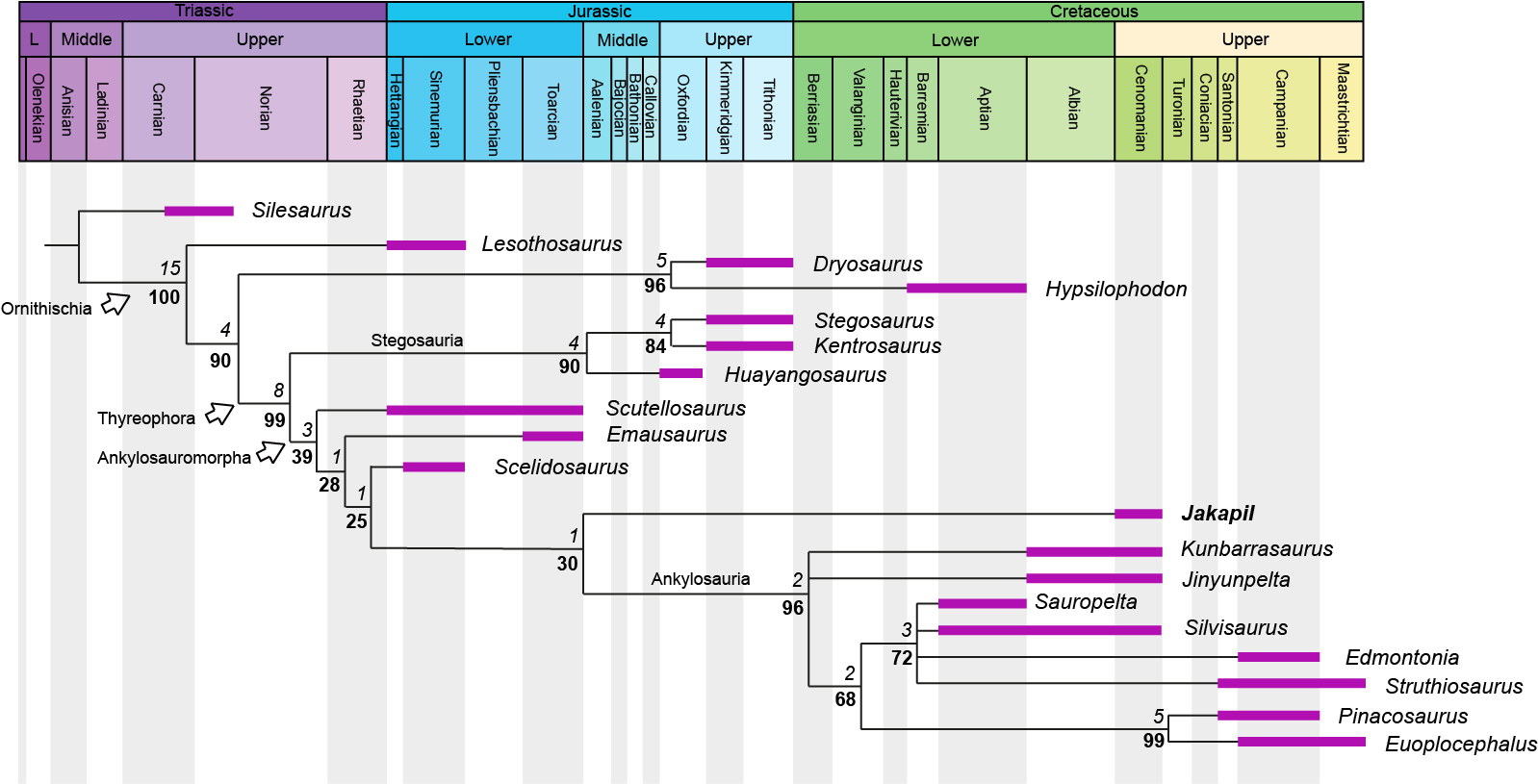


**Supplementary Figure 7**: Time calibrated strict consensus of 6 most parsimonious trees (L=232). CI: 0.702, RI: 0.867. Branch supports are figured (Bremer /Bootstrap). Ages of each record were taken from Ősi & Prondvai (2013), Boyd (2015), Norman (2020b), Maidment *et al*. (2020) and Breeden *et al*. (2021).

**Synapomorphies of Thyreophora present in *Jakapil*:**

Char. 29: 0🡪1: Mandibular condyles of the quadrate: medial larger than lateral condyle.

Char. 42: 0🡪1: Dorsal margin of dentary in occlusal view: mildly bowed medially.

Char. 95: 0🡪1: Fourth trochanter position: midlength.

Char. 96: 0🡪1: Transverse width of distal femur: >150% depth of medial condyle.

Char. 104: 0🡪1: Osteoderms form parasagittal rows either side of dorsal midline: present.

**Synapomorphies of Ankylosauromorpha present in *Jakapil*:**

Char. 105: 0🡪1: Osteoderms form multiple parasagittal rows over the torso: present.

**Autapomorphies of *Jakapil*:**

Char. 41: 1🡪0: Dentary dorsal margin in lateral view: straight (shared with *Silesaurus*, *Lesothosaurus*, *Dryosaurus*, *Hypsilophodon*, *Huayangosaurus*, *Kentrosaurus*, *Stegosaurus* and *Scutellosaurus*).

Char. 65: 0🡪1: Humerus: femur ratio: <80% (shared with *Lesothosaurus*, *Dryosaurus*, *Hypsilophodon*, *Kentrosaurus*, *Stegosaurus*).

Char. 103: 1🡪0: Epaxial ossified tendons along vertebral column: absent (shared with *Silesaurus* and *Stegosaurus*).

**Data matrix of Wiersma & Irmis (2018)**

The database including *Jakapil* presents 36 taxa and 293 morphological characters and is attached at the end of this archive (see character list in Wiersma & Irmis, 2018). All characters were unweighted, and characters 1, 2, 3, 7, 10, 13, 16, 18, 23, 25, 30, 36, 38, 48, 49, 54, 64, 87, 98, 101, 103, 104, 105, 140, 141, 143, 145, 148, 149, 156, 162, 165, 174, 177, 194, 201, 205, 209, 217, 229, 231, 232, 236, 237, 238, 260, 268, and 279 were additive. Characters 74 (basisphenoid-basipterygoid fusion), 172 (coracoid-scapula fusion), and 228 (dermal armour pitting), which are probably under ontogenetic control (see Galton, 1982a; 1982b; Hayashi *et al*., 2009; Norman, 2020a), were not codified due to the subadult ontogenetic state of the holotype of *Jakapil*. Memory space was made for 2,000,000 trees. A Traditional search was carried out in TNT v1.5 (Goloboff & Catalano, 2016), with 10,000 replicates of Wagner trees under the tree bisection reconnection (TBR) algorithm, saving 10 trees per replication. Trees saved in memory were resampled with an additional round of TBR. Bremer values were also recorded with Traditional searches until 6 suboptimal trees. Bootstrap analysis was carried out using 10,000 pseudoreplicates with a Traditional search, and Absolute frequencies.

As in the analysis of Wiersma & Irmis (2018), we recovered Stegosauria and Ankylosauria as sister groups (the traditional Eurypoda), as well as the successive sister taxa *Emausaurus* and *Scutellosaurus*. Also, Wiersma & Irmis (2018) recovered *Scelidosaurus* as the sister taxa of all other Ankylosauria (Ankylosauromorpha sensu Norman, 2020b). However, the inclusion of *Jakapil* generates a polytomy (of grade 3) between *Jakapil*, *Scelidosaurus* and the traditional Ankylosauria (Suppl. Fig. 8). Branch support shows an overall decreasing, although Bootstrap support is not so low for Eurypoda, Stegosauria and Ankylosauromorpha. The considerable decreasing in Ankylosauria Branch support compared with Wiersma & Irmis (2018) results may be related to a closer affinity of *Jakapil* to this clade. This is also supported in the several autapomorphies of *Jakapil* shared with many ankylosaurid taxa. Despite the lower Branch support compared with the results of Wiersma & Irmis (2018), *Jakapil* is well supported within the Ankylosauria along with *Scelidosaurus* in our analysis.


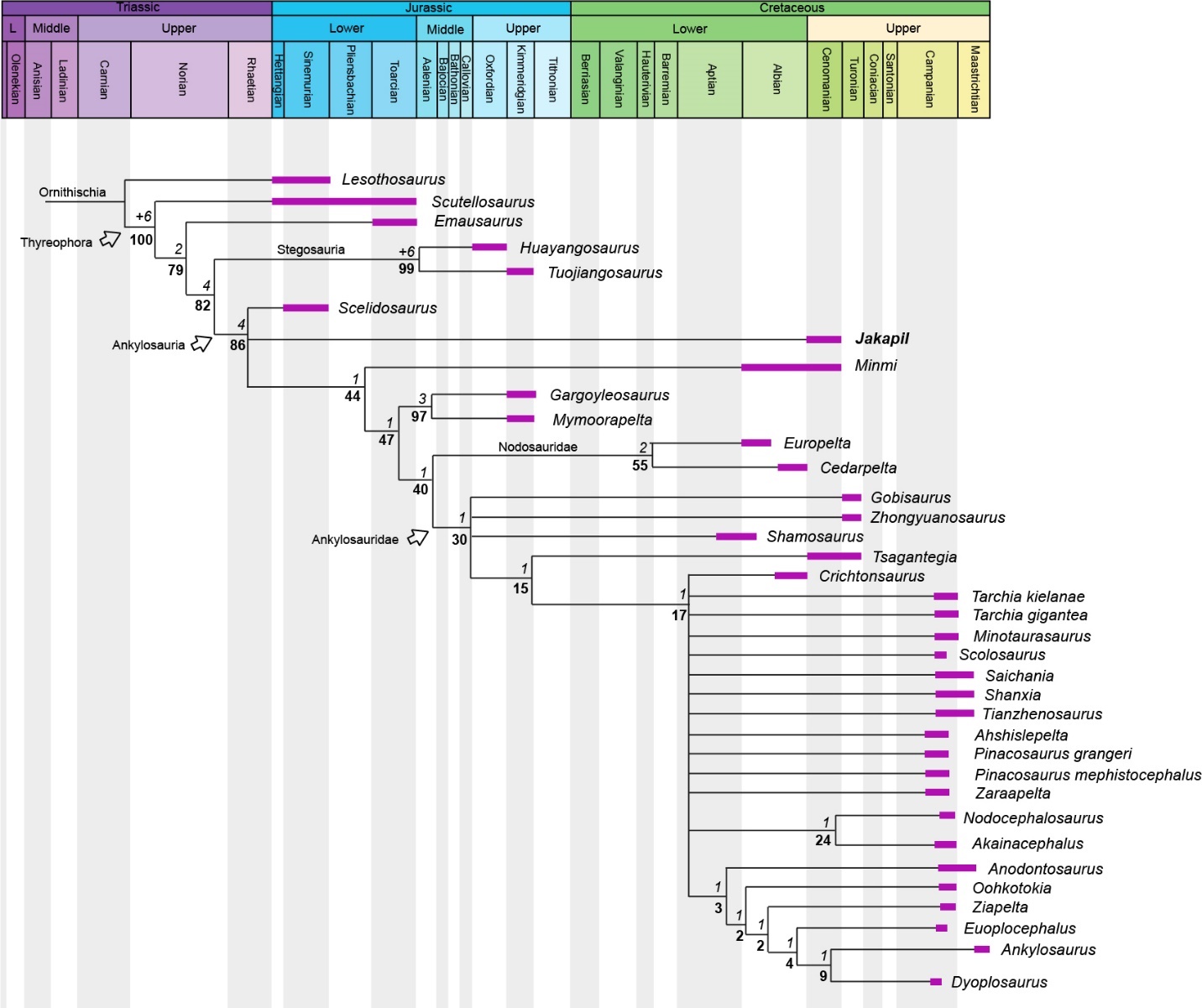


**Supplementary Figure 8**: Time calibrated strict consensus of 40 most parsimonious trees (L=652). CI: 0.421, RI: 0.677. Branch supports are figured (Bremer /Bootstrap). Ages of each record were taken from Kirkland *et al*. (2013), Penkalski (2014), Arbour & Currie (2016), Foster *et al*. (2018), Wiersma & Irmis (2018) and Maidment *et al*. (2020).

**Synapomorphies of Thyreophora present in *Jakapil***

None.

**Synapomorphies of Ankylosauria present in *Jakapil***

Char. 88: 0🡪1: Dentary, size and projection of the dorsal surangular process: well developed with a medially positioned dorsal projection (reverted in the Ankylosaurinae).

Char. 90: 0🡪1: Surangular, coronoid process: present.

Char. 93: 0🡪1: External mandibular fenestra: absent.

Char. 120: 0🡪1: Mandibular ornamentation, presence of ornamentation on lateral surface of mandible: present.

Char. 229: 0🡪1: Cervical armour, fusion of osteoderms on dorsal surface of neck region into neck bands or ‘rings’: sutured together, sometimes to a quarter-ring but not into a half-ring (change to 2 in *Shamosaurus* and the Ankylosaurinae).

**Autapomorphies of *Jakapil***

Chan. 83: 0🡪1: Dentary, depth of the dentary symphysial ramus relative to the maximum depth of the dentary in lateral view: shallow, less than 45% maximum dentary depth (shared with *Ankylosaurus*, *Euoplocephalus*, *Shamosaurus*, *Minotaurosaurus*, *Tarchia*, *Pinacosaurus*, *Anodontosaurus*, *Akainacephalus* and *Saichania*).

Char. 84: 1🡪0: Dentary, shape of dorsal margin of the dentary in lateral view: straight (shared with *Scutellosaurus* and *Lesothosaurus*).

Char. 94: 0🡪1: Premaxilla, premaxillary teeth: absent (shared with *Shamosaurus*, *Gobisaurus*, *Zhongyuanosaurus*, *Tsagantegia*, *Minotaurosaurus*, *Tarchia*, *Saichania*, *Pinacosaurus*, *Tianzhenosaurus*, *Crichtonsaurus*, *Akainacephalus*, *Nodocephalosaurus*, *Anodontosaurus*, *Oohkotokia*, *Ziapelta*, *Euoplocephalus* and *Ankylosaurus*).

Char. 96: 0🡪1: Dentary, teeth extend nearly to the symphysis or predentary contact: absent, there is a diastema between the symphysis and the rostral-most tooth (shared with *Ankylosaurus*, *Euoplocephalus*, *Shamosaurus*, *Minotaurosaurus*, *Tarchia*, *Pinacosaurus*, *Anodontosaurus*, *Akainacephalus* and *Saichania*).

**Data matrix of Maidment *et al*. (2020)**

With the inclusion of *Jakapil*, the data matrix consists of 26 taxa scored for 115 characters and is attached at the end of this archive (1-24, 106 and 107 as additive; see character list in Maidment *et al*., 2020). *Pisanosaurus mertii* was fixed as the outgroup-most taxon. All characters were unweighted. The analysis was made with TNT v1.5 (Goloboff & Catalano, 2016) using the same analytical settings as Maidment *et al*. (2020). Memory space was made for 2,000,000 trees. Searching trees was carried out with a New Technology search using a combination of Sectorial Search, Ratchet, Drift and Tree fusing options, with 10 random addition sequences. The MPTs recovered with the New Technology search were resampled with the tree bisection– reconnection algorithm (TBR). Branch support was calculated using Symmetric Resampling and Bootstrap analysis. Symmetric resampling was applied using 5,000 replicates and a Traditional search (default values). 1,000 pseudoreplicates and Traditional Search were used for Bootstrap support.

The phylogenetic analysis recovers *Jakapil* as the sister taxon of Eurypoda (Stegosauria+Ankylosauria, as defined by Sereno, 1998), along with the successive sister taxa *Scelidosaurus*, *Emausaurus*, and a clade including both *Scutellosaurus* and *Laquintasaura* (Suppl. Fig. 9). These results are consistent with those of Maidment *et al*. (2020). However, as seen in the analysis with the matrix of Norman (2020b), branch support decreases when incorporating *Jakapil*, with the exception of the clade *Laquintasaura* + *Scutellosaurus* (supporting Norman’s proposal for *Scutellosaurus* outside Eurypoda, see Phylogenetic Discussion below).


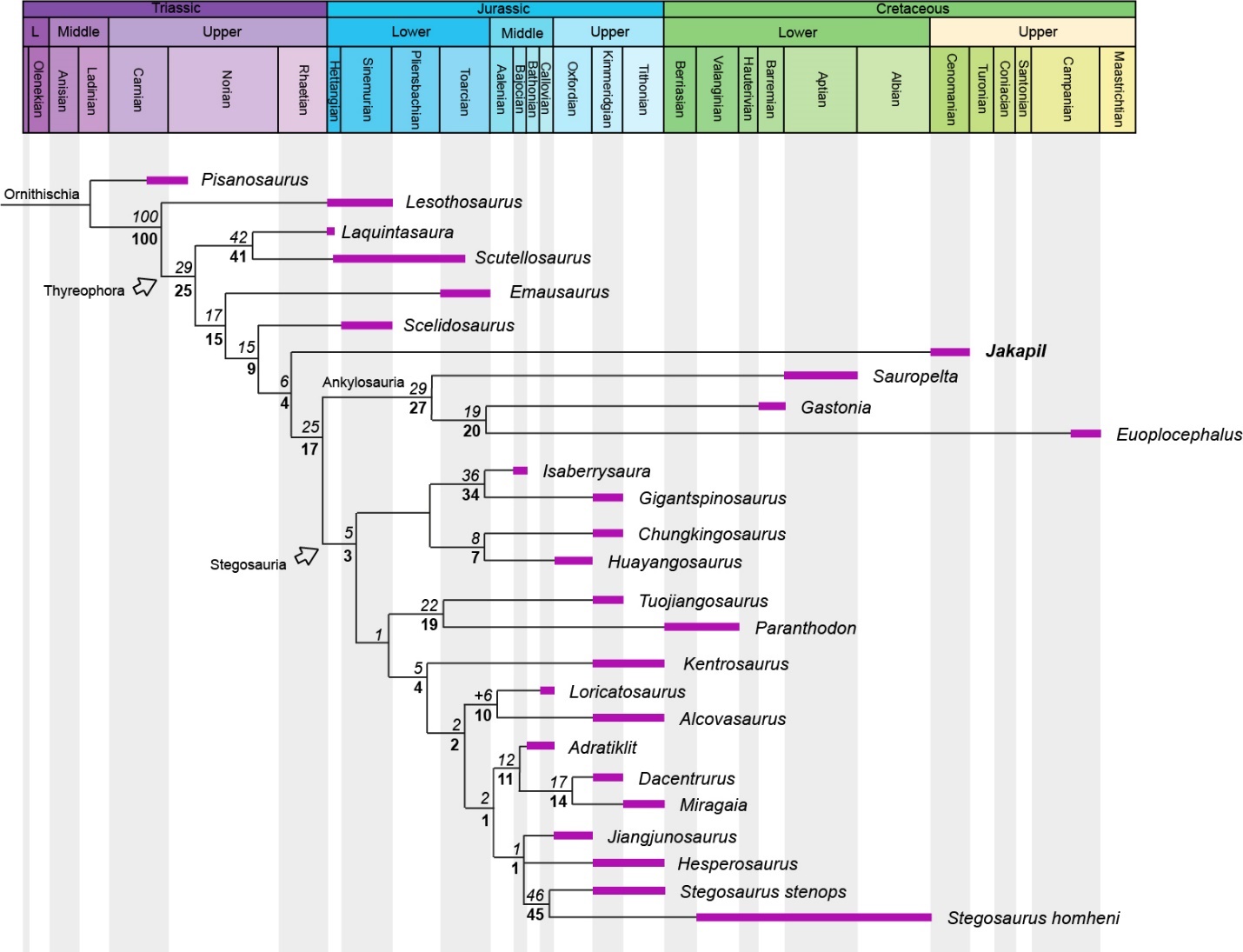


**Supplementary Figure 9**: Time calibrated strict consensus of 2 most parsimonious trees (L=281.66). CI: 0.591, RI: 0.650. Branch supports are figured (Symmetric Resampling /Bootstrap). Ages of each record were taken from Arbour & Currie (2016), Norman (2020b) and Maidment *et al*. (2020).

**Synapomorphies of Thyreophora present in *Jakapil***

Char. 29: 0🡪1: Maxilla, tooth row inset medially from the lateral surface of the maxilla: present.

Under ACCTRAN

Char. 108: 0🡪1: Dermal armour including scutes, and/or spines and/or plates: present.

**Autapomorphies of *Jakapil***

Char. 2 (continuous, ordered): Teeth, number coded as meristic: 11 teeth (relatively low).

Char. 8 (continuous, ordered): Dorsal vertebrae, centrum height to neural arch height ratio coded continuously: 1.6 (relatively high).

Char. 42: 0🡪1: Jaw joint, ventral to tooth row: level with tooth row (shared with S*tegosaurus stenops*).

Char. 46: 1🡪0: Dentary, tooth row in lateral view: straight (shared with *Pisanosaurus*, *Lesothosaurus* and S*tegosaurus stenops*).

Char. 115: 0🡪1: Ossified epaxial tendons: absent (shared with the Stegosauridae).

**Phylogenetic discussion**

Sereno (1998) phylogenetically defined Ankylosauria with a stem-based definition (all taxa closer to *Ankylosaurus* than to *Stegosaurus*). However, Norman (2020b) redefined Ankylosauria as all taxa closer to *Edmontonia* and *Euoplocephalus* than to *Scelidosaurus*, and defined Ankylosauromorpha as all taxa closer to *Edmontonia* and *Euoplocephalus* than to *Stegosaurus* (see also Carpenter, 2001). Since the definition of Sereno (1998) for Ankylosauria would include *Jakapil* and the traditional basal thyreophorans, we consider that the statement of an Ankylosauromorpha group sensu Norman (2020b) is useful to approach taxa with plesiomorphic features quite different from those traditionally used to diagnose ankylosaurs (Vickaryous *et al*., 2004; Arbour & Currie, 2016). We used the traditional Ankylosauria (and Eurypoda; both sensu Sereno, 1998) to be conservative with the literature.

Our topologies with the Soto-Acuña *et al*. (2021) and Maidment *et al.* (2020) datasets recover all the basal thyreophorans outside Eurypoda, in contrast with that of Norman, where all these taxa are included within Ankylosauromorpha. Norman (2020b) argued that, in previous phylogenetic studies (such as Arbour & Currie, 2016, and Maidment *et al*., 2020;designed to test different hypotheses other than *Scelidosaurus* phylogenetic position), taxa like *Scelidosaurus* are mostly ‘absent/0’ coded and excluded from the Ankylosauromorpha. Also, that ankylosaurian datasets (Arbour & Currie, 2016; Wiersma & Irmis, 2018) show high homoplasy, generating a low branch support (Norman, 2020b). However, Norman (2020b) has excluded several basal ankylosaurian and stegosaurian taxa (like *Isaberrysaura*, *Gigantspinosaurus*, *Cedarpelta*, *Gargoyleosaurus*, *Gastonia*, *Peloroplites*, *Polacanthus*, etc.), and their effect on the topology remains to be tested due to the importance of basal taxa in phylogenetic analyses (Raven & Maidment, 2018).

Despite the more inclusive analysis (with the Soto-Acuña dataset) that recovers *Jakapil* as a basal thyreophoran and sister taxon of Eurypoda, a closer position of *Jakapil* to the Ankylosauria than to Stegosauria is suggested by the results obtained with the Wiersma & Irmis (2018) and Norman (2020) datasets. It is supported in the mixture of basal and ankylosaurian-like features presents in *Jakapil* (see the convergences with ankylosaurids listed above). More records are needed to understand better these tendences at the base of Eurypoda.

The overall diminution in branch support (both in thyreophorans and ceratopsians) when *Jakapil* is incorporated into all analyses may be due to the ambiguous features, including plesiomorphic and derived features (homoplasies; see description, synapomorphies and autapomorphies with all datasets), and to the low proportion of coded characters (with Soto-Acuña *et al*. 2021 dataset: 90/383=23.50%; Norman 2020b dataset: 40/115=34.78%; with Wiersma & Irmis 2018 dataset: 64/293=21.84%; with Maidment *et al*. 2020 dataset: 35/115=30.43%).

Whatever the case, an exhaustive discussion of the thyreophoran phylogeny is beyond the scope of this paper. The contrasting results with the used datasets and the general decrease in branch support show that the early diversification of thyreophorans is still poorly understood. *Jakapil kaniukura* shows that Gondwanan thyreophoran diversity is almost entirely unknown, as well as their contribution to the early thyreophoran diversification. *Jakapil* represents an Upper Cretaceous remnant of an older lineage that probably originated during the Lower-Middle Jurassic.

# Supplementary References

**Apesteguía, S. & Novas, F. E**. 2003. Large Cretaceous sphenodontian from Patagonia

provides insight into lepidosaur evolution in Gondwana. *Nature*, **425**, 609-612. DOI: 10.1038/nature01995.

**Apesteguía, S. & Zaher, H.** 2006. Cretaceous terrestrial snake with robust hindlimbs and a sacrum. *Nature*, **440**, 1037–1040. DOI: 10.1038/nature04413.

**Apesteguía, S., Agnolin, F. L. & Lio, G. L.** 2005. An early Late Cretaceous lizard from Patagonia, Argentina. *Comptes Rendus Palevol*, **4**(4), 311-315. DOI: 10.1016/j.crpv.2005.03.003.

**Apesteguía, S., Agnolín, F. L. & Claeson, K.** 2007. Review of Cretaceous dipnoans from Argentina (Sarcopterygii: Dipnoi) with descriptions of new species. *Revista del Museo Argentino de Ciencias Naturales, Nueva Serie*, **9**(1), 27-40.

**Arbour, V. M. & Currie P. J.** 2013. *Euoplocephalus tutus* and the diversity of ankylosaurid dinosaurs in the Late Cretaceous of Alberta, Canada, and Montana, USA. *PLoS ONE*, **8**, e62421. DOI: 10.1371/journal.pone.0062421.

**Arbour, V. M. & Currie, P. J.** 2016. Systematics, phylogeny and palaeobiogeography of the ankylosaurid dinosaurs. *Journal of Systematic Palaeontology*, **14**, 385–444. DOI: 10.1080/14772019.2015.1059985.

**Baron, M. G., Norman, D. B. & Barrett, P. M.** 2017. Postcranial anatomy of *Lesothosaurus* *diagnosticus* (Dinosauria: Ornithischia) from the Lower Jurassic of southern Africa: implications for basal ornithischian taxonomy and systematics. *Zoological Journal of the Linnean Society*, **179**, 125–168. DOI: 10.1111/zoj.12434.

**Barrett, P. M.** 2001. Tooth wear and possible jaw action of *Scelidosaurus harrisonii* Owen and a review of feeding mechanisms in other thyreophoran dinosaurs. In: Carpenter K, ed. *The armored dinosaurs*. Bloomington: Indiana University Press, 25–52.

**Barrett, P. M., Butler, R.J. & Knoll, F.** 2005. Small bodied ornithischian dinosaurs from the Middle Jurassic of Sichuan, China. *Journal of Vertebrate Paleontology*, **25**, 823–834.

**Barrett, P. M., Butler, R. J., Mundil, R., Scheyer, T. M., Irmis, R. B. & Sánchez Villagra, M. R.** 2014 A palaeoequatorial ornithischian and new constraints on early dinosaur diversification. *Proceedings of the Royal Society B*, **281**, 20141147. DOI: 10.1098/rspb.2014.1147.

**Barrett, P. M., Butler, R. J., Yates, A. M., Baron, M. G & Choiniere, J. N.** 2016. New specimens of the basal ornithischian dinosaur *Lesothosaurus diagnosticus* Galton, 1978 from the Early Jurassic of South Africa. *Palaeontologia Africana*, **50**, 48–63. DOI: 10539/19886.

**Barta, D. E. & Norell, M. A.** 2021. The osteology of *Haya griva* (Dinosauria: Ornithischia) from the Late Cretaceous of Mongolia. *Bulletin of the American Museum of Natural History*, **445**, 445.1.1. DOI: 10.1206/0003-0090.445.1.1.

**Benson, R. B., Hunt, G., Carrano, M. T. & Campione, N.** 2018. Cope’s rule and the adaptive landscape of dinosaur body size evolution. *Palaeontology*, **61**, 13–48. DOI: 10.1111/pala.12329.

**Bertozzo, F., Dalla Vecchia, F. M. & Fabbri, M.** 2017. The Venice specimen of *Ouranosaurus*

*nigeriensis* (Dinosauria, Ornithopoda). *PeerJ*, **5**, e3403.

**Blows, W. T.** 2015. British Polacanthid Dinosaurs – Observations on the history and palaeontology of the UK polacanthid armoured dinosaurs and their relatives. 220 pp. Manchester (Siri Scientific Press).

**Boyd, C. A.** 2014. The cranial anatomy of the neornithischian dinosaur *Thescelosaurus neglectus*. *PeerJ*, **2**, e669. DOI 10.7717/peerj.669.

**Boyd, C. A.** 2015. The systematic relationships and biogeographic history of ornithischian dinosaurs. *PeerJ*, **3**, e1523. DOI 10.7717/peerj.1523.

**Breeden, B. T. & Rowe, T. B.** 2020. New specimens of *Scutellosaurus lawleri* Colbert, 1981, from the Lower Jurassic Kayenta Formation in Arizona elucidate the early evolution of thyreophoran dinosaurs. *Journal of Vertebrate Paleontology*, **40**(4), e1791894. DOI:10.1080/02724634.2020.1791894.

**Breeden, B. T., Raven, T. J., Butler, R. J., Rowe, T. B. & Maidment, S. C. R.** 2021. The anatomy and palaeobiology of the early armoured dinosaur *Scutellosaurus lawleri* (Ornithischia: Thyreophora) from the Kayenta Formation (Lower Jurassic) of Arizona. *Royal Society Open Science*, **8**, 201676. DOI: 10.1098/rsos.201676.

**Brown, C.M., Boyd, C.A. & Russell, A.P.** 2011. A new basal ornithopod dinosaur (Frenchman Formation, Saskatchewan, Canada), and implications for late Maastrichtian ornithischian diversity in North America. *Zoological Journal of the Linnean Society*, **163**, 1157–1198. DOI: 10.1111/j.1096-3642.2011.00735.x.

**Burch, S. H.** 2013. Osteological, Myological, and Phylogenetic Trends of Forelimb Reduction in Nonavian Theropod Dinosaurs. PhD Dissertation. Stony Brook University, 407 pp.

**Burns, M. E. & Currie, P. J.** 2014. External and internal structure of ankylosaur (Dinosauria; Ornithischia) osteoderms and their systematic relevance. *Journal of Vertebrate Paleontology*, **34**, 835–851. DOI: 10.1080/02724634.2014.840309.

**Burns, M., Currie, P. J., Sissons, R. L. & Arbour, V. M.** 2011. Juvenile specimens of *Pinacosaurus grangeri* Gilmore, 1933 (Ornithischia: Ankylosauria) from the Late Cretaceous of China, with comments on the specific taxonomy of *Pinacosaurus*. *Cretaceous Research*, **31**(2), 174 –186. DOI: 10.1017/jpa.2014.14.

**Burns, M. E.** 2015. [Intraspecific variation in the armoured dinosaurs (Dinosauria: Ankylosauria)](https://scholar.google.com/scholar_lookup?title=Intraspecific%20variation%20in%20the%20armoured%20dinosaurs%20(Dinosauria:%20Ankylosauria)&author=Burns&publication_year=2015) PhD dissertation, University of Alberta.

**Butler, R. J., Upchurch, P. & Norman, D. B.** 2008. The phylogeny of the ornithischian dinosaurs. *Journal of Systematic Palaeontology*, **6**, 1–40. DOI: 10.1017/S1477201907002271.

**Butler, R. J. & Zhao, Q.** 2009. The small-bodied ornithischian dinosaurs *Micropachycephalosaurus hongtuyanensis* and *Wannanosaurus yansiensis* from the Late Cretaceous of China. *Cretaceous Research*, **30**, 63–77.

**Butler, R. J.** 2010. The anatomy of the basal ornithischian dinosaur *Eocursor parvus* from the lower Elliot Formation (Late Triassic) of South Africa. *Zoological Journal Linnean Society,* **160**, 648-684. DOI: 10.1111/j.1096-3642.2009.00631.x.

**Candia Halupczok, D. J., Sánchez, M. L., Veiga, G. D. & Apesteguía, S.** 2018. Dinosaur tracks in the Kokorkom Desert, Candeleros Formation (Cenomanian, Upper Cretaceous), Patagonia Argentina: Implications for deformation structures in dune fields. *Cretaceous Research*, **83**, 194-206. DOI: 10.1016/j.cretres.2017.10.017.

**Carpenter, K.** 2001. Phylogenetic analysis of the Ankylosauria. In: Carpenter K, ed. *The armored dinosaurs*. Bloomington: Indiana University Press, 455–483.

**Carpenter, K.** 2004. Redescription of *Ankylosaurus magniventris* Brown 1908 (Ankylosauridae) from the Upper Cretaceous of the Western Interior of North America. *Canadian
Journal of Earth Sciences*, **41**, 961986. DOI: 10.1139/e04-043.

**Carpenter, K., DiCroce, T., Kinneer, B. & Simon, R.** 2013. Pelvis of *Gargoyleosaurus* (Dinosauria: Ankylosauria) and the origin and evolution of the ankylosaur pelvis. *PLoS ONE*, **8**, e79887. DOI: 10.1371/journal.pone.0079887.

**Cerda, I. A. & Powell, J.E.** 2010. Dermal armor histology of *Saltasaurus loricatus*, an Upper Cretaceous sauropod dinosaur from Northwest Argentina. *Acta Palaeontologica Polonica,* **55**(3), 389–398. DOI: 10.4202/app.2009.1101.

**Cerda, I. A., Gasparini, Z., Coria, R., Salgado, L., Reguero, M., Ponce, D., González, R., Janello, M. J. & Moly, J.** 2019. Paleobiological inferences for the Antarctic dinosaur *Antarctopelta oliveroi* (Ornithischia: Ankylosauria) based on bone histology of the holotype. *Cretaceous Research,* **103**, 104171. DOI: 10.1016/j.cretres.219.07.001.

**Cerda, I. A., Pereyra, M. E., Garrone, M., Ponce, D., Navarro, T. G., González, R., Militello, M., Luna, C. A. & Jannello, J. M.** 2020. A basic guide for sampling and preparation of extant and fossil bones for histological studies. *Publicación Electrónica de la Asociación Paleontológica Argentina*, **20**(1), 15–28. DOI: 10.5710/PEAPA.07.04.2020.314.

**Chinsamy-Turan, A.** 2011. Dinosaur Growth: Egg to Adult. In: *Encyclopedia of Life Sciences* (ELS). John Wiley & Sons: Chichester. DOI: 10.1002/9780470015902.a0003319.pub2

**Colbert, E. H.** 1981. A primitive ornithischian dinosaur from the Kayenta Formation of Arizona. *Museum of Northern Arizona Bulletin*, **53**, 1–61.

**Coombs, W. P.** 1986. A juvenile ankylosaur referable to the genus *Euoplocephalus* (Reptilia, Ornithischia). *Journal of Vertebrate Palaeontology*, **6**, 162–173. DOI: 10.1080/02724634.1986.10011608.

**Corbella, H., Novas, F. E., Apesteguía, S. & Leanza, H. A.** 2004. First fision track age for the dinosaur-bearing Neuquén Group (Upper Cretaceous), Neuquén Basin, Argentina. *Revista del Museo de Ciencias Naturales, Nueva Serie*, **6**(2), 227–232.

**Cruzado-Caballero, P. & Powell, J.** 2017. *Bonapartesaurus rionegrensis*, a new hadrosaurine dinosaur from South America: implications for phylogenetic and biogeographic relations with North America. *Journal of Vertebrate Paleontology*. DOI: 10.1080/02724634.2017.1289381.

**Dieudonné, P. E., Cruzado-Caballero, P., Godefroit, P. & Tortosa, T.** 2020. A new phylogeny of cerapodan dinosaurs. *Historical Biology*. DOI: 10.1080/08912963.2020.1793979.

**Erickson, G. M., Curry Rogers, K., Varricchio, D. J., Norell, M. A. & Xu, X.** 2007. Growth patterns in brooding dinosaurs reveals the timing of sexual maturity in non-avian dinosaurs and genesis of the avian condition. *Biology Letters*, **3**, 558–61. DOI: 10.1098/rsbl.2007.0254.

**Fernández Dumont, M. L., Bona, P., Pol, D. & Apesteguía, S.** 2020. New anatomical information on *Araripesuchus buitreraensis* with implications for the systematics of Uruguaysuchidae (Crocodyliforms, Notosuchia), *Cretaceous Research*, 113. 104494. DOI: 10.1016/j.cretres.2020.104494.

**Ford, T. L.** 2000. A review of ankylosaur osteoderms from New Mexico and a preliminary review of ankylosaur armor. *New Mexico Museum of Natural History and Science Bulletin*, **17**, 157–176.

**Foster, J. R., Hunt-Foster, R. K., Gorman II, M. A., Trujillo, K. C., Suarez, C. A., McHugh, J. B., Peterson, J. E., Warnock, J. P. & Schoenstein, H. E.** 2018.
Paleontology, taphonomy, and sedimentology of the Mygatt-Moore Quarry, a large dinosaur bonebed in the Morrison Formation, western Colorado—implications for Upper Jurassic dinosaur preservation modes. *Geology of the Intermountain West*, **5**, 23–93. DOI: 10.31711/giw.v5.pp23-93.

**Galton, P. M.** 1974. The ornithischian dinosaur *Hypsilophodon* from the Wealden of the Isle of Wight. *Bulletin of the British Museum (Natural History), Geology,* **25**, 1–152c.

**Galton, P. M.** 1982a. Juveniles of the stegosaurian dinosaur *Stegosaurus* from the Upper Jurassic of North America. *Journal of Vertebrate Paleontology*, **2**, 47–62. DOI: 10.1080/02724634.1982.10011917.

**Galton, P. M.** 1982b. The postcranial anatomy of stegosaurian dinosaur *Kentrosaurus* from the Upper Jurassic of Tanzania, East Africa. *Geologica et Paleontologica*, **15**, 139–160.

**Galton, P. & Upchurch, P.** 2004. Stegosauria. In: Weishampel DB, Dodson P & Osmolska H, eds. *The Dinosauria*, second edition. Berkeley: University of California Press, 343–362.

**Garberoglio, F. F., Gómez, R., Apesteguía, S., Caldwell, M. W., Sánchez M. L. & Veiga, G. D.** 2019a. A new specimen with skull and vertebrae of *Najash rionegrina* (Lepidosauria: Ophidia) from the early Late Cretaceous of Patagonia. *Journal of Systematic Palaeontology*, **17**, 1313–1330. DOI: 10.1080/14772019.2018.1534288.

**Garberoglio, F. F., Apesteguía, S., Simões, T. R., Palci, A., Gómez, R. O., Nydam, R. L., Larsson, H. C. E., Lee, M. S. Y., & Caldwell, M. W.** 2019b. New skulls and skeletons of the Cretaceous legged snake *Najash*, and the evolution of the modern snake body plan. *Science Advances*, **5**, eaax5833. DOI :10.1126/sciadv.aax5833.

**Garrido, A. C.** 2010. Estratigrafía del Grupo Neuquén, Cretácico Superior de la Cuenca Neuquina (República Argentina): Nueva propuesta de ordenamiento litoestratigráfico. *Revista del Museo Argentino de Ciencias Naturales, Nueva Serie*, **12**(2), 121–177.

**Gilmore, C. W.** 1914. Osteology of the armored Dinosauria in the U.S. National Museum, with special reference to Stegosaurus. *United States National Museum Bulletin*, **89**, 1–136. DOI: 10.5962/bhl.title.63658.

**Gilmore, C. W.** 1920. Osteology of the carnivorous Dinosauria in the United States National Museum, with special reference to the genera *Antrodemus* (*Allosaurus*) and *Ceratosaurus*. *Bulletin of the United States National Museum*, **110**, 1–159. DOI: 10.5479/si.03629236.110.i.

**Goloboff, P. & Catalano, S.** 2016. TNT version 1.5, including a full implementation of phylogenetic morphometrics. *Cladistics*, **32**, 221–238. DOI: 10.1111/cla.12160.

**Han, F.-L., Forster, C. A., Clark, J. M. & Xu, X.** 2016. Cranial anatomy of *Yinlong downsi* (Ornithischia: Ceratopsia) from the Upper Jurassic Shishugou Formation of Xinjiang, China. *Journal of Vertebrate Paleontology*, **36**, e1029579. DOI: 10.1080/02724634.2015.1029579.

**Han, F.-L., Forster, C. A, Xu, X. & Clark, J. M.** 2018. Postcranial anatomy of *Yinlong downsi* (Dinosauria: Ceratopsia) from the Upper Jurassic Shishugou Formation of China and the phylogeny of basal ornithischians. *Journal of Systematic Palaeontology*, **16**, 1159–1187. DOI: 10.1080/14772019.2017.1369185

**Haubold, H.** 1990. Ein neuer Dinosaurier (Ornithischia, Thyreophora) aus dem unteren Jura des nördlichen Mitteleuropa. *Revue de Paléobiologie*, **9**, 149–177.

**Hayashi, S., Carpenter, K. & Suzuku, D.** 2009. Different growth patterns between the skeleton and osteoderms of *Stegosaurus* (Ornithischia: Thyreophora). *Journal of Vertebrate Paleontology*, **29**, 123–131. DOI: 10.1080/02724634.2009.10010366.

**Kilbourne, B. & Carpenter, K.** 2005. Redescription of *Gargoyleosaurus parkpinorum*, a polacanthid ankylosaur from the Upper Jurassic of Albany County, Wyoming. *Neues Jahrbuch für Geologie und Paläontologie, Abhandlungen*, **237**, 111–160. DOI: 10.1127/njgpa/235/2005/111.

**Kinneer, B., Carpenter, K. & Shaw, A.** 2016. Redescription of *Gastonia burgei* (dinosauria: Ankylosauria, Polacanthidae), and description of a new species. *Neues Jahrbuch für Geologie und Paläontologie, Abhandlungen*, **282**(1), 37–80. DOI: 10.1127/njgpa/2016/0605.

**Kirkland, J. I., Alcalá, L., Loewen, M. A., Espílez, E., Mampel, L. & Wiersma, J. P.** 2013. The Basal Nodosaurid Ankylosaur *Europelta carbonensis* n. gen., n. sp. from the Lower
Cretaceous (Lower Albian) Escucha Formation of Northeastern Spain. *PLoS ONE*, **8**(12), e80405. DOI: 10.1371/journal.pone.0080405.

Lambert, O., Godefroit, P., Li H., Shang, C.-Y. & Dong, Z.-M. 2001. A new species of *Protoceratops* (Dinosauria, Neoceratopsia) from the Late Cretaceous of Inner Mongolia. *Bulletin de L’Institut Royal Des Sciences Naturelles De Belgique*, **71**, 5–28.

**Leahey, L. G., Molnar, R. E., Carpenter, K., Witmer, L. M. & Salisbury, S. W.** 2015. Cranial osteology of the ankylosaurian dinosaur formerly known as *Minmi* sp. (Ornithischia: Thyreophora) from the Lower Cretaceous Allaru Mudstone of Richmond, Queensland, Australia. *PeerJ*, **3**, e1475l. DOI: 10.7717/peerj.1475.

**Leanza, H. A.** 2009. Las principales discordancias del Mesozoico de la Cuenca Neuquina según observaciones de superficie. *Revista del Museo Argentino de Ciencias Naturales, Nueva Serie*, **11**(2), 145-184.

**Leanza, H., Apesteguía, S., Novas, F. E. & de la Fuente, M. S.** 2004. Cretaceous terrestrial beds from the Neuquén Basin (Argentina) and their tetrapod assemblages. *Cretaceous Research*,
**25**, 61-87. DOI: 10.1016/j.cretres.2003.10.005.

**Lü, J., Ji, Q., Gao, Y. & Li, Z.** 2007. A new species of the ankylosaurid dinosaur *Crichtonsaurus* (Ankylosauridae: Ankylosauria) from the Cretaceous of Liaoning Province, China. *Acta Geologica Sinica - English Edition*, **81**, 883–897. DOI: 10.1111/j.1755-6724.2007.tb01010.x.

**Makovicky, P. J., Apesteguía, S. & Agnolin, F. L.** 2005. The earliest dromaeosaurid theropod from South America. *Nature*, **437**, 1007-1011. DOI: 10.1038/nature03996.

**Makovicky, P. J., Apesteguía, S. & Gianechini, F. A.** 2012. A new coelurosaurian theropod from the La Buitrera fossil locality of Río Negro. Argentina. *Fieldiana Life and Earth Sciences*, **5**, 90-98. DOI: 10.3158/2158-5520-5.1.90.

**Maidment, S. C. R., Wei, S. & Norman, D. B.** 2006. Re-description of the postcranial skeleton of the Middle Jurassic stegosaur *Huayangosaurus taibaii*. *Journal of Vertebrate Paleontology*, **26**, 944–956. DOI: 10.1671/0272-4634(2006)26[944:ROTPSO]2.0.CO;2.

**Maidment, S. C. R., Norman, D. B., Barrett, P. M. & Upchurch, P.** 2008. Systematics and phylogeny of Stegosauria (Dinosauria: Ornithischia). *Journal of Systematic Palaeontology*, **6**, 364–407. DOI: 10.1017/S1477201908002459.

**Maidment, S. C. R. & Barrett, P. M**. 2014. Osteological correlates for quadrupedality in ornithischian dinosaurs. *Acta Palaeontologica Polonica*, **59**, 53–70. DOI: 10.4202/app.2012.0065.

**Maidment, S. C. R., Brassey, C. & Barrett, P. M**. 2015. The Postcranial Skeleton of an Exceptionally Complete Individual of the Plated Dinosaur *Stegosaurus stenops* (Dinosauria: Thyreophora) from the Upper Jurassic Morrison Formation of Wyoming, U.S.A. *PLoS ONE*, **10**(10), e0138352. doi:10.1371/journal.pone.0138352.

**Maidment, S. C. R., Raven, T. J., Ouarhache, D. & Barrett, P. M.** 2020. North Africa’s first stegosaur: Implications for Gondwanan thyreophoran dinosaur diversity. *Gondwana Research*, **77**, 82–97. DOI: 10.1016/j.gr.2019.07.007.

**Main, R. P., de Ricqlès, A., Horner, J. R. & Padian, K.** 2005. The evolution and function of thyreophoran dinosaur scutes: implications for plate function in stegosaurs. *Paleobiology*, **31**, 291–314. DOI:10.1666/0094-8373(2005)031[0291:TEAFOT]2.0.CO;2.

**Maiorino, L., Farke, A. A., Kotsakis, T. & Piras, P.** 2017 Macroevolutionary patterns in cranial and lower jaw shape of ceratopsian dinosaurs (Dinosauria: Ornithischia): phylogeny, morphological integration and evolutionary rates. *Evolutionary Ecology Research*, **18**, 123– 167.

**Mallon, J. C. & Holmes, R.** 2010. Description of a complete and fully articulated chasmosaurine postcranium previously assigned to *Anchiceratops* (Dinosauria: Ceratopsia). In: Ryan MJ, Chinnery-Allgeier BJ, and Eberth DA, eds. *New Perspectives on Horned Dinosaurs: The Royal Tyrell Museum Ceratopsian Symposium*. Indiana University Press, Bloomington, Indiana, 189–202.

**Mallon, J. C. & Anderson, J. S**. 2014. The Functional and Palaeoecological Implications of Tooth Morphology and Wear for the Megaherbivorous Dinosaurs from the Dinosaur Park Formation (Upper Campanian) of Alberta, Canada. *PLoS ONE*, **9**(6), e98605. DOI: 10.1371/journal.pone.0098605.

**Maniel, I. J., de la Fuente, M. S., Apesteguía, S., Pérez Mayoral, J., Sánchez M. L., Veiga, G. D. & Smales, I.** 2020. Cranial and postcranial remains of a new species of *Prochelidella* (Testudines: Pleurodira: Chelidae) from ‘La Buitrera’ (Cenomanian of Patagonia, Argentina), with comments on the monophyly of this extinct chelid genus from southern Gondwana. *Journal of Systematic Palaeontology*, **20**(12), 1033–1055. DOI: 10.1080/14772019.2020.1721579.

**Maryańska, T.** 1977. Results of the Polish-Mongolian palaeontological expeditions. Part VII. Ankylosauridae (Dinosauria) from Mongolia. *Palaeontologia Polonica*, **37**, 85–151.

**McDonald, A. T., Bird, J., Kirkland, J. I. & Dodson, P**. 2012. Osteology of the Basal Hadrosauroid *Eolambia caroljonesa* (Dinosauria: Ornithopoda) from the Cedar Mountain Formation of Utah. *PLoS ONE*, **7**(10), e45712. DOI:10.1371/journal.pone.0045712.

**Middleton, K. M. & Gatesy, S. M.** 2000. Theropod forelimb design and evolution. *Zoological Journal of the Linnean Society*, **128**, 149–187. DOI: 10.1111/j.1096-3642.2000.tb00160.x.

**Molnar, R. E.** 2001. Armour of the small ankylosaur *Minmi*. Armour of the small ankylosaur *Minmi*. In Carpenter K, ed. *The armored dinosaurs*. Bloomington: Indiana University Press, 341–362.

**Morschhauser, E. M., You, H., Li, D. & Dodson, P**. 2018. Postcranial morphology of the basal neoceratopsian (Ornithischia: Ceratopsia) *Auroraceratops rugosus* from the Early Cretaceous (Aptian–Albian) of northwestern Gansu Province, China. *Journal of Vertebrate Paleontology*, **38**:sup1, 75-116. DOI: 10.1080/02724634.2018.1524383.

**Müller, R. T. & Garcia, M. S.** 2020 A paraphyletic ‘Silesauridae’ as an alternative hypothesis for the initial radiation of ornithischian dinosaurs. *Biology Letters*, **16**, 20200417. DOI: 10.1098/rsbl.2020.0417.

**Norman, D. B.** 2002. On Asian ornithopods (Dinosauria: Ornithischia). 4. *Probactrosaurus* Rozhdestvensky, 1966. *Zoological Journal of the Linnean Society*, **136**, 113–144.

**Norman, D. B., Witmer, L. M. & Weishampel, D. B.** 2004a. Basal Thyreophora. In: Weishampel DB, Dodson P, Osmólska H, eds. *The Dinosauria*, second edition. Berkeley: University of California Press, 335–342.

**Norman, D. B, Witmer, L. M. & Weishampel, D. B. 2004b.** Basal ornithischia. In: Weishampel DB, Dodson P, Osmólska H, eds. *The Dinosauria*, second edition. Berkeley: University of California Press, 325–334.

**Norman, D. B., Crompton, A. W., Butler, R. J., Porro, L. B. & Charig, A. J.** 2011. The Lower Jurassic ornithischian dinosaur *Heterodontosaurus tucki* Crompton & Charig, 1962: cranial anatomy, functional morphology, taxonomy, and relationships. *Zoological Journal of the Linnean Society*, **163**, 182–276. DOI: 10.1111/j.1096-3642.2011.00697.x.

**Norman, D. B.** 2020a. *Scelidosaurus harrisonii* Owen, 1861 (Dinosauria: Ornithischia) from the Early Jurassic of Dorset, England: cranial anatomy. *Zoological Journal of the Linnean Society*, **188**, 1–81. DOI: 10.1093/zoolinnean/zlz074.

**Norman, D. B.** 2020b. *Scelidosaurus harrisonii* Owen, 1861 (Dinosauria: Ornithischia) from the Early Jurassic of Dorset, England: biology and phylogenetic relationships. *Zoological Journal of the Linnean Society*, **191**, 1–86. DOI: 10.1093/zoolinnean/zlaa061.

**Norman, D. B.** 2020c. *Scelidosaurus harrisonii* Owen, 1861 (Dinosauria: Ornithischia) from the Early Jurassic of Dorset, England: postcranial endoskeleton. *Zoological Journal of the Linnean Society*, **189**, 47–157. DOI: 10.1093/zoolinnean/zlz078.

**Norman, D. B.** 2020d. *Scelidosaurus harrisonii* Owen, 1861 (Dinosauria: Ornithischia) from the Early Jurassic of Dorset, England: dermal skeleton. *Zoological Journal of the Linnean Society*, **190**, 1–53. DOI: 10.1093/zoolinnean/zlz085.

**Ohashi, T. & Barrett, P. M.** 2009. A new ornithischian dinosaur from the Lower Cretaceous Kuwajima Formation of Japan. *Journal of Vertebrate Paleontology*, **29**(3), 748-757. DOI: 10.1671/039.029.0306.

**Ostrom, J. H.** 1970. Stratigraphy and paleontology of the Cloverly Formation (Lower Cretaceous) of the Big Horn Basin area, Wyoming and Montana. *Peabody Museum of Natural History Bulletin*, **35**, 1–234. ISBN: 9781933789422.

**Ősi, A.** 2005. *Hungarosaurus tormai*, a new ankylosaur (Dinosauria) from the Upper Cretaceous of Hungary. *Journal of Vertebrate Paleontology*, **25**, 370383. DOI: 10.1671/0272-4634(2005)025[0370:HTANAD]2.0.CO;2.

**Ősi, A. & Prondvai, E. 2013.** Sympatry of two ankylosaurs (*Hungarosaurus* and cf. *Struthiosaurus*) in the Santonian of Hungary. *Cretaceous Research,* **44**, 58–63. DOI: 10.1016/j.cretres.2013.03.006.

**Ősi, A., Prondvai, E., Mallon, J. & Bodor, E. R.** 2016. Diversity and convergences in the evolution of feeding adaptations in ankylosaurs (Dinosauria: Ornithischia). *Historical Biology*, **29**, 539–570. DOI: 10.1080/08912963.2016.1208194.

**Padian, K. & Woodward, H. N.** 2021. Archosauromorpha: Avemetatarsalia – Dinosaurs and Their Relatives. In: de Buffrénil, V., de Ricqlès, A. J., Zylberberg, L. & Padian, K., eds. *Vertebrate Skeletal Histology and Paleohistology*, first edition. Boca Raton: CRC Press, 511–549. DOI: 10.1201/9781351189590.

**Padian, K., Horner, J.R. & de Ricqlès, A.** 2004. Growth in small dinosaurs and pterosaurs: The evolution of archosaurian growth strategies. *Journal of Vertebrate Paleontology*, **24**, 555–571. DOI: 10.1671/0272-4634(2004)024[0555:GISDAP]2.0.CO;2.

**Park, J-Y., Lee Y-N., Currie, P. J., Ryan, M. J., Bell, P., Sissons, R., Koppelhus, E. B, Barsbold, R., Lee, S. & Kim, S-H.** 2021. A new ankylosaurid skeleton from the Upper Cretaceous Baruungoyot Formation of Mongolia: its implications for ankylosaurid postcranial evolution. *Scientific Reports*, **11**, 4101. DOI: 10.1038/s41598-021-83568-4.

**Peng, G.** 1992. Jurassic ornithopod *Agilisaurus louderbacki* (Ornithopoda: Fabrosauridae) from Zigong, Sichuan, China. *Vertebrata PalAsiatica,* **30**, 39–51. [in Chinese, with English summary].

**Penkalski, P.** 2014. A new ankylosaurid from the late Cretaceous Two Medicine Formation of Montana, USA. *Acta Palaeontologica Polonica*, **59**(3), 617–634. DOI: 10.4202/app.2012.0125.

**Pérez-Mayoral, J., Argüello Scotti, A., Apesteguía, S. & Veiga, G.D.** 2021 High-resolution analysis of an erg-margin system from the Cretaceous Candeleros Formation (La Buitrera Paleontological Area, Río Negro Province, Argentina): an approach to different scales of fluvial-aeolian interactions. *Latin American Journal of Sedimentology and Basin Analysis*, **28**(1), 37–59.

**Pol, D. & Apesteguía, S.** 2005. New *Araripesuchus* remains from the early Late Cretaceous (Cenomanian-Turonian) of Patagonia. *American Museum Novitates*, **3490**, 1–38. DOI: 10.1206/3490.1.

**Pol, D., Rauhut, O. W. M. & Becerra, M**. 2011. A Middle Jurassic heterodontosaurid dinosaur from Patagonia and the evolution of heterodontosaurids. *Naturwissenschaften*, **98**, 369–379.

**Porro, L. B., Witmer, L. M. & Barrett, P. M.** 2015. Digital preparation and osteology of the skull of *Lesothosaurus diagnosticus* (Ornithischia: Dinosauria). *PeerJ*, **3**, e1494. DOI: 10.7717/peerj.1494.

**Raven, T. J. & Maidment, S. C. R.** 2017. A new phylogeny of Stegosauria (Dinosauria: Ornithischia). *Palaeontology*, **60**, 401–408. DOI: 10.1111/pala.12291.

**Raven, T. J. & Maidment, S. C. R.** 2018. The systematic position of the enigmatic thyreophoran dinosaur *Paranthodon africanus*, and the use of basal exemplifiers in phylogenetic

analysis. *PeerJ*, **6**, e4529. DOI: 10.7717/peerj.4529.

**Raven, T. J., Barrett, P. M. & Maidment, S. C. R.** 2019. A reassessment of the purported ankylosaurian dinosaur *Bienosaurus lufengensis* from the Lower Lufeng of Yunnan, China. *Acta Palaeontologica Polonica*, **64**, 335–342. DOI: 10.4202/app.00577.2018.

**Rougier, G. W., Apesteguía, S. & Gaetano, L. C.** 2011. Highly specialized mammalian skulls from the Late Cretaceous of South America. *Nature*, **479**, 97–102. DOI: 10.1038/nature10591.

**Ruiz-Omeñaca, J. I., Canudo, J. I., Cuenca-Bescós, G., Cruzado-Caballero, P., Gasca, J. M. & Moreno-Azanza, M.** 2012. A new basal ornithopod dinosaur from the Barremian of Galve, Spain. *Comptes Rendus Palevol*, **11**, 435–444. DOI: 10.1016/j.crpv.2012.06.001.

**Salgado, L., Canudo, J. I., Garrido, A. C., Morenoazanza, M., Martınez, L. C., Coria, R. A. & Gasca, J. M.** 2017. A new primitive Neornithischian dinosaur from the Jurassic of Patagonia with gut contents. *Scientific Reports*, **7**, 42778. DOI: 10.1038/srep42778.

**Saneyoshi, M., Watabe, M., Suzuki, S. & Tsogtbaatar, K.** 2011. Trace fossils on dinosaur bones from Upper Cretaceous eolian deposits in Mongolia: taphonomic interpretation of paleoecosystems in ancient desert environments. *Palaeogeography, Palaeoclimatology, Palaeoecology,* **311,** 38–47. DOI: 10.1016/j.palaeo.2011.07.024.

**Scheyer, T. M. & P. M. Sander.** 2004. Histology of ankylosaur osteoderms: implications for systematics and function. *Journal of Vertebrate Paleontology*, **24**, 874–893. DOI: 10.1671/0272-4634(2004)024[0874:HOAOIF]2.0.CO;2.

**Sereno, P. C.** 1998. A rationale for phylogenetic definitions, with application to the higher-level taxonomy of Dinosauria. *Neues Jahrbuch für Geologie und Paläontologie, Abhandlungen*, **210**, 41–83. DOI: 10.1127/njgpa/210/1998/41.

**Sereno, P. C.** 1991. *Lesothosaurus*, ‘fabrosaurids’ and the early evolution of Ornithischia. *Journal of Vertebrate Paleontology*, **11**, 234–256. DOI: 10.1080/02724634.1991.10011386.

**Sereno, P. C.** 2012. Taxonomy, morphology, masticatory function and phylogeny of heterodontosaurid dinosaurs. *ZooKeys*, **226**, 225. DOI: 10.3897/zookeys.226.2840.

**Sereno, P. C. & Dong, Z.** 1992. The skull of the basal stegosaur *Huayangosaurus taibaii*. *Journal of Vertebrate Paleontology*, **11**, 318–343. DOI: 10.1080/02724634.1992.10011463.

**Sissons, R. L.** 2011. Ankylosaur (Dinosauria, Ankylosauria) foot morphology and an assessment of the function of the limbs and feet (Master Thesis). University of Alberta, 187 pp. DOI: 10.7939/R3MS3K89G.

**Soto-Acuña, S., Vargas, A. O., Kaluza, J., Leppe, M. A., Botelho, J. F., Palma-Liberona, J., Simon-Gutstein, C., Fernández, R. A., Ortiz, H., Milla, V., Aravena, B., Manríquez, L. M. E., Alarcón-Muñoz, J., Pino, J. P., Trevisan, C., Mansilla, H., Hinojosa, L. F., Muñoz-Walther, V. & Rubilar-Rogers, D.** 2021. Bizarre tail weaponry in a transitional ankylosaur from subantarctic Chile. *Nature*. DOI: 10.1038/s41586-021-04147-1.**Stein, M., Hayashi, S. & Sander, P.M.** 2013. Long bone histology and growth patterns in ankylosaurs: Implications for life history and evolution. *PLoS ONE*, **8**, e68590. DOI: 10.1371/journal.pone.0068590.

**Tanoue, K., You, H.-L. & Dodson, P..** 2009. Comparative anatomy of selected basal ceratopsian dentitions. *Canadian Journal of Earth Sciences*, **46**, 425–439. DOI: 10.1139/e09-030.

**Tumanova, T. A.** 1987. The armored dinosaurs of Mongolia [in Russian]. *The Joint Soviet-Mongolian Paleontological Expedition,* **32,** 1–80.

**Tumanova, T. A.** 2000. Armoured dinosaurs from the Cretaceous of Mongolia. In: Benton MJ, Shishkin MA, Unwin DM & Kurochkin EN, eds. *The Age of Dinosaurs in Russia and Mongolia*: Cambridge University Press, Cambridge, 517-532.

**Vickaryous, M. K. & Russell, A. P.** 2003. A redescription of the skull of *Euoplocephalus* tutus (Archosauria: Ornithischia): a foundation for comparative and systematic studies of ankylosaurian dinosaurs. *Zoological Journal of the Linnean Society*, **137**, 157–186. DOI: [10.1046/j.1096-3642.2003.00045.x](https://doi.org/10.1046/j.1096-3642.2003.00045.x).

**Vickaryous, M. K., Maryańska, T. & Weishampel, D. B.** 2004. Ankylosauria. In: Weishampel DB, Dodson P, Osmólska H, eds. *The Dinosauria*, second edition. Berkeley: University of California Press, 363–392.

**Wiersma, J. P. & Irmis, R. B.** 2018. A new southern Laramidian ankylosaurid, *Akainacephalus johnsoni* gen. et sp. nov., from the Upper Campanian Kaiparowits Formation of southern Utah. *PeerJ*, **6**, e5016. DOI: 10.7717/peerj.5016.

**Xu, X., Makovicky, P. J., Wang, X. L., Norell, M. A. & You, H. L.** 2002. A ceratopsian dinosaur from China and the early evolution of Ceratopsia. *Nature*, **416**, 314–317. DOI: 10.1038/416314a.

**You, H.-L. & Dodson, P.** 2003. Redescription of neoceratopsian dinosaur *Archaeoceratops* and early evolution of Neoceratopsia. *Acta Palaeontologica Polonica,* **48**(2), 261–272.

**You, H.-L. & P. Dodson.** 2004. Basal Ceratopsia. In: Weishampel DB, Dodson P, Osmólska H, eds. *The Dinosauria*, second edition. Berkeley: University of California Press, 478–493.

**You, H.-L., K. Tanoue & P. Dodson.** 2007. A new specimen of *Liaoceratops yanzigouensis* (Dinosauria: Neoceratopsia) from the Early Cretaceous of Liaoning Province, China). *Acta Geologica Sinica*, **81**, 898–904. DOI: 10.1111/j.1755-6724.2007.tb01011.x.

**You, H.-L., Tanoue, K. & Dodson, P.** 2008. New data on cranial anatomy of the ceratopsian dinosaur *Psittacosaurus major*. *Acta Palaeontologica Polonica*, **53**(2), 183–196. DOI: 10.4202/app.2008.0202.

**Zheng, R., Farke, A. A. & Kim, G.** 2011. A photographic atlas of the pes from a hadrosaurine hadrosaurid dinosaur. *PalArch Journal of Vertebrate Paleontology*, **8**(7), 1–12.

**Zheng, W., Jin, X., Azuma, Y., Wang, Q., Miyata, K. & Xu, X.** 2018. The most basal ankylosaurine dinosaur from the Albian–Cenomanian of China, with implications for the evolution of the tail club. *Scientific Reports,* **8,**3711. DOI: 10.1038/s41598-018-21924-7.

**Zhou, S.** 1984. The Middle Jurassic Dinosaurian fauna from Dashanpu, Zigong, Sichuan, Vol. 2 (Stegosaurs). *Chengdu: Sichuan Scientific & Technical Publishing House*, 52 pp. + 13 pls. [In Chinese].

**Morphological data matrices**

**Soto-Acuña *et al*. (2021)**

xread

383 75

Marasuchus_lilloensis

??????????????????????????????????????????????????????????????????????????????????????????????????????????????????????????????????????????????????????????????????????????????????????????????????????????0?000?00??00000000??000??000000000??00000000?0000000000?0000????1201?0??00?0?000??????????????????????1001000000?0??020000000000000??0000000????0000000000000000000200100000?????100?

Silesaurus_opolensis

0???0?????000?000001??1?0000000????????000?????????00?0000????2???????????????????000??1?0?1?0????0?1????????0??????????00001?1?00?000??0?0???0??00000??0???????????00000000?0???????0?00??00?000002??????001?1000???00??00101?10020000000000?0000100020?00????00?00000????0110000001??000??????????????????????1000000000?0?10100000??000000?00000000?00?000000011?000?00?0?00??0001000?0?100?

Eoraptor_lunensis

00000????0?01010000?102000000?011000000000000????002000000000?000?0000?000000000000?000100??0000100110000000?0??0000000?????????????????0??00?????00000?0????????0?00000000000????0010000000000000020000001?000000000000001010000?100?1001011100001000?0000000?00?0000????0100000001000001000000001000000000?0?0020000000000??00001000?010110??0000000????001?????0000001101000000001100000000?

Herrerasaurus_ischiugualastensis

00000????0?0001000000121000000001000000000000????0100000000000100000000000000000000??000000110000000000?0000?0??000000??000210??0?0000???00?01?0?000000?0????????0?00000000?00????00000000000?0000020?000011000?00??000000001000?0?0000?00000100000000000?0000000?0?0000???0100002000110011?010000101000010000?11100000000?0?0010000000000000?00000000????000000001000000100000000001000000000?

Abrictosaurus_consors

?1000??????11?01??1?00????????010??01???00??10000???????????????????????????1???000??????????????????????????0????????????????????????????????????00000?10?001???0?000010000?10100????00000???10??03?0000000100?0???01011011111101????0???01???????????????????10?00000????????????00??00100010000?010????????0?0211002?00?0?11101100??????????????????????010??1020000?211??????0101100000100?

Fruitadens_haagarorum

???????????????1???1????????????0???????0??????????????????????????????????????????????????????????????????????????????????????????????????????????????????????????00000?00?0?????????????????????03100100??100?00??11111011011100??????01?????0??2000?0??00????????????????????????01?001?????????????????????????????????????????????????????????????????01000112100002111111???????00????00?

Tianyulong_confuciusi

????0????101?001011?00?00?00??1100?0?1??000??????01?????????????????????????????????????????????????????????????????????????????????????????????????????11?000????0100010000?1010001??0?00001?00100400101100100?1???1110101101110??????????????00????????????0?1100000????????????000?1001??0?00?010100001000?010??????????????????????1000?0??11???????????1?????????????1??????01??100000?00?

Heterodontosaurus_tucki

01000????101110111110010010000110010111010001000001000010200001000001010000010000001000200021010100011010000?0??0000001?0000201100000000000001000000000?101001???001000100000101000110000100001000030010110010111100111111111111011?0?1101001000003000000000101100000000?0000000100001100100010000101000010002010201002100?0?1010110000100000?11100001000000100011210000211111?000101100000100?

Pisanosaurus_merti_

????????????????????????????????????????1?????????????????????????????????????????????????????????????????????????????????????????????????????????????????????????000001000?000?00??00?000000?1000?????????11?1?00???000111101???0?????????????000????????????????????????????????????????????????????????????????????????????????????0???????????0?0??????????????????01101000????00?00????00?

Pegomastax_africanus

????????????????????????????????????????????????????????????????????????????????????????????????????????????????????????????????????????????????????????1?1001????010101?0000??00?????????????????????????001011?11?111111111?110??????????????????????????????????????????????????????????????????????????????????????????????????????????????????????????????????????????????????????????????

Echinodon_becklesii

????????????1??1??????????????0?????????1???????????????????????????????????????????????????????????????????????????????????????????????????????????????1????????0100001?00001?10?????????????????????0???001011110011111111101100?????????????????????????????????????????????????????????????????????????????????????????????????????????????????????????????????????????????????????????????

Manidens_condorensis

???????????????1???????????????1?????12?1??????????000010000001000001010000010000001?00?0??2???0??011????????0???00000??00??????0?????????????????00000????????????100010000?101001?100000000?0000????????00100?00??111?10111?110?????1????????00?3?????????????????????????????????????????????????????????????02?10?2100?0?10101100?0?0??????1??????????0????????????????????????????????????

Eocursor_parvus_

?????????????????????????????????????????????????????????????????????????????????????????????????????1?000?0?0????????????0??????????00????????????????????????????00000000??0?10?0??0?10000??0000??????????100?00??110?????0?110???????0101???0??????????????????????????0010000??0000001????????0??0???1??????0?0?????00?0?10?010000000?010??11000?1000100100010200001?10????????0??0?????00?

Lesothosaurus_diagnosticus_

01000????0001000001100100000000100001020001010010100000000000?????0000000000?????00000010001100000010?000000?0??0000000?000010100000000000000??00000000?10100000010000010000?0?1000?000100000010000000010011100?00??11001111011100?000100101???0002?00????00?0111000000???010000?201000000??00000000000?000??1000101002000?0?001?00000000001100110000100?0[0 1]010001020000121010?1?00101100?0??00?

Agilisaurus_louderbacki

01000????0?1100000110000010000010000002?1000110001?000000200002000000000000000010000000110000000000111000000?0??00?0001?0000100?0000010000000??0??00000?10?0?000?000000000001101001?000000000010000101012001100?00??11201111011100100?0?0101110000200000000010110000000???110000100120?001??????????????????????0201002000?0?001000000010001100110000101100010??1020000121010?11?0101100000100?

Yandusaurus_hongheensis_

??????????????????????????????010000???010??????????0000000000?0??00??????????????0?0?0???????????0???????????????????????????????????????????????00000?????????????????????1?????????????????????????????1?101100??11??11110?11?1??????01011?0010????????0??01?????0?0???01000010??20?101???0????????????0????0?????????????????????????????????????????????1?120???00?21?????????0??00????00?

Haya_gravis

01000????0?01?10001?0010010?0011000010??1000100101?000000000001000000110000001010000000?10000100000111000000?0??0000000?000?100?00???00000010????000000?11100010011000000000?1010?1?000000000?0000010001010?1011000?1100111101110?10000001011000003???00?00?1011010000000?0100001000000001000000?0????0?????????0201002000?0?10101110000000?1001100001001110111121200001210?011110001?00000100?

Hexinlusaurus_multidens_

0??00????0??????0????????10??0?10000102?10?0100001?0000000000020000001100000000?0000000??????00000010100?000?0??0000000?000010??0?????????????????00000??????????1???000000?11010?????????????0000????????11101000?0110011110?110010000001011100002000000000101100000000?0110000100000000100000000000000000??20001010?2000?0?10100100000000010111000010110001100102000012101001110101100000100?

Changchunsaurus_parvus

?10?0????0?01?10?0110010010000110002101?1000100??1?0000110000010000000101?0000010000000?1?00???0??011????????0???000000???????0?00?0000000??0??0?000000?111000100110000100001101001?100000000010000100010110101100??1100111101110??00?01010111???03??????????011??0000000??100?010??00?001??????????????????????0??????????????????1????????????????????????111121???????1??0????00?1?0000?100?

Hypsilophodon_foxii_

01000????0?010100011000000000011001010201000100101?000000000002000000010000001010000000210000100000111000000?0??0000000?0000101100000100000?0100?000000?11100010?100000000001101001?1000000000100001000100001010010?1100111101110110000001011100003000000000101110000001000100001000000001000000000000000000?2?00201002000?0?10101110000000010111000010111[0 1]011112120000121010?1110001100000100?

Jeholosaurus_shangyuanensis

01000????100101000110010010000110000102?1000100001?000001000001000000010000001010000000010000100000111000000?0??0000000?0000100100?0000000010?10?000000?11100000010000010000?101001?000000000?0000000001010?101100???100111101110?1000010101??0000300000?00010110000000??0?1?000??0000?001??????????????????????0201002000?0?1010111000000011001100001???1101111212000012101011110101100000100?

Orodromeus_makelai

01000????0?01?10001100?0010000?11?0010211000100001?000010?000020000001?0000000010?000000100??1000?0111?0?000?0???000001?0000?0??000?00??0?????00?000000?1????????1?00000000?1101001?100000000?00000100010?0?10010????10011110??10?10??000101?1000030?000?0001011?000000?0001000010??000001?0?0?0??0???000000???00101002000?0?10101110000000010111000?101110011?121200001210101??100?1100000100?

Yueosaurus_tiantaiensis_

????????????????????????????????????????????????????????????????????????????????????????????????????????????????????????????????????????????????????????????????????????????????????????????????????????????????????????????????????????0101??00?0????00?00010????00???????100001??????0???????????????????????????????????????????????0000????110?????????011??212?0???2?0?0?????????00?????0?

Gideonmantellia_amosanjuanae

??????????????????????????????????????????????????????????????????????????????????????????????????????????????????????????????????????????????????????????????????????????????????????????????????????????????????????????????????????????????00?0????00?00010????000???????????????????????????????????????????0101?????0?0?00?01110???????????1????114???0111120??0001?1?10?1??0?01?00000?00?

Koreanosaurus_boseongensis

?????????????????????????????????????????????????????????????????????????????????????????????????????????????????????????????????????????????????????????????????????????????????????????????????????????????????????????????????????????0?1??0000?????????????????????????20000?0??0???01??????????????????????0????????????????111???????????????????????011112120000??10??????????????????0?

Parksosaurus_warreni

0000?????0?????00????????0??????1?001???1000100??1?00?00000?0??00?0000??00000?0?0?0?0??????0?1?000011???0000?0?????000???0??????0???0?????????????00000???????????100000000?11010?1??00?????0?1000????0???112??100???100111101110????????0???20???30??0?0???3?111000?0?10?020??????02???????????????????????????0??100??00?0??????1??0?000001?111?00?1?41??01???21201001??0?0????00??1????0?00?

Zephyrosaurus_schaffi_

0???0??????010?0?01100??????0011???0???110?010011??00001000000?00?00011000000?????000????0?0?1?00?0?11??0000?0?????0????000???0?000001??0?0????0??00000???????????????0???0?1????????00?0????????001??010?1?1??100??110?11110?110????????1?????????????????????????????????????????????????????????????????????????????????????????????????????????????????????????????????????????????????????

Thescelosaurus_neglectus_

00000????0?010100011000000000?111000002010?0110001?0000000000000000000000000000100010002100?0000000111?00000?0??0000000?0012?001000000000?01??????00000?111000100110000000001101001?100010000000000000?1001110110?0?00001110001100??????0000110000300001?000101110000001?0020000?0?02000010000000000000000??12000101002000?0?1?1?11100000000101110000104111001?12120100121010?1?10001100000100?

Gasparinisaura_cincosaltensis_

0?????????0????00???????????00111000002?1000100001??00000?0000?0000000?00?0000000000010?100????000011???00?0?0???00000??0?00????0????????????????000000??????????10000000000?1?1001??0000??00?1000????????0010111?0?11001111011101?0????0?01???000200?0?????3011100000???0?100?0?0??00?00???????????????????????0301002000?0?10001?10000000010111000?1011100?1112120000??101021110101100100100?

Dryosaurus_altus_

01?00????101?100111110000?0?0011100001201010110001000000000000?100000010000000?100000???00000000000111?00000?0??000000??001?1????00?01??000?0100?000000?1101001001100000000?1101?01?000000000?10001???????1021101101100011111111011?000010011100003000010000101100000000000100?01??020?001000??00???0?????????0?0101002000?0?10001110000101010111000011011000111202001112101021110101110100100?

Zalmoxes_robustus

?00?0????0?10?00011?00001000??100?0000201001?????1?0000001000031010010?00000?0??0000010210020000000?11?00000?0??000000??0?02?00?10?201000???????????????1?00001000000000000011111?1?100010000010001?????????21110110110011110111011?00001111?200103?00????0????1???????????110?000??0?0?01??????????????????????020100201100??0101111001000?0?1????????????101?1202?11??21?1??1?????????????00?

Tenontosaurus_tilletti_

00000????0?100000111100010000010100000201000100101?000000000002001000010000001000000010200020000000111000000?0??000000??00121001000101??000???000000000?110100001110000100001111101?100010000010001???????002110010010001111?1110120001011101200002000011000101110000000000100?01??00000010000000000000110001?000301002000?0?1?101110000001010111000011011010111202011112101021110001110000100?

Iguanodon_atherfieldensis

00000????10100001111100010000020????00201001100000?001000100003100000010000000110000010000000000000111100000?0??000000??0122201110020200000?0200?000000?110100101120010100001111111?000000000010001???????2221101111100011111211112000001?11120010300021000010110100000010011000001000010110111101021011101113100101002000?0?11001110001101011?11100012011110111202011112101021000101?20100100?

Probactrosaurus_gobiensis_

000?0?????010000111110001000002??????0??10?1??????????0001000?3?00000?100?0000???0?0010?????0?00?001???00000?0??0000????????????????????????0??????0000?11010010?1200001000?1111111??00???????10001???????22211011011000111?121112??0??????????0103?0021??00?01??100000010?1?0?0?01000??01????11?1?2?11110111??001010?2000?0?11001111000101011?1110?012011?101112020111?210?0????0101?20100100?

Ouranosaurus_nigeriensis_

000?0????101000001111000100000200000002010011000000??10001000031000000100000001??000010000??1?00000111?00000?0??00000???01220???0??????????????????0000?110???101?200101000??111111?100000000010001???????22?11????11???????121?112?00?0??111200103?0021?10?101??100000????1?0000010?000011?1?1101??111???1???1?01010?2000?0?11001111000101011?1110?01201111011?2020111?210?0??1?0101?20100100?

Camptosaurus_dispar

00000????1011??001111?0??0000010????002?10??10000000000000000031000000100000000?0000010?10??00000001?1100000?0??00000???0010100?000202?00?????????00000???????????1000010000?11110??100000000010001???????112110110110001111121101100?001?111?00102?0021?00030110100000???010000101020000110111101010111100012100101002000?0?10001110001101011?1100001101111011?202011?1210?0??100101?10000100?

Stenopelix_valdensis_

??????????????????????????????????????????????????????????????????????????????????????????????????????????????????????????????????????????????????????????????????????????????????????????????????????????????????????????????????????????????????31000???0?1?????00000????00???0???????????????????????????????021100211100??0111100??2010?0??1?1???100???001??20?????1?????????000??00000?00?

Yinlong_downsi

221111100000100000100010010000011100111010001001101210001001000012000010001100010001100000021010010001010000?10101001100101?100010?10211001011000000000?10100011010?10000000?1010001101000011100100311012000100?00??1100111101110?100001010110001031000000001011000000????00000000000?0001000000?0000000000?????02110011001001021110000101000??1?1?0010200?011?120200001210?0?1??0001?00000100?

Hualianceratops_wucaiwanensis

???????????????????????????????11???11???0?0?????0121000101100101200000010110?0?00000010???0????????0????????????10?1100???????????????????0????????????1?100?1101??10001001?1010?01?010??0????01?????????001?0?0????10?111?0?11?????????????????????????????????????????????????????????????????????????????????????????????????????????????????????????????????????????????????0????00???????

Chaoyangsaurus_youngi

221?11?01??0??00??1001?1?????00?????????11??????????10001011???01200??????????0?0001?0?0???2??????0??????????????????????????????????????0??1?????00000?10000011010?10000000110100???01000011100100410010100100?000?11001111011100??00000?01??????????????????1?????0????????0?0????0???01?????????????????????????????????????????????????????????????????????????????????????????????????????

Xuanhuaceratops_niei

??????????????????10????????????????????????????????????1???0???1200?0???????0010001?0?0???2??1????????????????????????????????????????????????????????????????????012??1????????????0??000111??1?05????????1?0?0000?1??1?1?001100????????????????3????????????????????????0?000???????1???????????????????????????????????????????????????????????????????0?1?121??0??????????????????????????

Liaoceratops_yanzigouensis

210?11100000?00010100010010001110101111?10001001101000000200112000100000101100111001000000021010110101111000?111211000101011110110010110111?11100000000?10101011010010000000110110??010001001100200310012101111111101100111100110?????????????????????????????1???0000?????????????????????????????????????????????????????????????????????????????????????????????????????????????????????????

Aquilops_americanus

?10?11110102??00?01?0010000001210001111?100?100111?00000000011?????000001001??????0???0?????1?10??0?0?1???????????????????????????????????????????0?????111000??0110100000????????????????????????03100121001?11111111001111011102?????????????????????????????????????????????????????????????????????????????????????????????????????????????????????????????????????????????????????????????

Yamaceratops_dorngobiensis

2???111?0?????????????????????21?10111?01000?????01000100200010000110000100100111101100??00?101011010??1??00?1???110?01?101??11?1??101?0111?11?0?000000?1?100????11012000111?101101??00101001000200?1?01?0??1111111011001111001102???????????????????????1????????0000??????????????????????????????????????????0211???????0??0211110??????????????????????????????????????????????????0???????

Archaeoceratops_oshimai

2101111101021000?0100010000001210101111?100010011012000012001?0002??00?01001001111010002?0021010110101?????0????21?00???10??1?111?010?1??1101??0?000000?11101011010010001000110110???101010010002003100121001111111011001111111101????0?0?0??0?000310?????????????0000??????????????????????????????????????????021100100110?10211110??0000????11100010200???1?121??????????0?11?00?1?000001???

Auroraceratops_rugosus

210111110102100000100010000001111101111?100010011012001012001100021100001001001?1101100000021010110101111100?111211000??101111111??10???1110?110?000000?1110001101001001100011011001110101001000200310012100111111101100111101110?211?010101111010310021010110110000000001010000100000000100000000000000000?1100020100101110?10211110?0110000?111100010200001111212010012101021??00?1?100001???

Koreaceratops_hwaseongensis

???????????????????????????????????????????????????????????????????????????????????????????????????????????????????????????????????????????????????????????????????????????????????????????????????????????????????????????????????????????????????????1010110?????????????????????????????????????????????????????????????????????????0100???1?????????????????????????2?010211100??1100????0?

Albalophosaurus_yamaguchiorum

???????????????????????????????1????????0???100?1?????????????????????????????????0??????????0??????????????????????????00??????????02??????????????????????????????????0???10?10??????????????0????????????1011110011??1111011101?????????????????????????????????????????????????????????????????????????????????????????????????????????????????????????????????????????????????????????????

Protoceratops_andrewsi

2101111110?21000001000110010012111011120100?1001101210101200012000110000100100111101100000021010110001[0 1]11100?1112100001?10??111110?101??11101??0?000000?111010110110100101111101101??00001101000200410011?0011111110110011111111022?1??10101?00010310021010100110000000??1010000001?00000100000000000000000012100201001000?0?1021111000100000?111100010?001011?1212000012101021110001111000100?

Bagaceratops_rozhdestvenskyi

21011?1100?2?00000100011001001211101112?100?100110121010020?010000110000100100111101100200021010110001?11100?1111100001?101?111110?101?01?1?1010?000000?111010?1011010010000?101101???0001101000201???????001111111011001111111102????????????????3???????????????????0??????????????????????????????????????????????????????????????0???????????????????????1??21?????????????????????????????

Leptoceratops_gracilis_

2101111100?2?00000100010000001211101111?100?100??012101012000100001100001001001111011002?0021010110001110000?1112110001?101?1[0 1]111??101??111011?0?000000?11101011011012010011?101101??10001001000201???????1111111110110011111111012?1???01011000103???21?1010011000000000100?000111000000100000000010100000011000201001000?0?10211110001000?0??11100010?001111?121200101210?02?1?0001110000100?

Psittacosaurus_mongoliensis

221110001112100010100121000100?0???????0110010011??210100011001010100011000000011000001010020000010001110000?1010000001?10?000111002000001101100?000000?10000001011010000111?1011001?010100?0000101???????001111110011001111011101?00001010110000030000?00?0101100000000010100000100000001000000?0000000000010?00211001000?0??021111000000000?111100010200101101212?010121?10?1110001100000100?

Psittacosaurus_lujiatunensis

22111000111210001010012100010000????????110?10011??210100011001010100011000000011000001010020000010001110000?1010000001?1010001110?2000001101100?000000?1000000101101000011111011011?010100?0000101???????001111?1001100111101110???0??????????????????????????????????????????????????????????????????????????????????????????????????????????????????????????????????????????????????????????

Mosaiceratops_azumai

21011011?0?21000001?0?110?01?1211101111?10???????01200001001??00001000010?01??1?1?????????????????0???????????????????????????111??20???????????????????1110101101?010010011?1011?01??0??1001?00201???????????1?111???0?111???11??????????????????????????????????????????????????????????????????????????????????01001????????21???????1??????????????????01?????2?????????????????????????00?

Scelidosaurus_harrisonii

01000????0?010000011001000000001100010201000101??0010000020010200000000010000000000100010001100000010100000000??000000??000?100101010000000?0?01??10000??????????100010000001001001?000100000?01000110010011100?00??1100111101110000001001001200002000000000101100100100?00100000210000001??000001000000100002000101002010?0?0000000000000000?1110010100001010001020100121010111000011110001110

Scutellosaurus_lawleri_

???00???????1????0?100??????????????????00?????????100000??000?00000????????????0?0???0????1???00???0???????????????????????????0?????????????????1???????????????0??000000?10?????????????????00?00????0?10100?00??11?01111011100100???000??100?02???0?1?00?0?1??10000??????000?2?000?001???0????0?????0?0????001?1002000?0??010?00?00?000????110000100?010100010201001?10?0????0????000???100

Emausaurus_ernsti_

010?0????0?0?00000?100000?0000?110001020100010000001000002?000100000000010000?00000??0??????100000010???0?00?0??00?00????????????0??00????????????10000??????????1001100000?10?10000000100000?000001?0010???100?00??11001111011100??????????1???????????????10????100?0??????????????????1???0????????????????????????????????????????????????????????????1????????????????????????????????1?0?

Gargoyleosaurus_parkpinorum

1???0??????0???????????00?0000?0???????????????????????????????????00????????????01?0001???1??????010??00????0??????????????0???????????????????00111221??????????0???1?????????00???0?????????100???0????????00????????????011?00?????????????????????????0??????10?1????????????1??0??????????????????????????1???1???1????0??000???????????????11???????1???????????1?????????11?????????2??

Pinacosaurus_grangeri

110?0????0?11000001100?000?01010????????1000121?????000002???0?00?0000??000?00??101??0011101?0?10?1??1?0?010?0???0?00???00100???010000??000?0101111112111?00010??110?010000??0??001?000000000?0100????????0???1?????????????011?00??01?0?1011??001??10???000211???10110????2?111?11000?001??0?0011?001?1??1?????12??1?201????0??000???0???????????11???????1?0????0????121???????1111???0??1?1?

Euoplocephalus_tutus

1??????????11000?01100?0000010?0????????10??1?1?????000002?0????0?0000????0?00?0101??001110????10?1????????0?0??00?00???00100?0?01??000000??0?0111111221?????1????10?01???????????1????????????1??????????22??00???????????????????101??????????????1??????0?1?????????????21?1?????0?????????????????????????????0???????????????????????????????????????????????0???????????????????21??????0

Huayangosaurus_taibaii

00000????0?0101000110011010000110000102?1000121??001000002?000200?00000010000001001??00100111000000001100000?0??00?000??000010??0?0?00???00?0?00?000000?1000000?01000100000??0010001000000000?10000000010022100?00??1100111101110?11??0?01011??1012?00?1?101?011??110?1????111001??0?0?01101???????????1????????0211112010?0?00000000?0????????11?011100011100?0??0??00121??0??1?1111?21?11110?

Hesperosaurus_mjosi

0????????0?????00???????0000?0?1????????10??1?1????1??0002?00020000000??0?0?00010?1?????1???10?0000?01?000?0?0??0??000??00???????1??????????????????????????????????????00?0?0?10001?0?000000?1000??????????1101????11??1?1?0?11??2000000?01?001012??0210?11?0110011????????????0?1??0??????????????????????????0??1112010?0?0000000??00??2?0??11001?100???1???????????1?????????11?????????10?

Stegosaurus_stenops_

00000????0?0?00000111010100000210?00?0201000121??0010000020000200000000000000001001?000110110000000001?00000?0??00?000??000?1011010200??000?00?10000000?1000000?012001000000?0?10001000000000?10001???????221101100001??1?1?0111002100000?0012010120002101111011001100000?011010021000??1111000011000102??1?11000211112010?0?000000000000020??111001111001110000200??0?121??0??111110?21111110?

Wannanosaurus_yansiensis_

???0???????????????????????????????????????????????000001???00????0000001?12???????????????????00?0?0110?0010100110011?10?????????????????????????00000?1????????00?0000000??100001?000010??0?0020?????????0110?00??01??11110?110???????01????????????????????????0000?????????????110?100??????????????????????????????1??????????????????????????????????0010?21101???2?1??????????????????0?

Homalocephale_calathocercos

0??00?????????????????????0?????????????10??111????0000012001020000000001112001100010000100000000001111000110100110111?1000110101011101110110??0?000000?????????????????????????????????????????????????????1?010?0??1??11110?1101????????????1000310110??00?011110000001???????????????????????????????????????0201001111011?120111011100000?11111001131120???1??100?012?1?0????0????0?????00?

Goyocephale_lattimorei

0???0????????0?1?0???????00?????????????????111???????????????????000????1?2????????????????0000000?1110000101001??011?1????????????10??1??????????0000??????????00?00001001?10?00???000?0?00?0021030001?0111001000?012?1111011101????????????????1??1????00?01?11??0?0????????????11??101??????????????????????02?1001111010?12011????????????????????????????????????????????110????00????00?

Stegoceras_validum

010?0????0?0?0000011000000000001???????01000111????0000012001020000000001112001100010000000?0??10001111000111100110011?1000110?01?1110111?100210?000000??????????0??0000100??100001?0010?0100000?10300010011100100??112?1111011101????????????1000????10?000?0111100000????1?000?0?11??101??????????????????????0201001?11011?12?1110111000?0??????????????0??????1?00??2?1???????????00????00?

Prenocephale_prenes

000?0????0?01?0100110000000?0011????00?01000111??1?00000120010200000000011120011000100001000???1??01111000111100110111?10001100010111011101?0110?000000???????????????????????????????????????????03?00?1?1?1?0??????12?11110?1101????????????1???31?11???????1?1???0?0??????????????????????????????????????????2?1001111011?12??110111000?0??????????????0?1????1?0????????????????????????0?

Micropachycephalosaurus_hongtuyanensis

??????????????????????????????????????????????????????????????????????????????????0100001??1?1?0??????????0000??????????00??????????????????????????????????????????????????????????????????????????????????100??0??01??11110?110???????????????????????????????????????????????????????????????????????????????0??1????00?????????????????????????????????0?1?121??0?0??1??????????????????00?

Laquintasaura_venezuelae

??????????????????1????????????1000??02?0??????????0??00000000???????00?0?????????0?0?????0?????????????????????????????????????????0?????????????????????????????????????????????????????????????00???0????000000??11??1?1?1?110???0??0??????000???????????0??????????????100??0???????????????????????????????02?1?0??00??????0000?0?0010100111000?1??0??????010200001???11?1?????????????00?

Isaberrysaura_mollensis

010??????0?0??100????????10???0100?010??10001110001?000000000??01?000??00????00?00?????????????0??000????????????????????????????????????????????????????????????????1??0?????????????????????????0000010122100?0???11??11110?110??????????????????????????????????????????????????????????????????????????????????????????????????????????????????????????????????????????????????????????????

Kunbarrasaurus_ieversi

1?????????????????????????0????0????????????101????????????????????0?????????????0???00????1??????1??1100????0???????????00000?000?20?????????????100210??????????????1?????????00??????????????00??????????1011?????1????110?1100?????????????001??10???002?0111010110??????0?1??1??0??11???????????????????????1??1?201????0???00???0???????????110?0??011?0?????????1?????????11?????????421

Stegouros_elengassen

???00??????01?0??010????????????????????1???1?1??1???????????????????????????????????????????????????1100???????????????0000?010000200????????????10????1?00000111101111000000010??????????????????????????1101100???1001?110?1100??0?0001010?0001200010000210??101?1??01????0???1100?00110?00000100010???1????00211112010?0?0000000100100200??????????????10000200?000121010200011011210001421

Antarctopelta_oliveroi

????????????????????????????????????????????????????????????????????????????????????????????????????????????????????????????????????????????????????????????????????????0???0???????????????????????????????101100???1????110?1100??0?0?010????????????????2?0??101?1????????0???????????????????????????????????????????????????????????????????????????????????????????????????111112?????4?1

Jakapil_kaniukura

???????????0???0?01?00????????10???????01??????????????????????????????????????????????1???1???????????????????????????????????????2?1?1????????????????1?1000010110000001?011??001??0100???0?00??1????????0000?00??110???1?01110?????????????00?0?????????????0??1011????011?100???00????????????0????????????????????????????????????000200?1????????????0?????????00???????????????11???????

;

ccode + 1 22 30 38 124 162 195 202 203 221 226 237 242 246 267 291 295 301 305 319 360 *;

proc /;

comments 0

;

**Norman (2020b)**

xread

115 19

Silesaurus 000000000000000000000000000?000??00000--00000000001000000000000010?0???00000000000000000000101001000000000000000000

Lesothosaurus 0000100000100000000000100001200100000000000000000010001000000020001000010110000000101011100102001010001000000000000

Hypsilophodon 0000100001100000000000010002201100000000001101300010012000000020001000010110300110101012100102001010011000000000000

Dryosaurus 00002000111000000000000100012011000000100011013020100120000000200010???10110311110101011101102001010011000000000000

Scutellosaurus 0?00?0??01?010001?100010000?101?????????0??1????001000100?0?0?201010?00111100000??10101??00102111010001111?1??10100

Emausaurus 000010??012110001?10001000??11???1??????11?10?1000100010???1??????2????????????????????????????????????111????10100

Scelidosaurus 0000100011211100211?00100001111101011011111101110010001000010020112010011210000010101011110102111110001111112110100

Huayangosaurus 00001000012100000?0000?0100111110?00?1100111001000111010?0000010?1111???021110???11020111101111121200?11020?0011001

Kentrosaurus 00???????1??00?00???00?010??11110000????01?110???122101000001?11011111210211100?111020111102111121200??102000?11001

Stegosaurus 0000200011210000000000101000111200001110011110102122101000001011012111210211100011102011110211112120020102000011000

Kunbarrasaurus 010021112121111?21??12200001?11?00001?211??11120212020??0?010?1??1?????0111??000??1010232?031????????11111111110100

Sauropelta

011??????1??111131??11???11-?1??1?1????112?11112?122201011010??2112012201221221???1131232103?1112120121111132?10100

Struthiosaurus 011??????1??11?131?111??????????1?1?????12?1??????2220??0?110?12?0?0????1???221????????????31111?1?0?2?11?112?1010?

Silvisaurus 0110100121?111113121112001131101111012211201112?0122201111?10????????12????1?2???????????103211121???2?111132??0100

Edmontonia 0110200121211111312111200113110111101221120111222122201111110112112??2201221221222113123210321112121121111132110100

Jinyunpelta 01?1211?112111??2????0200003?1????????211?0111?12???????1???0?10??201??0111??00??????????1?321112120??1111??1?10010

Pinacosaurus 1101211121211122312112200013110101001221120111122121201111110110112012201221211222113123212320212121121111131110010

Euoplocephalus 1101211121211122312112200013110101001221120111122121201111110110112012201221211222113123212320212121121111131110010

Jakapil ????2????1??????????????????1??????1??0001011110?010001?0??10?100????????????00?1????????1?1??11??1??001111????????

;

proc /;

comments 0

;

**Wiersma & Irmis (2018)**

xread

293 36

Lesothosaurus_diagnosticus 0000000000000000000000000000?00000000000000????00?0000000000??0????00000000000000000000010?0000000000?????????????????????00000100?0100000000000000100000000000000?0000100000??00?000000000000000000000010000?1100010?00000000?0?????????????????????????????????????????????00000000000000??1???????

Scutellosaurus_lawleri 0???????????????0??0000???00?1??????????????????0????????00???0?01?0000?????????0?00000????????000000?????????????????00??00000010?0000000000000000102000000000000?00000000001000?00000000?000000100000010000000000000000100000000110?????????????????????00??0000000????0???000000000000000?101????0

Emausaurus_ernsti 1100000000??00000??0000???00?10000010000000????00?0000??0???????00?00000?????????011101010?0000000000?????????????????00???00??????????000????????????00000000000??????????????????????????0???????????????????????????????????000100?????????????????????1????0?????????????000??????0??????????????

Huayangosaurus_taibaii 003001?000000000000000000000?10000021200000????00?0000?0011100001000001100?100020001102000?0000011000?????????????????10??01010000?0011001101000200201???000000000?00101100001011010001000?1110011010000000000????0110001000000100100?????????????????????120010??00?0???0???100100000?110010000[0 1]0[0 1]01

Tuojiangosaurus_multispinus 003000000000?000000???????00?1?????21?00000????00?0000?????1000?000?011100????????01000????????011000?????????????????10??000110???1?1?00?101000201100000???0????0?0021110000201101?01000?1?111011010000000000????01?010100000??00100?????????????????????10??10??00?????????011?111000111010000[0 1]0???

Jakapil ?????????0??????0?00??????00?1???????????????????????????00?????????????0?1??????0100011?1??111100???????????0?????????1?????00?00?000??01???????????????????????2?0001101??????????00?0??????????????????????0001?????0???????0?01?1????????????????????01???????????????????00?000???????????1?????

Scelidosaurus_harrisonii 0000000000001000000000000000?10000021101010????00?00000000000??00000000000?00000010100111101100010001??0??00000?000000110000000000?00000011?20??00011000100?000011?0001000000???000011000010010101000011100001000100000010100000001110000000000000000010011100100000010000???000000000000000?101????0

Minmi_paravertebra 10010000000020100???0?00100??2010002110101110100200002?0000011000000001000?0000???01??01110?1??010011??0???0?00?00000011000??1?????000110110??00????????1?0?0??011?0000?01?0?????0001000001?01110100001110000100110000???0?000?000111010?00?1?0000?000100110??010000010000????000?000??00000?101????0

Mymoorapelta_maysi ?????00?0????0?????????????????????????1?111010020110200?0011?0?01000011000??????????????????????1?121111?????????1???1???00?11110?00000011020011111200210010010?2100??1????01??1?11110001?00101110100111000111221????????1000?00011111100011000??????10011101000012010000???????000000000?0?101????0

Gargoyleosaurus_parkpinorum 1001000000002010010001001000?201100212010111010020110200???1100001000011000000???1011201110110100111211111100010001000110100111110???????1???00011?1?002100?0?10???????1?0100??11?1?11000??0????????0011100011????121010???????00011111100011000??????100111010000120100?????000?0???00????0??01????0

Europelta_carbonensis 103001?00???2111??????????0102010002120101000000110002101111110000000011111??0???101120?????1?001011210000???00?0101001101010111011101001112212111021211100101100??????0?1?1?1?00?1?11?0???111010111111121112100111110101011000100111?1???0????0????0?????1100001011111000???0000000000????0?101????0

Cedarpelta_bilbeyhallorum 1010?1?0?00021110??001001001?2?????21201010000001100000001101???00000001111000110??????0110110??111120000?11?00?0?000011?????11000?1000???1111?1110220001???0?????????????????????11111000????????????????????00000110101011????????????????????????????????????????????????????0000000??????????????

Gobisaurus_domoculus 2010001010012012020201101001021111021201110000011100100101110100010?001110100011?????????????11?01112000??10000?0001002??????????????????????????????????????????????????????????????????????????????????????????????????????????????????????????????????????????????????????????????????????????????

Zhongyuansaurus_lauyangensis 1011001011012012020201101001021111021201110000011100???????001010100001110100????????????????11??2112000??1100100010002?????????????100??????????????????112?1111?????????????????1????????1??????????????????0000?????????????10010???????????????????????????0??????????????????000??????????1?????

Shamosaurus_scutatus 2001001011012012020201101001021111021201110000011100100101100101000?0011101000?1?1111?0111011111??112000??1000100011002101?????????1?1?01???????????????????????11?00010001100112???????????????????????????????????1011????????0010211100111001??????0??000??00?????????????0000?000??0000????1?????

Tsagantegia_longicranialis 100100201101201212021110100102111102120111000001110011110??0010101000011101000100????????????11?11112121011100100001002?????????????????????????????????????????????????????????????????????????????????????????????????????????????????????????????????????????????????????????0????????????????????

Crichtonsaurus_benxiensis 11000010100?201212021??0100??211110212111111100321001?001??01110001?0111101000?00????????????11???112121111100100001002??????11000?101?011101100101110111???0????210001001?100112?1?111101??11110211??????????????021011211111??0010212200122001??????0??010??000000010111000????00000000000?101????0

Zaraapelta_nomadis 310000101???2012??????????0??21111?21201011110032100100011010111001?0111101????0?????????????????211212201?????00001102?????????????????????????????????????????????????????????????????????????????????????????????????????????????????????????????????????????????????????????0????????????????????

Pinacosaurus_mephistocephalus 32000020120120121202111010?112111?021211111110032100?0??111??????????11????000?00??111?00100111?12112122111111100000002101?1???????????01?1021??1001????01121?111?????100001??????11??10?01?111102?1????????????????????????????0010212200122001??????0??000??000000?????100??00?????0?????0?101????0

Tianzhenosaurus_youngi 310000201001201212021110100112111?121211?1111003210011001111001200?0011110?000100??11??00100?11?1211213321?111100001002???01111?00?1?0?01110210010012???011211111210001001?10011211???11001111110?1?111120002100000?1?11?1?111?1???????????????????????????0??0?0000?????100??00000000?0000????1????0

Pinacosaurus_grangeri 3101001?1201201212021110100112111112121111111002210011101110011100000011101000100111110001001111121121331111111000000021010??1?200?101?011111100100120??011211111210001001?100112111001000111111021011??200021????021011?1?111110010212210122101??????0??000??000000?10111011000000000000000?101????0

Saichania_chulsanensis 3101001012112012120211101001121111121211111110032100111111110011001001111010001001111100011011111211212311111110000100210111111210?1?01011102100100120100112111112100010001100112111001000111111021111112111210000021011211111110011210000000000??????0??100??000000010111001000000000000000?101????0

Ahshislepelta_minor ?????????????????????????????????????????????????????????????????????????????????????????????????????????????????????????????????????????????????????????????????210001101?101111?10000000??????????????????????????????????????0011??????????????????????0????????????????????????????0000??????????

Tarchia_gigantea 3101001012112012120211101001121111121201111110032100111011110111?0?00111101000?0011111000100111112112133111111000001002101??????????????????????????????011211111????????????????????????????????2??1???211121??????????????????0022????????????????????????????00000101111200000??????????0??01????0

Scolosaurus_cutleri ?????????????????????????????????????????????????????????????????????????????????????????????????????????????????????????????11100?10????1111100?0?110000???1?1????????101?111111?11001100110101021011112111210001011011211111110021212210122100??????0??000??00000000???????????0???00????0?101?????

Shanxia_tianzhenensis ?????0101?????12??????????????????????11?111100321?????????1?112001000111?????????????????????????1121??21???????00???2???0??11110?011????????????????????????????????????????????????????????????????????????????021011????????00????????????????????????0????0?????????????????0000??????????1?????

Akainacephalus_johnsoni 2200101010112012120211101001121111121211011111022100110001110112000001111010001?0111110001001111?2112133211111100001002111???0021??0110011101110101110000112111102100011001112111?11101100??11110210??????????????0110111???????0021212210122201??????0??000??00?????0???1011000000000000000?101????0

Nodocephalosaurus_kirtlandensis 31001010101120121?02111??00112?11??2121101111103210010000101????001001111????????????????????1???2112133211111100001002????????????????????????????11110????????????????????????????????????????????????????????????????????????0022??????????????????????0?????????????????????0????00????????1?????

Minotaurasaurus_ramachandrani 31011010021120121202111010011211111212111111100321001000111111120010001110100010011111000110111112112133211111100001112111111???????????????????????????????????????????????????????????????????????????????????????????????????00??2?22001??????????????????????????????????0000????????????????????

Tarchia_kielanae ??0??0101????01???????????????????????01?1111001?????1?????00112???0001???????????????????????????1?213?2????????00??12??????????????????????????????????????????????????????????????????????????????????????????????????????????????????????????????????????????????????????????????????????????????

Anodontosaurus_lambei 2201102012112012120211101001?211111212011110100221?0110011110111000?0111101000?00111?1?00100111?12112111011101100001102111??????????????1?1?1?0020011000?112111?1???????????????????001?????1111021???????????0201011011????????00212122?0022?00??????0??100??00000000???1121?000????00????0?101????0

Oohkotokia_horneri 2201101012112012120211101001121111121211111110032100110?11?10112000?01111010?0???????????????1???2112111011101200001102??????11???????????1?110021011011????1????21000???1??????????????????????????????????????????????????????0021212110021100??????0??000??0??????0???1011???00???00????????1????0

Euoplocephalus_tutus 220110201211201212021110100112111112121111111003210011001111011200000111101000100111110001101111121121110111112000010021110??11110?100001111110020112000011211111210001100111?11111100100?111111021111112111210000011011211111?10021210000000000??????0??000??00000000???1011000000000000000?101????0

Dyoplosaurus_acutosquameus ??0??0101?????1????????????????????????1?1????????????????????????????????????????????????????????11211?01???????00???????????????????????1?11??20112000011211111?????????????????1?????0??101010211??????????????1110112111111?0021??????????????????????0????00000010111000????????00????0?101????0

Ankylosaurus_magniventris 32011010101120121202111010011211111212011111100321001000111101110000011110100010011111000100111112112111011100200001002111???11110?1010011??????20111000011211111210001101?111????1?10100??1????0?????????????0001111011???1???10010211??0010001??????0??000??00000001011101100000000000000????1????0

Ziapelta_sanjuanensis 110?10101211201212021110100112111?121201011111032100???????101110000011110?000???????????????11??2112111011001200001002?????????????????????????????????????????????????????????????????????????????????????????????????????????0021210000022200??????0??100????????????????????0????00????????1?????

;

proc /;

comments 0

;

**Maidment *et al*. (2020)**

nstates cont ;

nstates 32 ;

xread

115 26

&[cont]

Lesothosaurus 0.5 16.0 6.0 0.1 9.0 1.0 3.2 3.2 14.0 ? 2.8 0.2 0.5 0.2 0.57 0.64 0.17 0.8 2.1 ? 2.7 1.6 0.78 5.3

Scutellosaurus ? 18.0 4.0 0.1 7.0 1.0 3.0 3.0 ? 0.8 2.0 0.2 0.6 ? ? 0.83 0.23 ? ? ? ? 1.2 0.87 5.5

Emausaurus 0.59 19.0 6.0 0.65 ? ? ? ? ? ? ? ? ? ? ? ? ? ? ? ? ? ? ? ?

Scelidosaurus 0.5 ? 7.0 0.4 8.0 1.07 2.46 2.56 16.0 0.6 2.68 0.35 ? ? ? ? ? 0.8 2.4 0.25 1.9 ? 1.11 ?

Huayangosaurus 0.8 21.0 6.0 0.5 8.0 ? ? 1.6 16.0 1.25 2.7 0.4 0.8 ? ? ? ? 1.7 2.11 ? 2.0 1.3 ? 7.4

D._armatus ? ? ? ? ? 2.6 0.8 2.2 ? ? 1.9 0.3 0.6 0.3 0.78 0.7 ? 1.6 ? 0.7 2.1 1.4 ? 1.4

Miragaia ? ? 5.0 0.2 17.0 1.14 1.53 1.8 ? ? 2.0 0.4 ? 0.5 0.71 ? ? 1.5 ? ? ? ? ? ?

Loricatosaurus ? ? ? ? ? 1.91 0.99 1.9 ? ? 2.06 0.35 ? 0.3 0.94 ? ? ? ? ? ? 1.7 ? ?

Kentrosaurus ? ? ? ? ? 1.27 0.45 0.62 ? 1.1 2.5 0.46 0.9 0.3 0.95 0.72 ? 1.6 4.57 1.1 1.6 1.69 1.47 1.45

Paranthodon ? ? 6.0 ? ? ? ? ? ? ? ? ? ? ? ? ? ? ? ? ? ? ? ? ?

Chungkingosaurus ? ? ? ? ? 3.2 1.1 3.6 ? ? ? ? ? ? ? ? ? 1.4 4.2 ? ? ? ? ?

Tuojiangosaurus ? ? ? ? ? ? ? ? ? ? ? ? ? ? ? ? ? 2.28 3.6 ? ? ? ? ?

Gigantspinosaurus ? 30.0 ? ? ? ? ? ? 16.0 ? ? 0.36 ? ? 0.8 0.7 ? ? ? ? ? 1.6 1.57 ?

S._homheni ? ? ? ? ? 3.2 0.9 2.7 ? ? ? ? ? ? ? ? ? 1.5 9.3 ? ? ? ? ?

S._stenops 0.3 23.0 6.0 0.3 11.0 3.52 0.8 2.5 14.0 1.6 2.1 0.43 0.97 0.41 0.94 0.67 ? 1.81 6.1 0.71 2.05 1.94 1.72 1.1

Hesperosaurus ? ? ? ? 13.0 1.37 1.73 2.51 16.0 ? 2.3 0.5 1.0 0.4 0.93 ? ? 1.48 4.42 0.7 1.8 1.8 1.8 ?

Gastonia ? ? ? ? 8.0 1.0 3.1 3.1 ? ? 2.5 0.5 1.1 0.6 0.81 ? ? 1.3 ? ? ? 1.9 ? ?

Sauropelta ? ? 5.0 ? 8.0 1.0 1.95 1.95 16.0 0.99 2.9 0.3 ? 0.5 0.8 0.82 0.23 ? ? ? ? 1.5 1.3 ?

Euoplocephalus ? 21.0 6.0 ? 8.0 1.0 2.83 2.83 ? 0.9 2.5 0.3 1.4 0.4 0.83 0.58 ? 2.4 2.8 2.5 0.2 ? ? ?

Jiangjunosaurus ? 21.0 7.0 ? 11.0 ? ? ? ? ? ? ? ? ? ? ? ? ? ? ? ? ? ? ?

Laquintasaura ? ? 4.0 ? ? ? ? ? ? ? ? ? ? ? ? ? ? ? ? ? ? ? ? ?

Alcovasaurus_longispinus ? ? ? ? 8.0 ? ? ? ? ? ? ? ? ? ? ? ? 1.8 ? ? ? ? ? ?

Pisanosaurus ? 15.0 ? ? ? ? ? ? ? ? ? ? ? ? ? ? ? ? ? ? ? ? ? 8.3

Moroccan_stegosaur ? ? ? ? ? 1.45 1.47 2.14 ? ? 1.92 0.32 ? ? ? ? ? ? ? ? ? ? ? ?

Isaberrysaura ? 30 7 ? 6 ? ? ? 15 ? ? ? ? ? ? ? ? ? ? ? ? ? ? ?

Jakapil ? 11.0 7.0 ? ? 1.0 1.6 1.6 ? ? ? ? ? ? ? ? ? ? ? ? ? ? ? ?

&[num]

Lesothosaurus 0??00?000??0000?00?000001000?111?00000?1???1010?10000100000000?0?0111??00000100100?00000???

Scutellosaurus ???01??00?????0????00101?0010011??0?00??00?????1?0000?000?000100?0110??000001?01???10000??0

Emausaurus 00??1000????????0??00100?00?????????????00?00???1?????????0????????????????????????10000???

Scelidosaurus ?1?010?10100100100100100?0010101000?01?100?10100100001000?000110101110000010000000010100??0

Huayangosaurus 01101101111010111010010010010???001001?000?00110100011?1?011011000001000??11011?1??1100?000

D._armatus ???????????????????????1????????1???111?11011??????????1???0111010?010100101121????110??0?1

Miragaia ?0001????????????????????????1011?????????????????1?10?100??????????????????1??????11????0?

Loricatosaurus ?????????????????????????????000001?01??2111?001?0?????1?????110????1??01101021????11??11??

Kentrosaurus ????????01??????1??101??1????101?01?01011101111011001101101011100010?1101101021?12211001001

Paranthodon ?1??0???????????????0?11?11????????????????????????????????????????????????????????????????

Chungkingosaurus ???????????????????????0??????????11012????10???10???????0?1011000?0110?1??????????11?0?00?

Tuojiangosaurus ????0?1?0??????11??10111??1??????????????????????????????0?1111000?0100????????????11??????

Gigantspinosaurus ?????????????????0?011??1?1???????1?002100?10?10??000?01???0111000?0100??1???21????11001000

S._homheni ????????????????????????????????????0110??????????????????1111111100101????????????11???1??

S._stenops 000110011111101011011001?11100000100012011011011011010010?111111110010101101021112211000111

Hesperosaurus 0???1101111110101???????01?10111011001?011011??111101?11??111111110010101101021?1??110001?0

Gastonia 1??11??10000?11??????????1011????01?012000?1010110?1010000?0111000?0100111???11????10110???

Sauropelta 1???1??10000111??0?00111?1111????01?012000?00101100111000110?110000010001110?01000110110??0

Euoplocephalus 10?11??10000111?10?00111?1111????01?0120?????1011001011011111110000010010?01011110210110???

Jiangjunosaurus ????1????111??11?0100101011??111???0????????????????0??????????????????????????????1100??1?

Laquintasaura ??????????????????????00??0??01??????0????????????0?0???????????????????101????????00??????

Alcovasaurus_longispinus ????????????????????????????????????0???211?1??000??????????????????????11??0?0????1???0???

Pisanosaurus ????0?????????????10?0?1????????10???????????????????????????0??????0???????????0000000????

Moroccan_stegosaur ????????????????????????????????01101??????????????????1???????????????????????????1???????

Isaberrysaura 000?1001???????1??????00?01?????????????????????????????????????0??????????????????????????

Jakapil ????1???????1????1100000110?????00?001???????11???001?????0?????????????01?????????1010???1

;

proc /;

comments 0

;
